# Isocyanoalkynes: Leveraging a High-Energy Interstellar Lead for Unique Multi-Bond-Forming Cascades

**DOI:** 10.1021/jacs.6c12613

**Published:** 2026-09-23

**Authors:** Huan Tian, Tore Brinck, Christopher Absil, Fraser F. Fleming

**Affiliations:** † Department of Chemistry, 6527 Drexel University, 3401 Chestnut St, Philadelphia, Pennsylvania 19104, United States; ‡ Department of Chemistry, KTH Royal Institute of Technology, Teknikringen 42, Stockholm SE-10044, Sweden; § Department of Chemistry, 6558 Temple University, 1901 N. 13th St., Philadelphia, Pennsylvania 19122, United States

## Abstract

The reactivity of isocyanoalkynes—alkynes bearing a terminal isocyanide—is an unexplored frontier. The absence of any isocyanoalkyne-based reactions stems from their particularly challenging synthesis, a route originally developed to determine the possible presence of isocyanoacetylene in interstellar gas clouds. The difficult flash-vacuum, pyrolysis-condensation synthesis (240 → −196 °C), the low yield (<10%), and the rapid polymerization of simple isocyanoacetylenes likely dissuaded attempts to investigate the chemistry of these highly energetic molecules. Described here is a robust, scalable synthesis of substituted isocyanoalkynes bearing aromatic, heterocyclic, and aliphatic substituents, and a structural analysis complemented by computations that identify molecular hot spots. These analyses have been particularly important in developing several exploratory reactions that reveal the high reactivity of isocyanoalkynes in unique, multi-bond-forming cascades. The synthesis, structural analyses, and reactivity of isocyanoalkynes provides a robust foundation for exploring, what augurs to be, a versatile new family of nitrogen-substituted alkynes.

## Graphical abstract

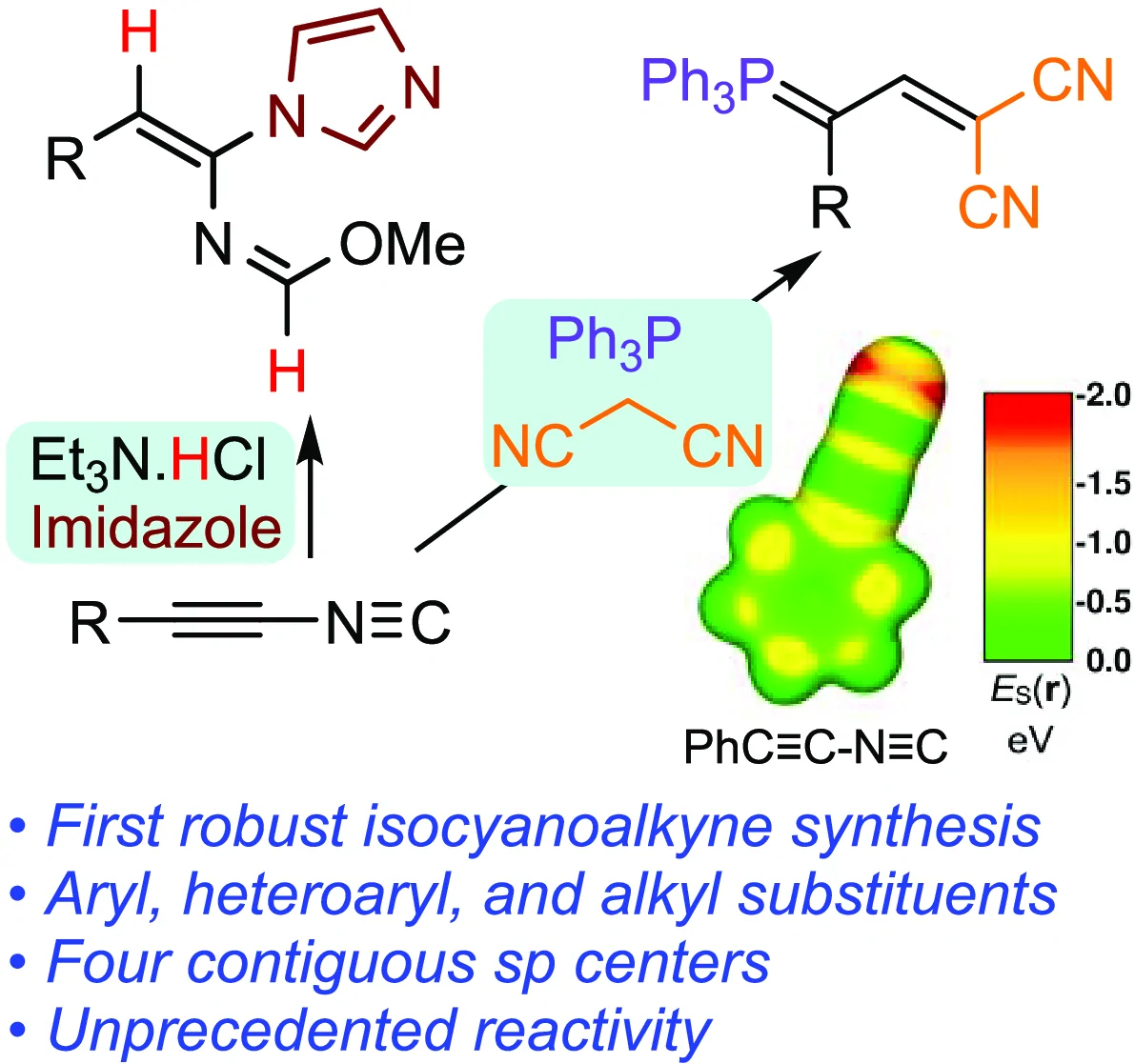

## Introduction

Interstellar molecules provide unique inspiration for new chemistry as the frigid vacuum in space can stabilize structures inaccessible under terrestrial conditions. Fullerene is a case in point. The serendipitous synthesis of the cage-like, carbon allotrope led to the detection of fullerene in the interstellar medium^1^ triggering a ream of important advances in fields ranging from electronics to nanoscience.^2^

The identification of simple isocyanides in the interstellar medium has sparked an analogous flurry of interest because these high-energy molecules are postulated as origin-of-life precursors.^3^ Speculation over the presence of more energetic sp-hybridized isocyanides in the interstellar medium^4^ led to the synthesis of isocyanoacetylene (HC≡C–N≡C, **1a**) via a flash vacuum pyrolysis (FVP)-demetallation of the chromium isocyanide complex **2** (Scheme 1A).^5^ The pioneering synthesis afforded a few milligrams of isocyanoacetylene (**1a**) whose key spectral signatures unambiguously matched those emitted from several interstellar gas clouds.^6^

**Scheme 1:**
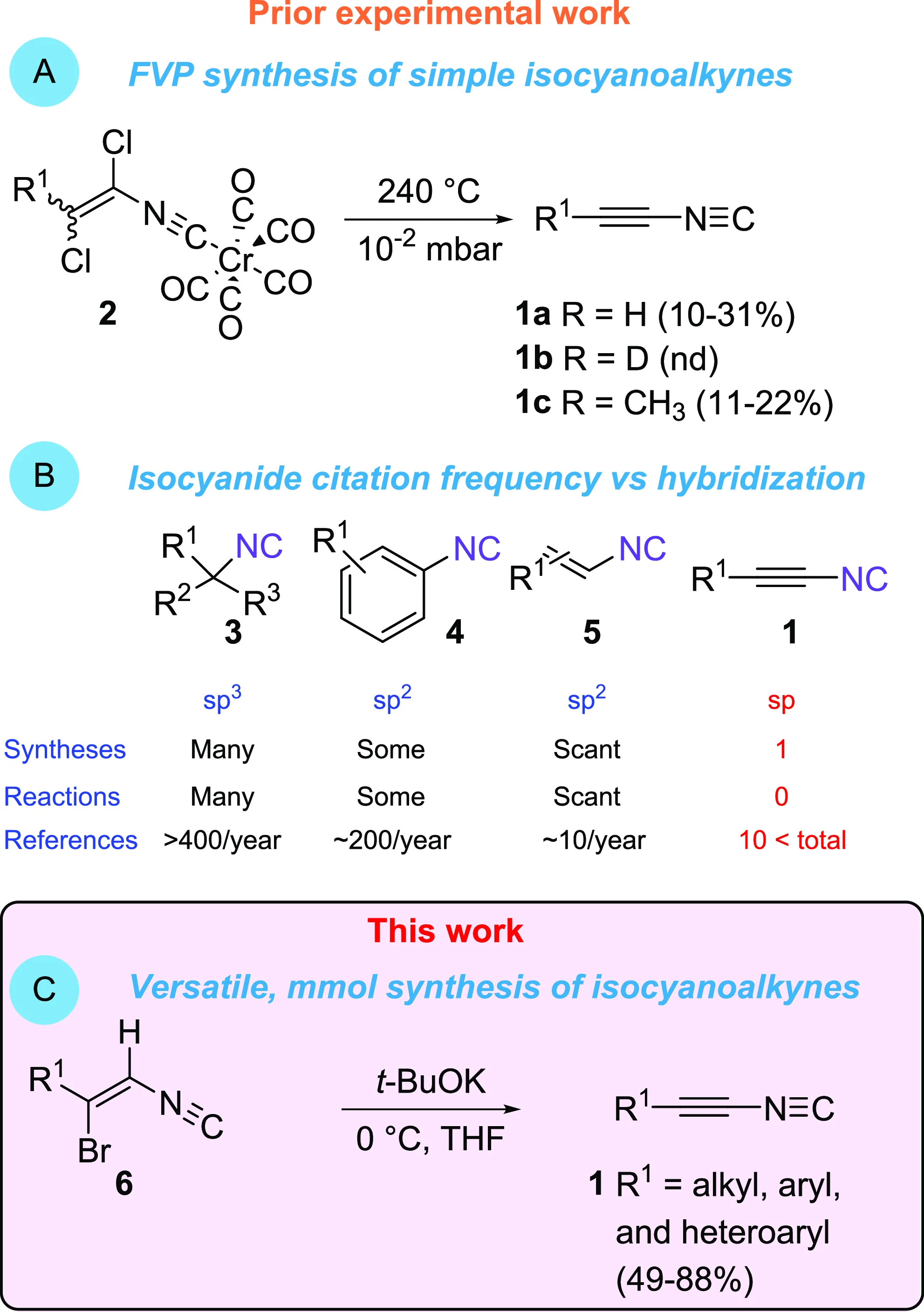
Isocyano*alkyne* vs Isocyano*alkane*, Isocyanoarene, and Isocyano*alkene* Chemistry

An absence of reactions with isocyanoacetylene (**1a**), or any simple derivatives, reflects their challenging synthesis and the rapid polymerization of these unstable gases above −80 °C.^7^ However, the instability hints at an untapped opportunity to harness the high reactivity embedded in the two contiguous triple bonds in new bond-forming reactions. The potential to discover new reactivity from isocyanoalkynes is high because most isocyanide-based reactions employ either isocyanoalkanes or isocyanoarenes (Scheme 1B, compare the frequencies of **3** and **4** with those of **5** and **1**);^8^ only a few reactions employ isocyanoalkenes,^9^ while virtually *nothing* is known about the experimental reactivity of isocyanoalkynes.

The important advance presented here is a reproducible, scalable synthesis of isocyanoalkynes which allows their unique reactivity to be explored. The approach is predicated on a rather simple dehydrohalogenation of bromoisocyanoalkenes (**6**, Scheme 1C), functionalities whose synthesis and reactions have only recently been developed.^10^ Described here is a robust synthesis of diverse isocyanoalkynes bearing aliphatic, aromatic, and heterocyclic substituents, a combined experimental and computational analysis of their structures, and exploratory forays to probe their reactivity.

## Results and Discussion

Access to isocyanoalkynes emanated from the naïve idea of dehydrohalogenating a suitable haloisocyanoalkene (Scheme 1C).^11^ As a class, haloisocyanoalkenes are themselves relatively rare: the first reported congeners were two α-chloroisocyanoalkenes isolated from soil bacteria^12^ whereas the first syntheses were of β-bromoisocyanoacrylates for an isocyanide-based approach to heterocycles.^13^ Only recently have β-bromoisocyanoalkenes **6** suitable for dehydrohalogenation, become readily accessible through a regioselective bromination-dehydration of the corresponding vinylformamides **7** (eq 1).^10^

1

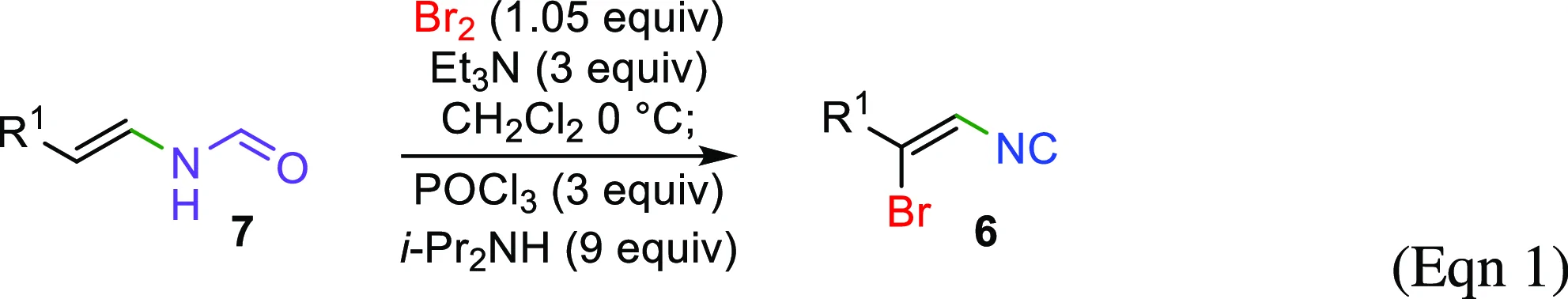




The ‘simple’ bromoisocyanoalkene dehydrohalogenation was initially probed with the phenyl-substituted congener **6a** (**6**, R^1^ = Ph) as a prototype (Scheme 2).^14^ The selection of **6a** was predicated on forming isocyanoalkyne **1d** with extended delocalization between the two π-systems to improve the stability, while the mass imparted by the phenyl substituent was anticipated to afford a more easily manipulated non-volatile oil or solid. Initial eliminations were promising but particularly challenging because of the instability of the resulting phenylisocyanoalkyne **1d**: concentrating or heating relatively clean samples from the elimination resulted in rapid decomposition to a black tar.^8^ By comparing crude reaction mixtures, the solvent, temperature, and base (LDA, KHMDS, Cs_2_CO_3_) were optimized, which identified *t*-BuOK in THF at 0 °C as ideal for cleanly affording **1d** (74% yield based on an NMR analysis of the crude reaction mixture with an internal standard). However, isolating pure samples of **1d** was frustrated by a strong tendency of neat samples to polymerize. Eventually, a robust workup and purification was developed using volatile ether-pentane mixtures to rapidly access concentrated samples for purification. Combining the optimized workup with chromatography on silanized “C-2 silica”^15^ was critical for minimizing polymerization and preventing irreversible adsorption onto the silica matrix. Using the optimized workup and chromatography protocol allowed a reliable synthesis of an array of isocyanoalkynes **1e**-**p**, all of which proved to be significantly more stable than **1d** (Scheme 2).^16^ Unwittingly, **1d** proved to be anything but prototypical.

**Scheme 2:**
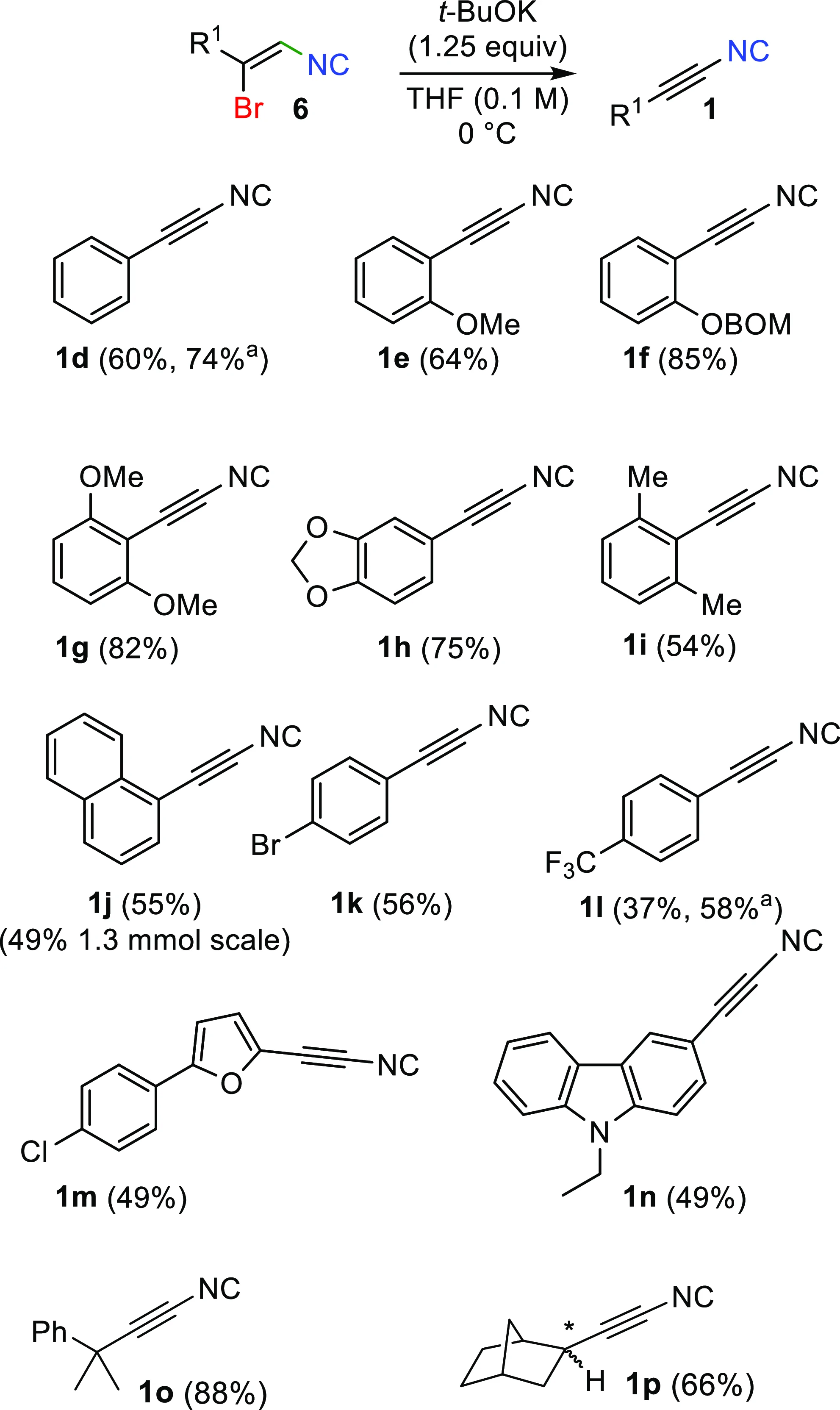
Synthesis of Diverse Isocyanoalkynes

a The yield was obtained from an internal standard added to crude reaction mixture because of degradation and absorption during chromatographic purification.

Initially, isocyanoalkynes were prepared with modest electronic changes in the aromatic ring; synthesizing arylisocyanoalkynes with electron-donating (**1e**-**1h**) or neutral substituents (**1i**-**1j**) was uneventful. The naphthyl isocyanoalkyne **1j**, prepared on a 1.3 mmol scale in 49% yield, was a particularly stable crystalline solid. Similarly, crystalline isocyanoalkynes **1g** and **1k** showed only trace degradation after storage at −20 °C for several months. Crystalline isocyanoalkynes and those with electron-rich substituents, seem to be more stable than those with more electron-deficient substituents, such as **1l** and **1m**, that were prone to polymerize at room temperature. Heterocyclic substituents at the sp carbon were tolerated (**1m**-**1n**), while the aliphatic sp^3^-hybridized substituents with quaternary or tertiary centers adjacent to the acetylenic carbon were among the more stable congeners (**1o**-**1p**, respectively).

Crystallographic analysis of **1j** provided insight into the isocyanoalkyne structure (Figure 1A). The carbon–nitrogen triple bond of the isocyanoalkyne **1j** is slightly elongated (1.17 Å) compared with the average isocyanide bond length (1.14 Å) while the C–NC single bond is shortened compared to the analogous bond in isocyano*alkanes*;^5,17^ the contraction of the C–NC bond in moving from sp^3^ to sp^2^ to sp hybridization, correlates with a progressive shortening caused by the increasing p character of the alkyl carbon.^5^ Comparing the structural characteristics of the isocyanoalkyne **1j** with the isoelectronic alkynenitrile **8a** is particularly revealing because the bond lengths are quite similar despite the two functionalities having very different spectral data.^18^

**Figure 1:**
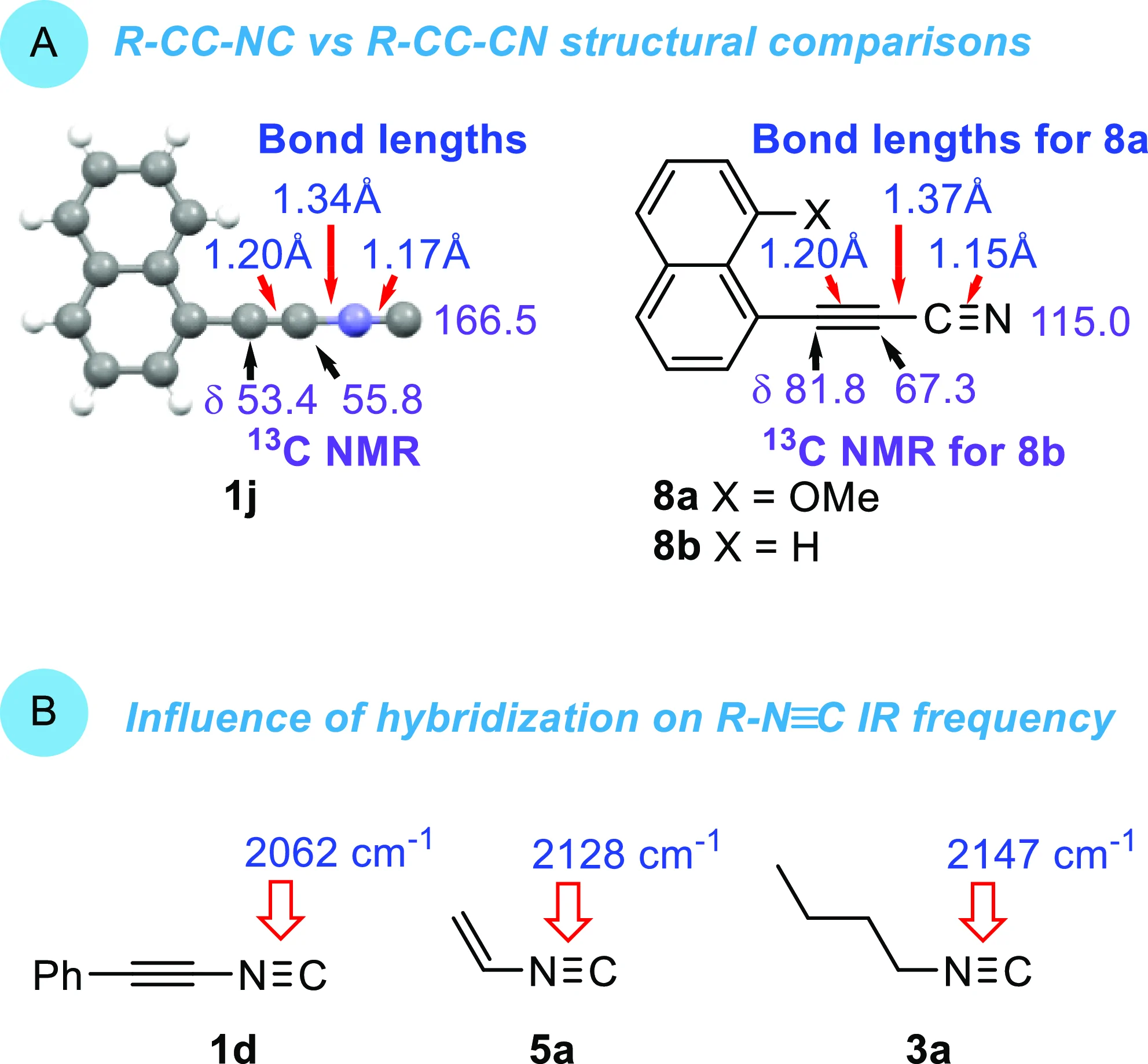
Structural features of isocyanoalkynes compared to related congeners.

Insight into the interplay of the isocyanide and alkyne π-systems was gleaned by comparing the relative ^13^C NMR shift differences of the acetylenic carbons of isocyanoalkyne **1j** with those of the alkynenitriles **8** (see values listed in Figure 1A). The ^13^C NMR shift difference between the β and α alkyne carbons, Δδ (β – α), for **1j** is −2.4 with the α-carbon being furthest downfield (55.8 ppm vs 53.4 ppm for the α- and β-carbons, respectively). Typically, electron-withdrawing substituents on alkynes induce downfield shifts for the β-carbon as observed for the acetylenic nitrile **8b** ^19^ for which Δδ = +14.5 (81.8 and 67.3 ppm for the β- and α-carbons, respectively); the relative ^13^C NMR chemical shift values of the acetylenic carbons in isocyanoalkynes are closer to electron-rich ynamides^20^ and ynamines^21^ than acetylenic nitriles. The upfield ^13^C NMR chemical shifts for the alkyne signals in **1j** suggests that the isocyanide nitrogen has shifted some electron density from the terminal isocyanide carbon into the alkyne π* orbital relative to aliphatic isocyanides, though overall the alkyne remains strongly polarized by the powerful electron-withdrawing effect of the isocyanide.^22^

The movement of electron density from the isocyanide nitrogen into the alkyne π* correlates with the increasing IR frequencies within the series of sp, sp^2^, and sp^3^-hybridized isocyanides (Figure 1B). The low IR stretching frequency for **1d** is typical of the isocyanoalkynes **1** relative to isocyanoethylene (**5a**) and isocyanobutane (**3a**, Figure 1B). The IR frequency trends are likewise consistent with some weakening of the triple bond caused by delocalization of the nitrogen lone pair into the acetylenic π* orbital.^23^

Computational analyses complement the electronic profile provided by crystallographic and spectral data. After scaling by 0.9642 to compensate for anharmonic effects, the B3LYP/6-31+G­(d,p) IR frequencies of the nitrogen–carbon triple bond of the isocyanides are in very good agreement with the experimental frequencies, showing computed N≡C bond lengths decreasing with increasing IR frequency [i.e., in the order **1d** (1.184 Å) > **5a** (1.180 Å) > **3a** (1.174 Å)].

Comparing the computed surface electrostatic potentials (*V*_*S*_(**r**)) of the isocyanoalkyne **1d** with 3-phenylpropiolonitrile (**8c**) indicates that the isocyano group has an only slightly weaker electron-withdrawing capacity than the nitrile (Figure 2), consistent with the reported Hammett σ_p_ values of 0.48 and 0.56 for the isocyanide and nitrile, respectively.^24^ The local *V*_*S*_(**r**) minima (*V*_S,min_) over the carbon–carbon triple bonds of **1d** and **8c** are 0.0 and −2.1 kcal/mol, respectively; compared to methylphenylacetylene (**9a**), which contains a weakly donating methyl group, the *V*_S,min_ is −24.7 kcal/mol which reflects the strong influence of the electron-withdrawing isocyanide and nitrile groups for the respective alkynes, **1d** and **8c**. Over the methyl group of methylphenylacetylene (**9a**), *V*_*S*_(**r**) is positive whereas at the isocyano and cyano lone pairs of **1d** and **8c** they are strongly negative; the *V*_*S*,min_ values are −48.0 and −55.7 kcal/mol, respectively. For comparison, the corresponding *V*_*S*,min_ values of isocyanoethylene **5a** and isocyanobutane **3a** are −52.5 and −57.8 kcal/mol, respectively, consistent with an electron donation from the butyl group to the isocyano group; in comparison, the phenylacetylene has a relatively weak electron-withdrawing effect on the isocyano group in phenylisocyanoalkyne (**1d**). The observed variations in *V*_*S*_(**r**) are consistent with the reported Hammett σ_p_ values for the substituents, −CN (0.56), -NC (0.48), −CCH (0.22), CH_3_ (−0.17) and -C_4_H_9_ (−0.19).^22^

**Figure 2:**
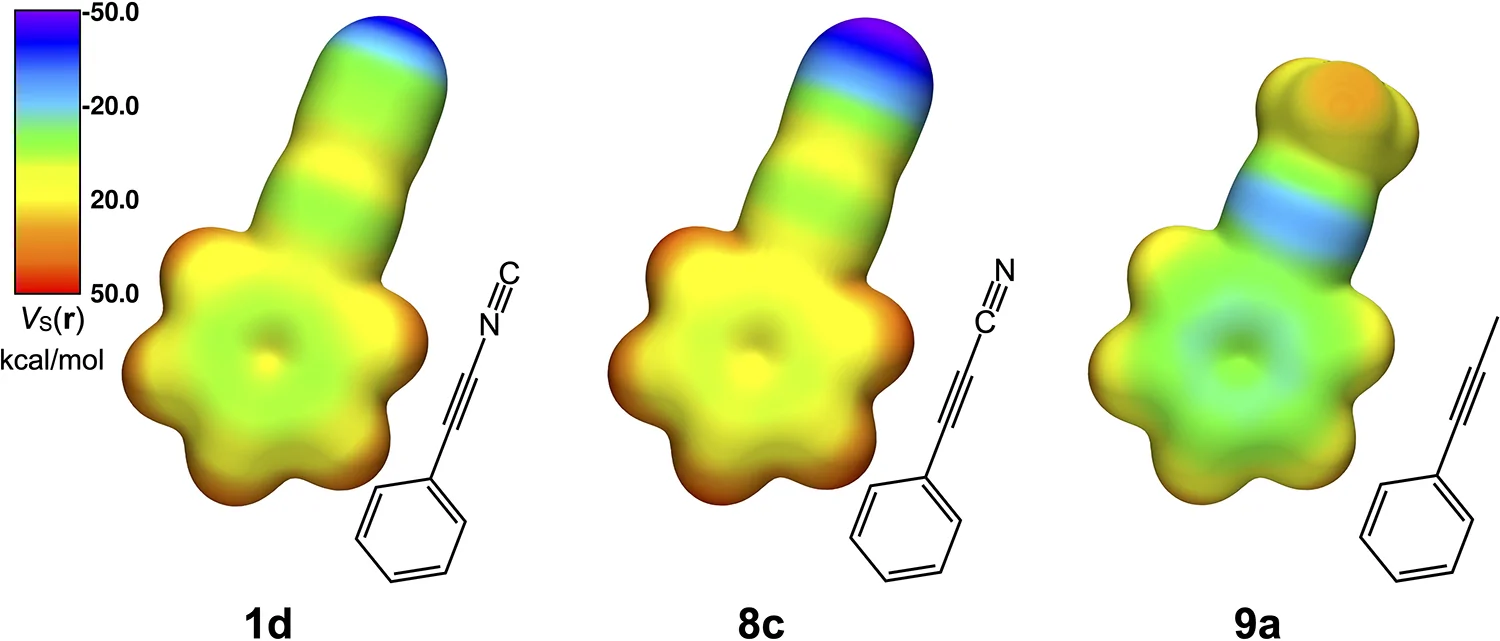
Comparison of the surface electrostatic potential *V*_*S*_(**r**) of isocyanoalkyne **1d** with nitrile and methyl congeners.

The local electron attachment energy (*E*_*S*_(**r**)),^25^ an electrophilicity index derived from unoccupied orbitals with negative energies, was calculated to provide insight into the reactive sites of isocyanoalkynes (Figure 3). The *E*_*S*_(**r**) for phenylisocyanoalkyne (**1d**) shows the terminal isocyanide carbon to be particularly electrophilic with an *E*_*S*,min_ of −2.06 eV, while the acetylenic β-carbon is less activated with an *E*_*S*,min_ of −1.02 eV (Figure 3); the effect is even more pronounced in the *E*_*S*_(**r**) map of the *p*-trifluoromethylphenylisocyanoalkyne **1l**, potentially explaining the instability of **1l** as the result of nucleophilic attack on the isocyanide carbon. Comparing the *E*_*S*_(**r**) maps of **1d** and **1l** with that of 3-phenylpropiolonitrile (**8c**) shows that nitrile **8c** has a lower *E*_*S*,min_ (−1.30 eV) at the β-carbon, which correlates well with the facile conjugate addition of nucleophiles to the β-carbon of alkynenitriles.^26^

**Figure 3:**
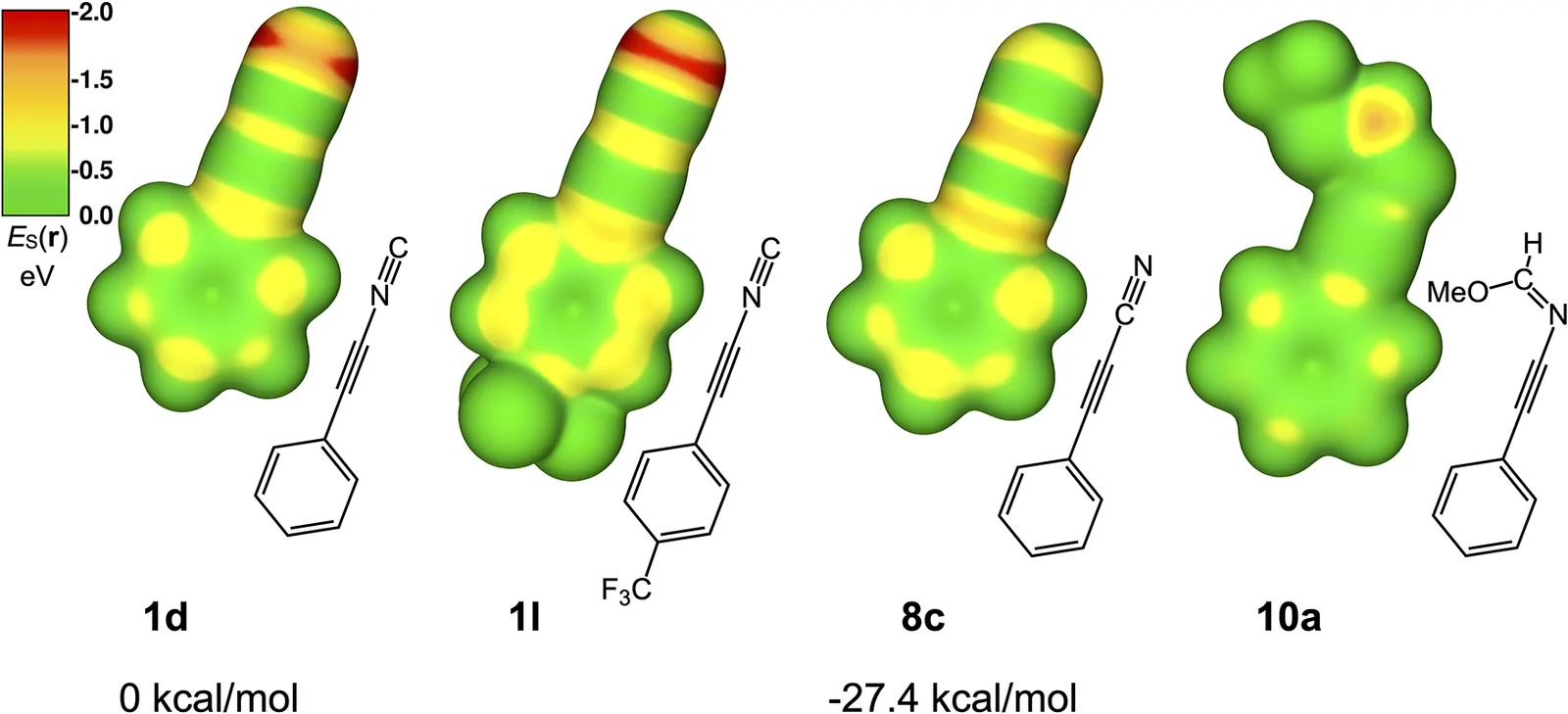
Comparison of the local electron attachment energy of phenyl-substituted isocyanoalkynes with related congeners. Relative energies of **1d** and **8c** have been computed at the CBS-QB3 level.

A comparison of the total enthalpy (CBS-QB3 level) of the isocyanoalkyne **1d** and the acetylenic nitrile **8c** indicates that the isocyanide is 27.4 kcal/mol less stable than the corresponding nitrile. In contrast, the enthalpy difference between methylisocyanide and acetonitrile is 24.3 kcal/mol, consistent with the phenylalkyne destabilizing the isocyanide compared to the corresponding nitrile, by around 3 kcal/mol. The high relative enthalpy of the isocyanoalkynes is consistent with the observed instability of some substrates and provides a strong thermodynamic driving force in synthetic reactions (vide infra).

The combined electron-withdrawing effects of the phenyl and isocyanide groups increases the electrophilicity of the alkyne, activating the β-carbon for attack by nucleophiles, but less so than for alkynenitriles. The electron-rich alkynylimidate **10a** is on the other extreme with a nucleophilic triple bond as indicated by the values of their associated minima in the surface average local ionization energy (*I*_*S*,min_), which are 9.07, 10.26, and 10.57 eV, respectively.^27^

Preliminary forays to probe the reactivity of isocyanoalkynes initially evaluated their reactions with nucleophiles for which the analogous reactions with alkynenitriles afford conjugate addition products^28^ (Scheme 3). Exposing isocyanoalkyne **1j** to imidazole and Et_3_N.HCl in methanol did not afford a conjugate adduct but efficiently provided the doubly nitrogen-substituted alkene **13a** (Scheme 3a). Presumably the initial acid-promoted isocyanide insertion into methanol to afford the rare alkynylimidate **11a** is followed by protonation of the electron-rich alkyne to afford **12a** that was intercepted by imidazole to afford **13a** (**11a** is anticipated to have the same electronic profile as **10a**, Figure 3); consistent with this mechanistic proposal was the isolation of **11a** in 46% yield when the reaction was performed in the absence of imidazole. Switching the nucleophile to *p*-bromothiophenol generated an analogous double addition product **15a** whose structure was secured by crystallographic analysis (Scheme 3B). Formation of **15a** is consistent with an analogous acid-promoted insertion of isocyanoalkyne **1j** into the thiol to afford **14a** whose protonation to form a keteniminium is followed by addition of a second equivalent of thiol (cf. **11a** → **12a** → **13a**) to afford **15a**.

**Scheme 3:**
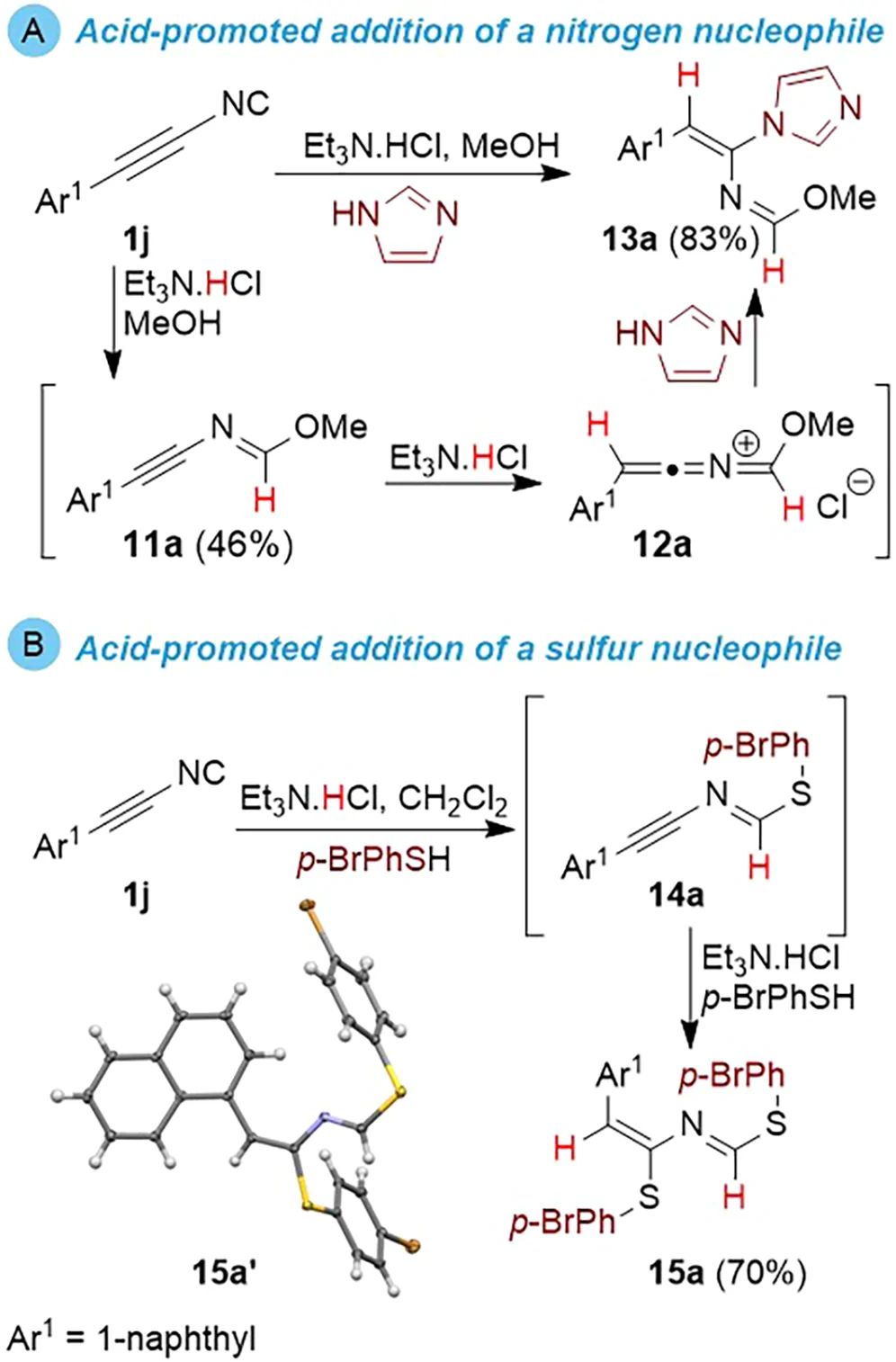
Acid-Promoted Isocyanoalkyne Cascades

The reactions of isocyanoalkynes with nucleophiles under basic conditions differ significantly from those of the corresponding alkynenitriles.^28^ With sodium methoxide in methanol, the electrophilic isocyanoalkyne **1j** incorporated three equivalents of MeOH to afford the acetal **18a** (Scheme 4A). The reaction is a rare example of two alkyoxy additions to an isocyanide.^29^ Presumably methoxide addition to the terminal isocyanide is followed by protonation to afford **11a** ^30^ that added a second equivalent of methoxide to afford **16a**; subsequent protonation followed by methoxide addition to the ketenimine functionality of **17a** would, after protonation, afford **18a**.

**Scheme 4:**
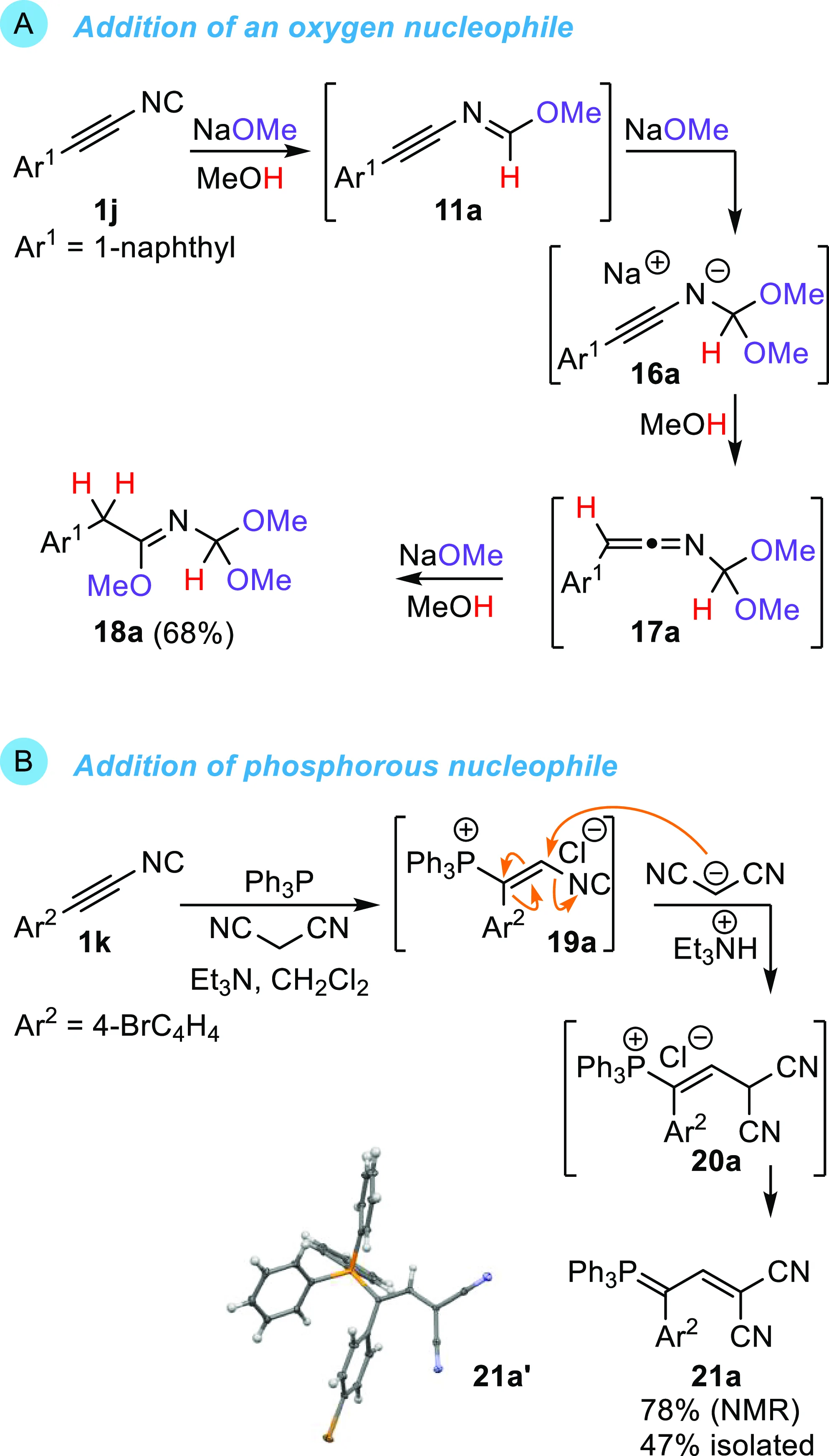
Base-Promoted Isocyanoalkyne Cascades

Under weakly basic conditions with the isocyanoalkyne **1k**, Ph_3_P, malononitrile, and Et_3_N, (Scheme 4B) a clean, deep-seated rearrangement occurred to afford the structurally reorganized ylide **21a** whose constitution was secured by X-ray crystallography (Scheme 4B). Attachment of the phosphonium salt at the benzylic position in **21a** suggests an initial conjugate addition of Ph_3_P to the β-acetylenic carbon of **1k** consistent with the computed electrophilicity of the alkyne. Subsequent protonation of the intermediate vinyl anion by malononitrile would afford **19a** and malononitrile anion.^31^ The resulting vinylphosphonium salt **19a** would polarize the alkene, favoring addition of the malononitrile anion at the isocyanide-bearing carbon to then trigger an ejection of cyanide (**19a** → **20a**); similar addition-eliminations of vinylphosphoniums are known,^32^ although this is the first report of cyanide ejection by cleavage of an isocyanide.^33^ Subsequent deprotonation of **20a** efficiently generated the ylid **21a** (Scheme 4B), though purification by silica gel chromatography caused significant mass loss through adsorption as is common with ylids.^34^

## Conclusions

A diverse array of isocyanoalkynes have been synthesized via a robust, base-induced dehydrohalogenation of bromoisocyanoalkenes. The strategy addresses the prior difficulty of synthesizing isocyanoalkynes, allowing the first mmol synthesis of diversely substituted isocyanides and investigation of the reactivity profile for this essentially new functionality. Initial reactions of isocyanoalkynes reveal a propensity for complex bond-forming cascades with nucleophiles in acidic or basic media, a reactivity that is distinctly different from most alkynes with electron-withdrawing substituents. Access to isocyanoalkynes adds an unexplored functionality to the arsenal of nitrogen-substituted alkynes whose unique multi-bond-forming cascades are primed for development.

## Supplementary Material



## Notes

Experimental procedures, characterization data, NMR spectra, computational analyses, and crystallographic data (PDF)
