# Supplementary material for: Isocyanoalkynes: Leveraging a High-Energy Interstellar Lead for Unique Multi-Bond-Forming Cascades
Source: J Am Chem Soc. 2026 Sep 23;148(39):42305–11. doi: 10.1021/jacs.6c12613 (PMC13651522; doi:10.1021/jacs.6c12613)
Supplement: Supplementary file 1 [file ja6c12613_si_001.pdf]

Isocyanoalkynes: Leveraging A High-Energy Interstellar Lead for Unique Multi-bond-forming Cascades

Supporting Information

Huan Tian, Tore Brinck*, Christopher Absil, and Fraser F. Fleming*

Department of Chemistry, Drexel University, 32 South, 32nd St., Philadelphia, PA 19104

Compound	Procedure	^{1}H NMR	^{13}CNMR
General Experimental Procedures	S6		
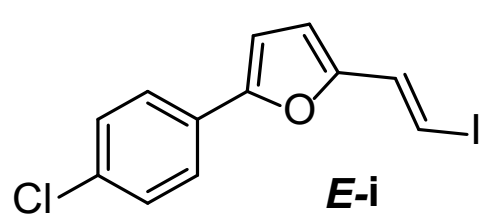 ***E*-i** 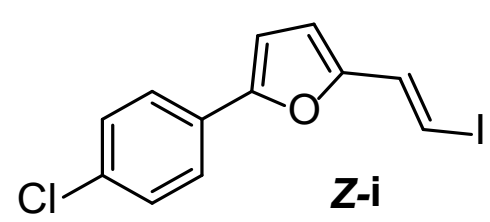 ***Z*-i**	S7	S22	S22
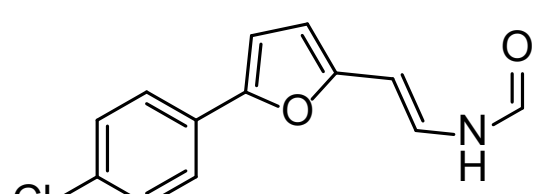 ***E*-7m**	S8	S23	S23
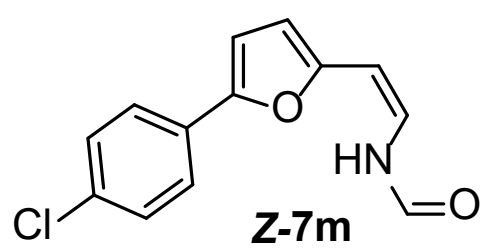 ***Z*-7m**	S8	S24	S24
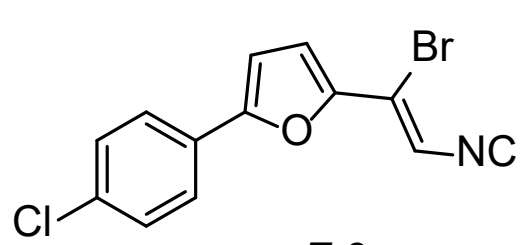 ***Z*-6m**	S9	S25	S25
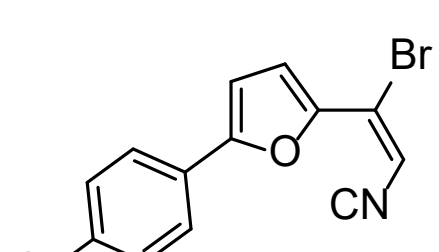 ***E*-6m**	S9	S26	S26
General Isocyanoalkyne Synthesis	S10		

Figure S1. Images showing the effect of concentrating solutions of **1d** S11

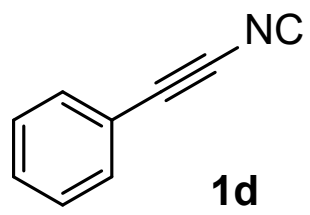 **1d**	S12	S27	S27
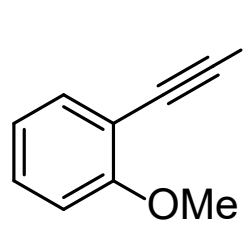 **1e**	S12	S28	S28
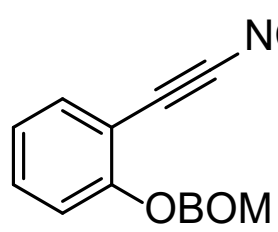 **1f**	S13	S29	S29
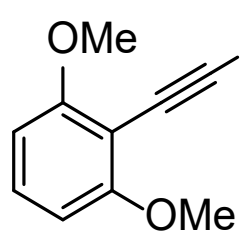 **1g**	S13	S30	S30
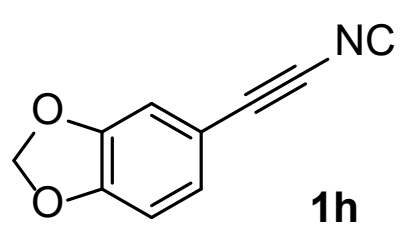 **1h**	S14	S31	S31
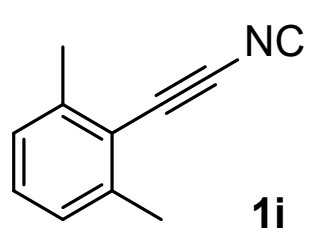 **1i**	S14	S32	S32
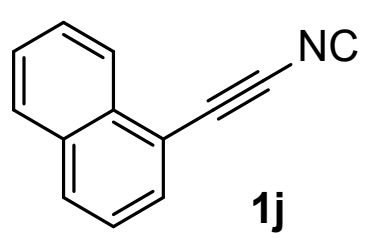 **1j**	S15	S33	S33
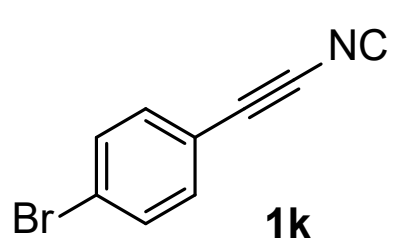 **1k**	S15	S34	S34
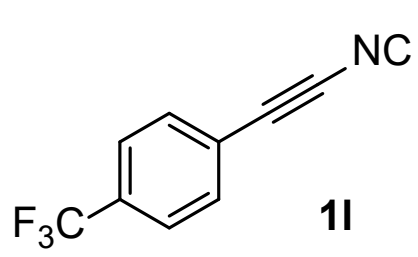 **1l**	S16	S35	S35
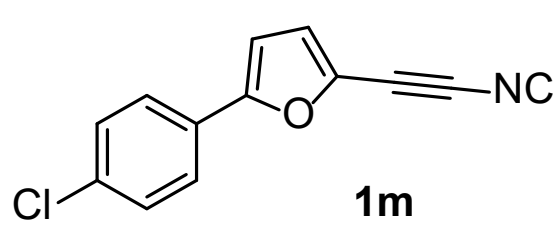 **1m**	S16	S36	S36

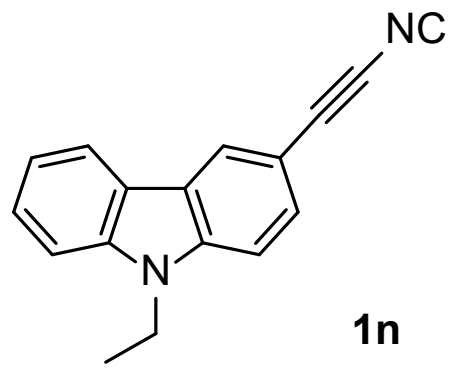 **1n**	S17	S37	S37
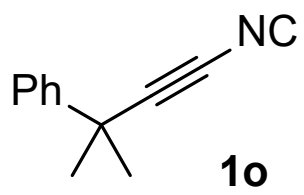 **1o**	S17	S38	S38
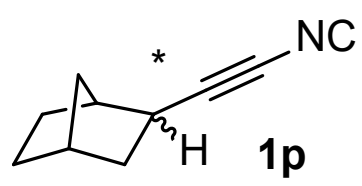 **1p**	S18	S39	S39
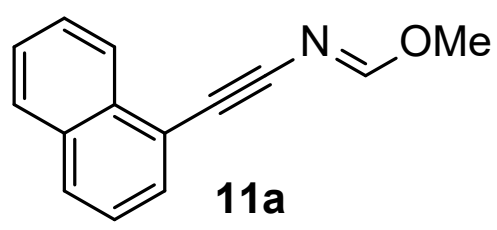 **11a**	S18	S40	S40
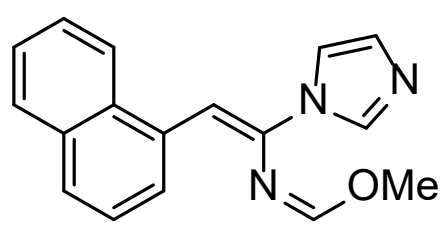 **13a**	S19	S41	S41
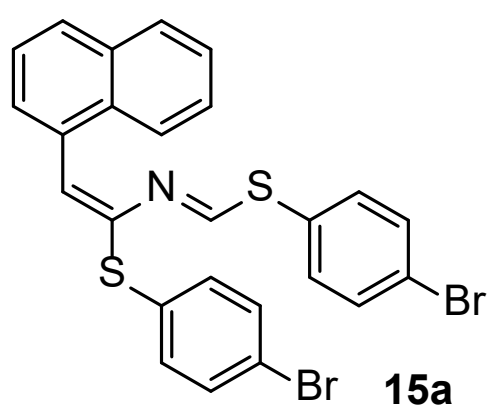 **15a**	S19	S42	S42
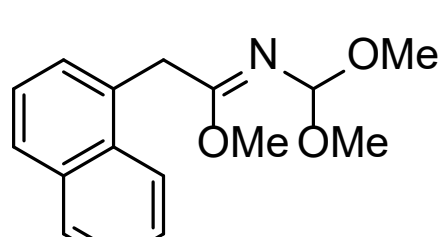 **18a**	S20	S43	S43
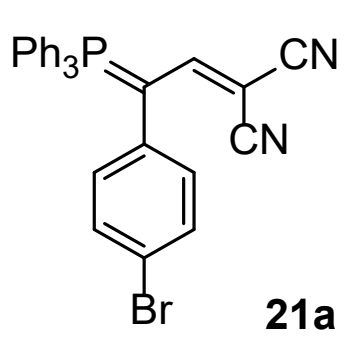 **21a**	S20	S44	S44

$^{31}P\{^{1}H\}$ NMR for **21a** S45

Figures S1, S2, and S3: Crystal structure plots for **1j**, **15a**, **21a** S46

Table S2. Citations of the Top 10 Isocyano*alkanes* S47

Figure S4: Citations of the Top 10 Isocyano*alkanes* over Time S48

Table S3. Citations of the Top 10 Isocyano*alkenes* S49

Figure S5: Citations of the Top 10 Isocyano*alkenes* over Time S50

Table S4. Citations of the Top 10 Isocyano*arenes* S51

Figure S6: Citations of the Top 10 Isocyano*arenes* over Time S52

Theoretical Background S53

Computational procedures S54

References S61

The isocyanoalkyne ^{13}C NMR spectra contained weak signals for the sp isocyanide and acetylenic carbons; the acetylenic carbon was often unable to be detected which is likely due to a combination of an absence of NOE enhancement and quadrupolar coupling by the adjacent nitrogen. The standard relaxation time between pulses was increased from 2s to 60 s which allowed detection of several R-C≡CN≡C carbons. The chemical shifts of the R-C≡CN≡C unit in Table S1 is the largest such collection which should prove useful as others prepare and characterize this functionality; comparable chemical shifts of previously isolated isocyanoalkynes are provided at the end of the table.

Table S1. Diagnostic ^{13}C NMR Data for Isocyanoalkynes in $CDCl_3$

Isocyanoalkyne	R-C≡CN≡C	R-C≡CN≡C	R-C≡CN≡C
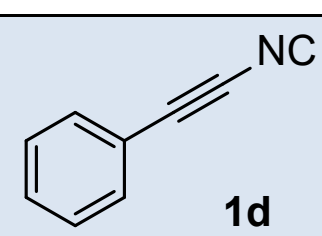 **1d**	165.4	-	57.1
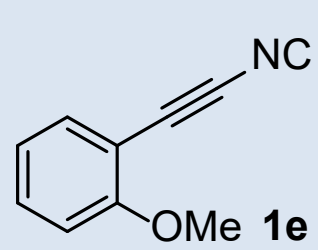 **1e**	165.4	-	54.1

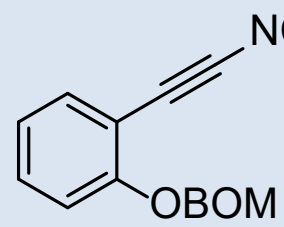 1f	165.6	70.5 (t)	54.1
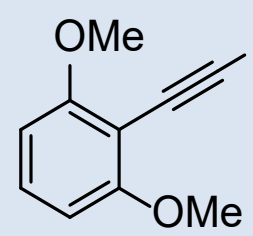 1g	165.5	55.6	50.9
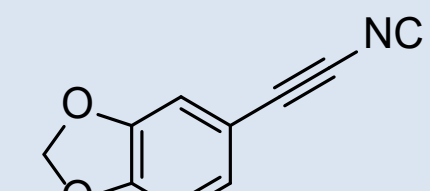 1h	165.0	101.8 (t)	57.3
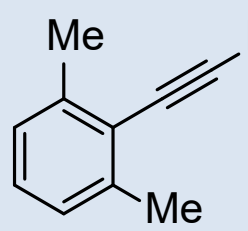 1i	166.3	-	55.2
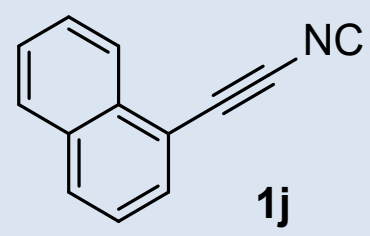 1j	166.5	55.8[a]	53.4
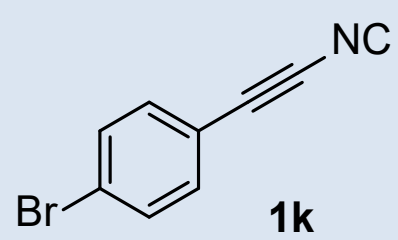 1k	166.4	-	57.1
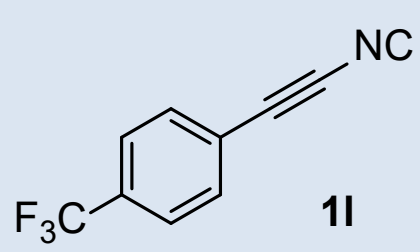 1l	167.0[b]	70.0[b]	56.4[b]
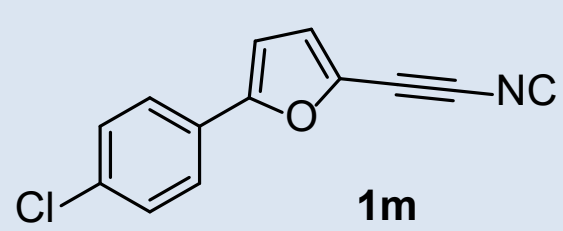 1m	171.2[b]	77.2[b]	49.0[b]
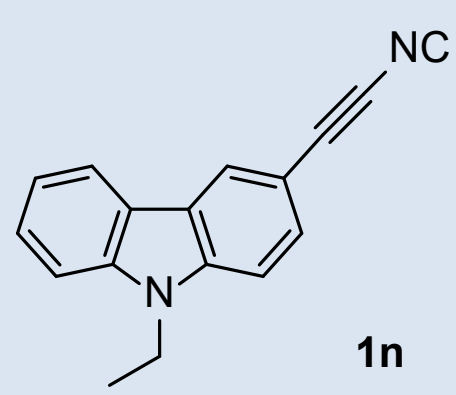 1n	164.1	-	59.1
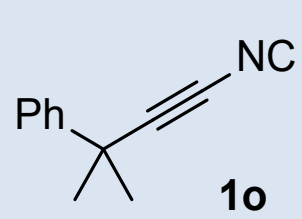 1o	160.5	64.9	62.2
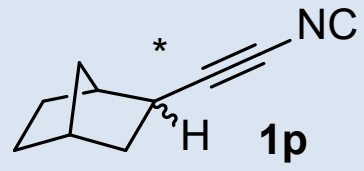 1p	158.6 (157.9)		63.7 (62.8)

[a] 55.7 in DMSO-*d6*. [b] In DMSO-*d6*

Previously Reported Characterization of Known Isocyanoalkynes

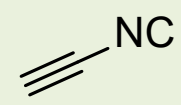 **1q**	160.2	61.6	47.9[1]
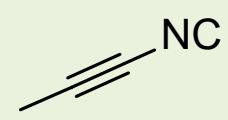 **1r**	155.2	59.1	55.6[1]

General Experimental Procedures. All nonaqueous reactions were performed in oven- or flame-dried glassware under a nitrogen atmosphere. All chemicals were purchased from commercial vendors and used as received unless otherwise specified. Anhydrous tetrahydrofuran (THF) and dioxane were distilled from benzophenone-sodium under N_2 before use; dichloromethane and diisopropylamine (*i*-PrNEt) were obtained from a solvent purification system (Innovative Technology Inc., model PS-MD7). Reactions were magnetically stirred and monitored by thin layer chromatography (TLC) plates precoated with 250 μm silica gel. The purification of materials sensitive to silica gel were purified on C-2 silica prepared in-house as described previously;[2] the columns were washed with a Et_3N-pentane solution (1:9 ratio, 20 mL) and then equilibrated with pentane before use. Reactions requiring heating were performed on a hot-plate stirrer using an oil bath. ^1H NMR and ^{13}C NMR high resolution nuclear magnetic resonance spectra were recorded on a Varian Inova 400 (400 MHz/101 MHz) instrument. Chemical shifts are reported relative to TMS (δ 0.00) or the residual $CHCl_3$ signal (δ 7.26) for ^1H NMR and chloroform (δ 77.16) for ^{13}C NMR. IR spectra were recorded as thin films (PerkinElmer Spectrum 100 FT-IR Spectrometer). High-resolution mass spectra were obtained on a Bruker 12.0 Tesla APEX – Qe FTICR-MS with an Apollo II ion source (positive electrospray ionization) and a Thermo-Finnigan LTQ-FT 7T FT-ICR spectrometer with an atmospheric pressure chemical ionization (APCI)

source with direct infusion run in positive ion mode at 5 kV.

General Precaution: No toxicity studies of the isocyanoalkynes have been performed so the same care should be followed in working with these chemicals as with any research material of unknown toxicity. Many naturally occurring isocyanides have antibacterial and antifungal properties and some are being evaluated as drug candidates. [3] While low molecular weight isocyanides sometimes have an unpleasant odor, those with a high steric demand around the isocyanide and those with a high molecular weight generally have only a modest odor; in some cases the smell is quite pleasant. [4] Most of the isocyanoalkynes described here have a modest odor which, in the worst case, is less than that of low molecular weight isocyanides.

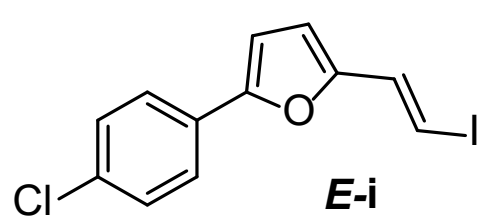

***E*-i**

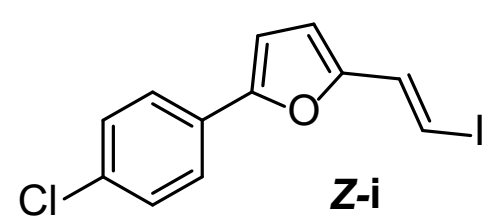

***Z*-i**

(E)-2-(4-Chlorophenyl)-5-(2-iodovinyl)furan ***(i)***. Following the published coupling procedure[6] with 5-(4-chlorophenyl)furan-2-carbaldehyde [5] (300 mg, 1.45 mmol), $CrCl_2$ (713.7 mg, 5.81 mmol), and iodoform (743.2 mg, 1.89 mmol) for 2 h, with purification by MPLC (12-g cartridge silica gel, 100% Hexanes), furnished 286 mg (59 %) of a 3:1 mixture of *E*- and *Z*-2-(4-chlorophenyl)-5-(2-iodovinyl)furan as a yellow solid: mp = 70.0 - 73.9 °C; IR 3122, 3064, 1700 cm^{-1}; HRMS (+ESI) m/z [M^+] Calcd. for $C_{12}H_8ClIO$ 329.9308, found 329.9321. For (*E*)-2-(4-chlorophenyl)-5-(2-iodovinyl)furan (***E*-i**): ^{1}H NMR (400 MHz, $CDCl_3$) δ 7.57 (d, *J* = 8.6 Hz, 2H), 7.34 (d, *J* = 8.6 Hz, 2H), 7.18 (d, *J* = 14.8 Hz, 1H), 6.82 (d, *J* = 14.8 Hz, 1H), 6.59 (d, *J* = 3.5 Hz, 1H), 6.31 (d, *J* = 3.5 Hz, 1H); ^{13}C{1H} NMR (101 MHz, $CDCl_3$) δ 153.0, 152.5, 133.41, 132.4, 128.96, 128.7, 125.1, 110.8, 107.2, 74.7. For (*Z*)-2-(4-chlorophenyl)-5-(2-iodovinyl)furan ^{1}H NMR (400 MHz, $CDCl_3$) δ 7.67 (d, *J* = 8.6 Hz, 2H), 7.41 – 7.32 (m, 3H), 7.05 (d, *J* = 3.6 Hz, 1H), 6.71 (d, *J* = 3.6 Hz, 1H),

6.47 (d, *J* = 9.1 Hz, 1H); ^{13}C{1H} NMR (101 MHz, $CDCl_3$) δ 152.6, 151.2, 133.43, 128.98, 127.3, 125.2, 113.8, 110.77, 107.0, 74.9.

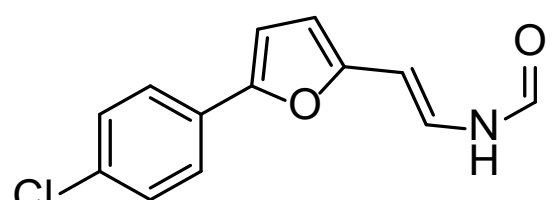

***E*-7m**

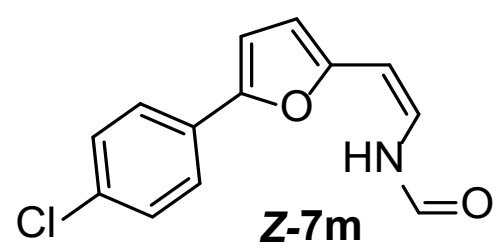

***Z*-7m**

E- and Z-N-(2-(5-(4-Chlorophenyl)furan-2-yl)vinyl)formamide ***(E-7m** *and* **Z-7m)**. Following the published coupling procedure [6] with a 3 :1 *E:Z*-mixture of 2-(4-chlorophenyl)-5-(2-iodovinyl)furan (**i**, 150 mg, 0.453 mmol,), formamide (0.36 mL, 9.08 mmol), CuI (8.7 mg, 46 μmol), Cs_2CO_3 (221.8 mg, 0.68 mmol) and *N,N'*-dimethylethane-1,2-diamine (14 μL, 91 μmol), for 1 h and purification by MPLC (12 g cartridge filled with C-2 silica[2] eluting with EtOAc/Hexanes 25:75 to 40:60) furnished 77.4 mg (69%) of the vinyl formamides **7m** as a 2:1 *E:Z* ratio of geometric isomers: IR (ATR) 3268, 2920, 2850, 1650 cm^{-1}; HRMS (+ESI) m/z [M+] Calcd. for $C_{13}H_{10}ClNO_2$ 247.0400, found 247.0406. For (*E*)-*N*-(2-(5-(4-chlorophenyl)furan-2-yl)vinyl)formamide (***E-7m***) isolated as a yellow solid: mp = 139.5 – 140.7 °C. The ^{1}H NMR spectrum (400 MHz, $CDCl_3$) showed two rotamers in a 1:1.9 ratio. For the major rotamer: ^{1}H NMR (400 MHz, $CDCl_3$) δ 8.26-8.19 (m, 1H), 8.10-7.98 (m, 1H), 7.60 – 7.55 (m, 3H), 7.35 – 7.29 (m, 2H), 6.59 (d, *J* = 3.4 Hz, 1H), 6.22 (d, *J* = 3.4 Hz, 1H), 6.11 (d, *J* = 14.5 Hz, 1H); ^{13}C{1H} NMR (101 MHz, $CDCl_3$) δ 158.0, 151.9, 150.8, 132.88, 128.92, 128.87, 124.8, 119.8, 109.5, 107.4, 103.6. For the minor rotamer: ^{1}H NMR (400 MHz, $CDCl_3$) δ 8.82 (br s, 1H), 8.43 (d, *J* = 11.0 Hz, 1H), 7.60 – 7.48 (m, 2H), 7.36 – 7.29 (m, 2H), 7.10 (dd, *J* = 14.0, 11.0 Hz, 1H), 6.61 – 6.59 (m, 1H), 6.19 (d, *J* = 3.4 Hz, 1H), 5.99 (d, *J* = 14.0 Hz, 1H); ^{13}C{1H} NMR (101 MHz, CDCl3) δ 162.7, 151.6, 150.6, 132.89, 128.96, 128.90, 124.7, 122.2, 109.2, 107.5, 102.4. For *(Z)-N*-(2-(5-(4- chlorophenyl)furan-2-yl)vinyl)formamide (***Z-7m***) isolated as a red oil, the ^{1}H NMR spectrum (400 MHz, CDCl3) shows two rotamers in a 1:1.9 ratio. For the major rotamer: ^{1}H NMR (400

MHz, $CDCl_3$) δ 8.73 (br. d, *J* = 11.5 Hz, 1H), 8.37 (s, 1H), 7.54 – 7.50 (m, 2H), 7.41 – 7.36 (m, 2H), 6.97 (dd, *J* = 11.5, 9.5 Hz, 1H), 6.7-6.6 (m, 1H), 6.32 (d, *J* = 3.5 Hz, 1H), 5.60 (d, *J* = 9.5 Hz, 1H); $^{13}C\{1H\}$ NMR (101 MHz, $CDCl_3$) δ 158.0, 152.3, 152.1, 133.41, 129.17, 128.6, 124.70, 117.8, 111.1, 107.7, 99.2. For the minor rotamer: ^1H NMR (400 MHz, $CDCl_3$) δ 8.69 – 8.60 (m, 1H), 8.42 (d, *J* = 11.3 Hz, 1H), 7.56 – 7.52 (m, 2H), 7.38 – 7.35 (m, 2H), 6.67 – 6.65 (m, 1H), 6.43 (dd, *J* = 11.3, 9.3 Hz, 1H), 6.29 (d, *J* = 3.6 Hz, 1H), 5.50 (d, *J* = 9.3 Hz, 1H); $^{13}C\{1H\}$ NMR (101 MHz, $CDCl_3$) δ 162.4, 152.4, 151.6, 133.38, 129.19, 128.3, 124.73, 120.5, 110.7, 107.5, 98.1.

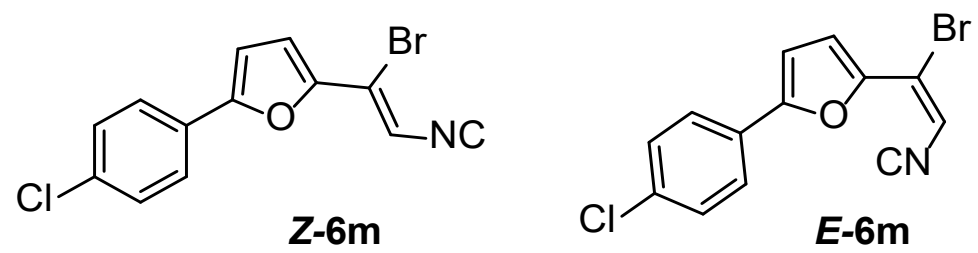

***Z*-6m** ***E*-6m**

*(Z)- and (E)-2-(1-Bromo-2-isocyanovinyl)-5-(4-chlorophenyl)furan (**Z-6m** and **E-6m**).* Following the published bromination-dehydration procedure for isocyanoalkenes [7] with (*E*)-N-(2-(5-(4-chlorophenyl)furan-2-yl)vinyl)formamide (***E*-7**, 48 mg, 0.33 mmol), bromine (11 µL 0.21 mmol), triethylamine (81 µL, 0.58 mmol), $POCl_3$ (54 µL, 0.58 mmol) and *i*-Pr_2NEt (0.3 mL, 1.74 mmol), for 2 h, and purification on C-2 silica[2] (1 cm x 2 cm column with 100% hexanes as the eluant), furnished 30.4 mg (51%) of the bromoisocyanoalkenes ***Z-6a*** and ***E-6a*** as a 3.3:1 ratio of diastereomers as a white solid that polymerized upon heating: IR 3063, 2922, 2115, 2086 cm^{-1}; HRMS (+EI) m/z [M^+] Calcd. for $C_{13}H_7BrClNO$ 306.9400, found 306.9408. Repeated purification by column chromatography on C-2 silica gel afforded pure samples of each diastereomer. For (*Z*)-2-(1-bromo-2-isocyanovinyl)-5-(4-chlorophenyl)furan (***Z-6a***): ^1H NMR (400 MHz, $CDCl_3$) δ 7.59 (d, *J* = 8.3 Hz, 2H), 7.39 (d, *J* = 8.3 Hz, 2H), 6.88 (d, *J* = 3.6 Hz, 1H), 6.84 (s, 1H), 6.72 (d, *J* = 3.6 Hz, 1H); $^{13}C\{1H\}$ NMR (101 MHz, $CDCl_3$) δ 171.8, 155.2, 147.8, 134.6, 129.2, 127.8, 125.5, 117.64, 117.63, 109.1 (br s), 108.1. For (*E*)-2-(1-bromo-2-isocyanovinyl)-5-(4-chlorophenyl)furan (***E-6a***): ^1H NMR (400 MHz, $CDCl_3$) δ 7.76 – 7.69 (m, 2H), 7.43 – 7.38 (m, 2H), 7.05 (d, *J* = 3.7

Hz, 1H), 6.80 (d, J = 3.7 Hz, 1H), 6.16 (s, 1H); $^{13}C\{1H\}$ NMR (101 MHz, $CDCl_3$) δ 170.4, 155.8, 147.0, 134.6, 129.2, 127.8, 125.9, 119.2, 117.9, 107.6, 107.1 (br s).

General Isocyanoalkyne Synthesis. Solid, dry *t*-BuOK (1.25 equiv.) was added to a 0 °C, THF solution (0.1M) of the bromoisocyanoalkene (1 equiv.). The reaction was monitored by ^1H NMR for complete consumption of the *Z*-bromoisocyanoalkene; unreacted *E*-bromoisocyanoalkene remained when the elimination was performed on *E/Z*-mixtures of bromoisocyanoalkenes. The crude alkyne was filtered through glass wool that was then washed with diethyl ether (1 x 10 mL). The combined filtrate was washed with brine (1 x 5 mL) and then with saturated, aqueous $NaHCO_3$. The organic phase was dried over Na_2SO_4 and then concentrated (150 mm/Hg, 20 °C) until the total volume was 1-2 mL. The sample was wet-loaded onto a 0.5 x 4.0 cm column packed with C-2 silica,[2] transferring any residue with a small volume (0.5 - 1 mL) of CH_2Cl_2. The column was eluted with pentane, the fractions were collected and then concentrated at 0 °C until the solvent volume was reduced to ~0.1 mL. Two purifications were performed for eliminations employing mixtures of *E*- and *Z*-bromoisocyanoalkenes because some isocyanoalkyne was always contaminated with *E*-bromoisocyanoalkene. The mixed fractions were collected and dry-loaded onto Celite (3 x the crude mass); the Celite-adsorbed crude reaction mixture was then placed under vacuum (9 mm Hg) at 0 °C for 1 min. The Celite-adsorbed sample was loaded onto a 0.5 x 4.0 cm precolumn containing a small amount of C-2 silica,[2] eluted with pentane, collected and concentrated at 0 °C until the solvent volume was reduced to ~0.1 mL. The flask containing the purified isocyanoalkyne was then placed in 0 °C ice-water bath and concentrated under a high vacuum until all the solvent was removed; the flask was gently swirled to prevent bumping with most of the solvent being removed within 1 min. The

sample was dissolved in $CDCl_3$ (1 mL) that was previously filtered from a suspension containing solid K_2CO_3 and then the ^1H and ^{13}C NMR were obtained.

Note: Several isocyanoalkynes are sensitive to heat, particularly when the solvent is being removed. Neat samples of several isocyanoalkynes tended to polymerize at room temperature whereas most samples were stable for weeks or months when stored at -20 °C. For example, phenyl isocyanoalkyne was obtained as a dark brown oil at 0 °C (Figure S1a) with warming to rt causing conversion to a dark polymer (Figure S1b) within 1 min. The polymerization does not appear to be associated with UV light as irradiation for 5 h with a 375 nm lamp did not cause any decomposition. Substrates with electron withdrawing groups tended to be more sensitive than those with electron donating groups. The tendency of isocyanoalkynes to polymerize can be minimized by taking care to ensure that only pure fractions of the isocyanoalkynes are concentrated, concentrating samples under a high vacuum in an ice-water bath for no longer than 1 min, and storing the pure isocyanoalkyne as CH_2Cl_2, benzene, or pentane solutions (0.1 mM) at -20 °C.

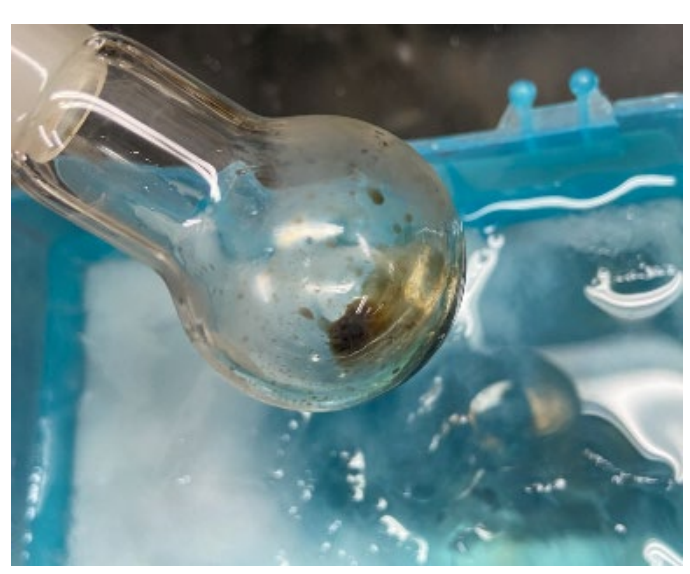

Figure S1a

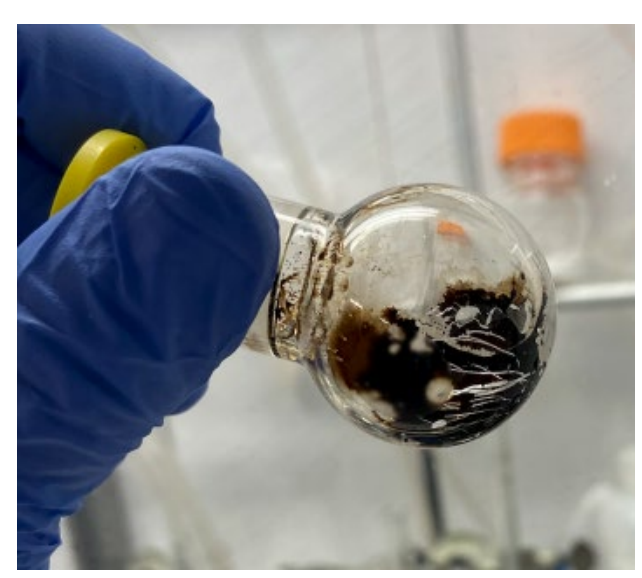

Figure S1b

Figure S1. Images showing the effect of concentrating solutions of **1d** at 0 °C (S1) and rt (S2)

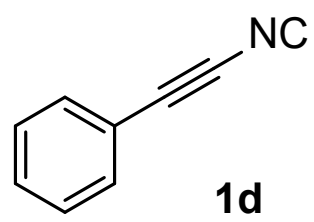

*(Isocyanoethynyl)benzene (**1d**)*. Following the general isocyanoalkyne procedure with (*Z*)-(1-bromo-2-isocyanovinyl)benzene[7] (24 mg, 0.12 mmol), with *t*-BuOK (16.2 mg, 0.15 mmol), for 0.5 h and purification on C-2 silica[2] (0.5 cm x 4 cm column) using pentanes as the eluant afforded 8.9 mg (60%) of (isocyanoethynyl)benzene as a dark red oil: IR 2926, 2240, 2063 cm^{-1}; ^{1}H NMR (400 MHz, $CDCl_3$): δ 7.53 – 7.49 (m, 2H), 7.47 – 7.42 (m, 1H), 7.39 – 7.33 (m, 2H); ^{13}C NMR (101 MHz, $CDCl_3$): 165.4, 132.9, 130.4, 128.7, 118.6, 57.1. Attempts to obtain HRMS data on a Waters Q-Tof Premier, time-of-flight, LCMS with electrospray ionization (ESI) and a scimaX FT-ICR instrument with an atmospheric pressure photoionization (APPI) have not been successful, probably because the isocyanide rapidly polymerized upon heating.[8] **Internal Standard Yield.** A yield was determined using mesitylene as an internal standard to provide a more accurate yield that takes into account the degradation of **1d** on the C2-silica chromatography matrix.[2] (Isocyanoethynyl)benzene was prepared following the general isocyanoalkyne procedure with (*Z*)-(1-bromo-2-isocyanovinyl)benzene (100 mg, 0.48 mmol) and *t*-BuOK (0.95 equiv), with mesitylene (15 mg, $mmol^{-1}$) added as an internal standard, except that the final solution of (isocyanoethynyl)benzene was analyzed by ^{1}H NMR for a yield and not concentrated. A yield of 70% was calculated using the integral value of the mesitylene resonance at 6.78 ppm (s) and the (isocyanoethynyl)benzene resonance at 7.34 - 7.39 ppm (multiplet) according to the following equation:

$$Alkyneisocyanide_{mass}(mg) = (isocyanoethynyl)benzene\ _{MW}[\frac{Mesitylene}{120.19g/mol} * \frac{Intetration\ Ratio}{3}]$$

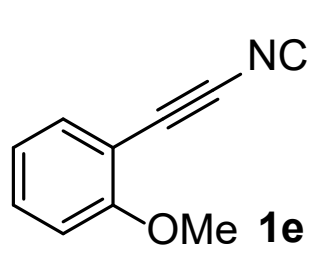

*1-(Isocyanoethynyl)-2-methoxybenzene (**1e**)*. Following the general isocyanoalkyne procedure with *(Z)*-1-(1-bromo-2-isocyanovinyl)-2-methoxybenzene[7] (228 mg,

0.96 mmol) and *t*-BuOK (134.3 mg, 1.20 mmol), for 1.5 h, with purification on C-2 silica[2] (0.5 cm x 4 cm column) using pentanes as the eluant afforded 96.8 mg (64%) of **1e** as a clear oil: IR (ATR) 2937, 2839, 2249, 2061 cm^{-1} ^{1}H NMR (500 MHz, $CDCl_3$) δ 7.45 (dd, *J* = 7.7, 1.7 Hz, 1H), 7.43 – 7.38 (m, 1H), 6.96 – 6.92 (m, 1H), 6.90 (d, *J* = 8.5 Hz, 1H), 3.90 (s, 3H); ^{13}C NMR (101 MHz, $CDCl_3$): δ 165.4, 161.9, 134.7, 131.9, 120.6, 110.8, 107.8, 55.8, 54.1; HRMS (+EI) m/z [M^+] Calcd. for $C_{10}H_7NO$, 157.0533 found 157.0511.

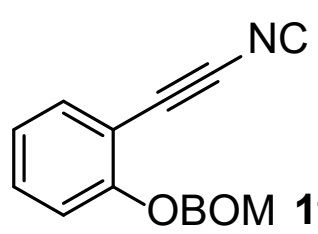
1f

*1-((Benzyloxy)methoxy)-2-(isocyanoethynyl)benzene (**1f**).* Following the general isocyanoalkyne procedure with (*Z*)-1-((benzyloxy)methoxy)-2-(1-bromo-2-isocyanovinyl)benzene[7] (19 mg, 55 μmol) and *t*-BuOK (7.8 mg, 69 μmol), for 2 h, with purification on C-2 silica[2] (0.5 cm x 4 cm column) using 2% CH_2Cl_2/Hexane as the eluant afforded 13 mg (89%) of **1f** as a yellow oil; IR (ATR): 2919, 2857, 2062, 1598 cm^{-1}; ^{1}H NMR (400 MHz, $CDCl_3$) δ 7.49 – 7.44 (m, 1H), 7.42 – 7.29 (m, 6H), 7.22 (dd, *J* = 8.5, 1.1 Hz, 1H), 7.03 – 6.96 (m, 1H), 5.38 (s, 2H), 4.76 (s, 2H); ^{13}C NMR (101 MHz, $CDCl_3$): δ 165.6, 159.7, 136.8, 134.6, 131.9, 128.5, 128.0, 128.0, 121.8, 114.7, 108.9, 92.6, 70.5 (t, *J* = 2.5 Hz), 54.1; HRMS (+ESI) m/z [M+Na]$^+$ Calcd. for 286.0838 found 286.0840.

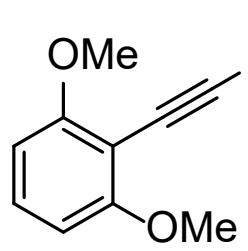
1g

*2-(Isocyanoethynyl)-1,3-dimethoxybenzene (**1g**).* Following the general isocyanoalkyne procedure with (*Z*)-2-(1-bromo-2-isocyanovinyl)-1,3-dimethoxybenzene[7] (100 mg, 0.37 mmol) and *t*-BuOK (52.3 mg, 0.47 mmol), for 2 h, with purification on C-2 silica[2] (0.5 cm x 4 cm column) using pentane as the eluant afforded 57.2 mg (82%) of **1g** as a white solid that decomposed upon heating: IR (ATR) 2966, 2930, 2120, 2061 cm^{-1}; ^{1}H NMR (400 MHz, $CDCl_3$) δ (t, *J* = 8.4 Hz, 1H), 6.52 (d, *J* = 8.4 Hz, 2H), 3.87 (s, 6H); ^{13}C

NMR (101 MHz, $CDCl_3$): δ 165.5, 163.4, 132.0, 103.4, 97.2, 56.1, 55.6, 50.9; HRMS (+EI) m/z [M^+] Calcd. for $C_{11}H_9NO_2$, 187.0633 found 187.0630.

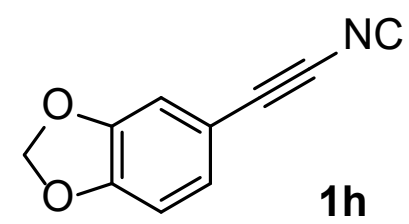
1h

5-(Isocyanoethynyl)benzo[d][1,3]dioxole ***(1h)***. Following the general isocyanoalkyne procedure with (*Z*)-5-(1-bromo-2-isocyanovinyl)benzo[d][1,3]dioxole[7] (43.2 mg, 0.17 mmol) and *t*-BuOK (24.1 mg, 0.22 mmol), for 1.5 h, with purification on a column packed with C-2 silica[2] (0.5 cm x 4 cm using 100% Hexanes as the eluant) afforded 22 mg (75%) of **1h** as a white solid that polymerized upon heating: IR (ATR) 2916, 2795, 2247, 2067 cm^{-1}; ^1H NMR (500 MHz, $CDCl_3$) δ 7.07 (dd, *J* = 8.0, 1.6 Hz, 1H), 6.92 (d, *J* = 1.6 Hz, 1H), 6.79 (d, *J* = 8.0 Hz, 1H), 6.02 (s, 2H); ^{13}C NMR (126 MHz, $CDCl_3$) δ 172.5, 165.0, 149.8, 147.7, 128.5, 112.3, 111.4, 108.8, 101.8 (t, *J* = 4.5 Hz), 57.3; HRMS (+APPI) m/z [M^+] Calcd. for $C_{10}H_{15}NO_2$, 171.0324 found 171.0315.

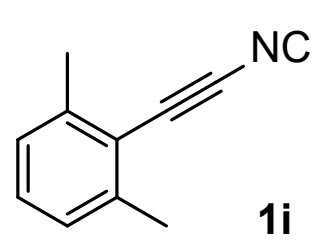
1i

2-(Isocyanoethynyl)-1,3-dimethylbenzene ***(1i)***. Following the general isocyanoalkyne procedure with (*Z*)-2-(1-bromo-2-isocyanovinyl)-1,3-dimethylbenzene[7] (100 mg, 0.42 mmol) and *t*-BuOK (59.4 mg, 0.53 mmol) for 2 h, with purification on C-2 silica[2] (0.5 cm x 4 cm column) using pentane as the eluant afforded 32 mg (49%) of **1i** as a clear oil: IR (ATR): 3066, 2922, 2854, 2059 cm^{-1}; ^1H NMR (400 MHz, $CDCl_3$) δ 7.24 – 7.18 (m, 1H), 7.06 (d, *J* = 7.7 Hz, 1H), 2.43 (s, 6H); ^{13}C NMR (101 MHz, $CDCl_3$): δ 166.3, 142.6, 129.8, 127.1, 118.4, 55.2, 20.9; ^{13}C NMR (101 MHz, DMSO) δ 166.8, 142.8, 130.8, 127.7, 117.6, 75.9, 55.8; HRMS (+ESI) m/z [M^+] Calcd. for $C_{11}H_9N$, 155.0735 found 155.0746.

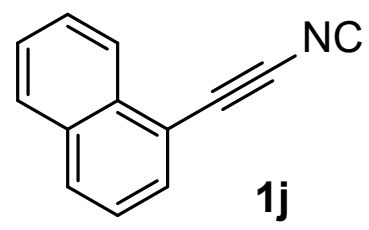
1j

*1-(Isocyanoethynyl)naphthalene (**1j**)*. Following the general isocyanoalkyne procedure with (*Z*)-1-(1-bromo-2-isocyanovinyl)naphthalene[7] (71 mg, 0.28 mmol) and *t*-BuOK (38.6 mg, 0.34 mmol), for 2 h, with purification on a column packed with C-2 silica[2] (0.5 cm x 4 cm column using pentane as the eluant) afforded 27 mg (55 %) of **1j** as a white solid that polymerized upon heating: IR (ATR): 3058, 2925, 2257, 2061 cm^{-1}; ^{1}H NMR (500 MHz, $CDCl_3$) δ 8.20 (d, *J* = 8.3 Hz, 1H), 7.94 (d, *J* = 8.3 Hz, 1H), 7.89 (d, *J* = 8.3 Hz, 1H), 7.80 – 7.76 (m, 1H), 7.66 – 7.61 (m, 1H), 7.60 – 7.54 (m, 1H), 7.48 – 7.43 (m, 1H); ^{13}C NMR (101 MHz, $CDCl_3$): δ 166.5, 133.8, 133.1, 133.0, 131.0, 128.7, 127.7, 127.0, 125.4, 125.1, 116.1, 55.8, 53.4; HRMS (+APCI) m/z [$M+H^+$] Calcd. for $C_{13}H_8N$ 178.0651, found 178.0654. **1.26 mmol scale:** Following the general isocyanoalkyne procedure with (*Z*)-1-(1-bromo-2-isocyanovinyl)naphthalene[7] (672 mg, 2.6 mmol) and *t*-BuOK (365.2 mg, 3.25 mmol), for 2 h, with purification on a column packed with C-2 silica[2] (0.5 cm x 4 cm column using pentane as the eluant) afforded 223.8 mg (49 %, 1.3 mmol) of **1j** identical to material previously isolated.

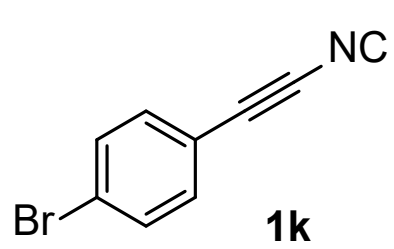
1k

*1-Bromo-4-(isocyanoethynyl)benzene (**1k**)*. Following the general isocyanoalkyne procedure with (*Z*)-1-bromo-4-(1-bromo-2-isocyanovinyl)-benzene[7] (160 mg, 0.56 mmol) and *t*-BuOK (78.2 mg, 0.69 mmol), for 1.5 h with purification on C-2 silica[2] (0.5 cm x 4 cm column) using pentanes as the eluant afforded 64.2 mg (56%) of 1-bromo-4-(isocyanoethynyl)benzene (**1k**) as a pink solid that polymerized upon heating: IR (ATR) 2923, 2852, 2222, 2061 cm^{-1}; ^{1}H NMR (400 MHz, $CDCl_3$) δ 7.53 – 7.49 (m, 2H), 7.39 – 7.34 (m, 2H); ^{13}C NMR (101 MHz, DMSO): δ 166.4, 135.4, 132.6, 125.5, 117.0, 57.1; HRMS (+EI) m/z [M^+] Calcd. for C_9H_4BrN 204.9527, found 204.9520.

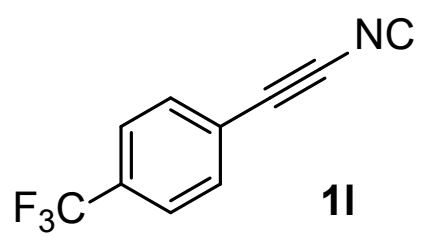
1l

*1-(Isocyanoethynyl)-4-(trifluoromethyl)benzene (**1l**)*. Following the general isocyanoalkyne procedure with (*Z*)-1-(1-bromo-2-isocyanovinyl)-4-(trifluoromethyl)benzene[7] (48 mg, 0.17 mmol) and *t*-BuOK (24 mg, 0.22 mmol), for 1.5 h, with purification on C-2 silica[2] (0.5 cm x 4 cm column) using pentanes as the eluant afforded 12.4 mg (37%) of 1-(isocyanoethynyl)-4-(trifluoromethyl)benzene as a yellow oil: IR 2925, 2854, 2064 1619 cm^{-1}; ^1H NMR (400 MHz, DMSO-d_6) δ 7.88 (d, *J* = 8.7 Hz, 2H), 7.83 (d, *J* = 8.7 Hz, 2H); ^{13}C NMR (101 MHz, DMSO d6) δ 167.0, 134.4, 131.1 (q, *J* = 32.3 Hz), 126.3 (q, *J* = 3.8 Hz), 124.0 (q, *J* = 272.7 Hz), 122.2 (q, *J* = 1.7 Hz), 70.0, 56.4; HRMS (+EI) m/z [M^+] Calcd. for $C_{10}H_4F_3N$ 195.0296, found 195.0310. A yield was determined using mesitylene as an internal standard to provide a more accurate yield that accounts for the degradation of **1l** on the C2-silica chromatography matrix.[2] 1-(Isocyanoethynyl)-4-(trifluoromethyl)benzene (**1l**) was prepared following the general isocyanoalkyne procedure with (*Z*)-1-(1-bromo-2-isocyanovinyl)-4-(trifluoromethyl)benzene (120 mg, 0.44 mmol) and *t*-BuOK (61.0 mg, 0.54 mmol), with mesitylene (13.2 mg, 0.11 mmol) added as an internal standard, except that the final solution of 1-(isocyanoethynyl)-4-(trifluoromethyl)benzene (**1l**) was analyzed by ^1H NMR for a yield and not concentrated. A yield of 58% was calculated using the integral value of the mesitylene resonance at 6.78 ppm (s) and the (isocyanoethynyl)benzene resonance at 7.64 ppm (singlet) according to the following equation:

$$Alkyneisocyanide_{mass}(mg) = (isocyanoethynyl)benzene\ _{MW}[\frac{Mesitylene}{120.19g/mol} * \frac{Intetration\ Ratio}{3}]$$

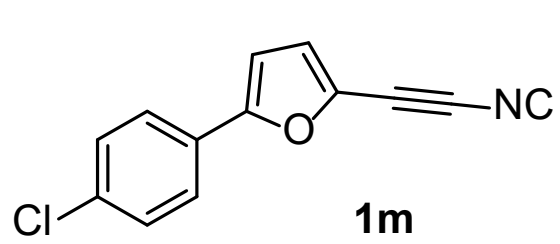
1m

*2-(4-Chlorophenyl)-5-(isocyanoethynyl)furan (**1m**)*. Following the general isocyanoalkyne procedure with (*Z*)-2-(1-bromo-2-isocyanovinyl)-

5-(4-chlorophenyl)furan (***Z*-6m**, 21 mg, 68 μmol) and *t*-BuOK (9.6 mg, 85 μmol), for 3.5 h, with purification on C-2 silica[2] (0.5 cm x 4 cm column) using 2% CH_2Cl_2/Hexanes as the eluant afforded 7.6 mg (49%) of **1m** as a white solid that polymerized upon heating: IR (ATR) 2924, 2854, 2057, 1588 cm^{-1}; ^{1}H NMR (400 MHz, $CDCl_3$) δ 7.65 – 7.57 (m, 2H), 7.43 – 7.35 (m, 2H), 6.92 (d, J = 3.6 Hz, 1H), 6.66 (d, J = 3.6 Hz, 1H); ^{13}C NMR (101 MHz, DMSO d_6): δ 171.2, 155.9, 134.0, 132.5, 129.7, 128.0, 126.5, 124.7, 108.9, 77.2, 49.0; HRMS (+EI) m/z [M^+] Calcd. for $C_{13}H_6ClNO$ 227.0138, found 227.0147.

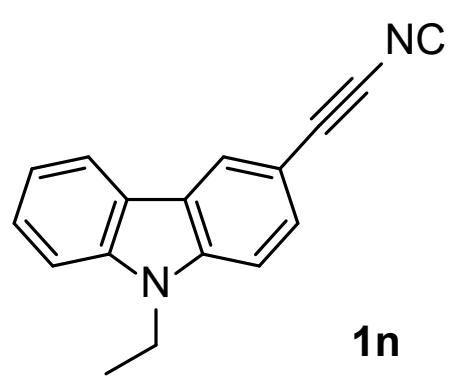

1n

9-Ethyl-2-(isocyanoethynyl)-9H-carbazole ***(1n)***. Following the general isocyanoalkyne procedure with (*Z*)-2-(1-bromo-2-isocyanovinyl)-9-ethyl-9H-carbazole[7] (29.3 mg, 90 μmol) and *t*-BuOK (12.7 mg, 0.11 mmol), for 2 h, with purification on a column packed with C-2 silica[2] (0.5 cm x 4 cm using 100% Hexanes as the eluant) afforded 14.2 mg (65%) of **1n** as a white solid that polymerized upon heating: IR 2925, 2858, 2253, 2059 cm^{-1}; ^{1}H NMR (400 MHz, $CDCl_3$) δ 8.27 (d, J = 1.7 Hz, 1H), 8.08 (d, J = 7.8 Hz, 1H), 7.58 (dd, J = 8.5, 1.7 Hz, 1H), 7.55 – 7.50 (m, 1H), 7.43 (d, J = 8.3 Hz, 1H), 7.37 (d, J = 8.4 Hz, 1H), 7.32 – 7.27 (m, 1H), 4.37 (q, J = 7.2 Hz, 2H), 1.44 (t, J = 7.2 Hz, 3H); ^{13}C NMR (101 MHz, $CDCl_3$) δ 164.1, 140.6, 140.4, 130.1, 126.6, 125.9, 123.1, 122.1, 120.6, 119.9, 108.9, 108.8, 107.8, 59.1, 37.8, 13.8; HRMS (+EI) m/z [M^+] Calcd. for $C_{17}H_{12}N_2$ 244.0995, found 244.1007.

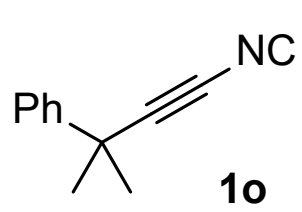

1o

(4-Isocyano-2-methylbut-3-yn-2-yl)benzene ***(1o)***. Following the general isocyanoalkyne procedure with (*Z*)-(3-bromo-4-isocyano-2-methylbut-3-en-2-yl)benzene[7] (47.6 mg, 0.19 mmol) and *t*-BuOK (26.7 mg, 0.24 mmol), for 1.5 h, with purification on a column packed with C-2 silica[2] (0.5 cm x 4 cm using 100% Hexanes as the eluant) afforded

28.2 mg (88%) of **1o** as a clear oil: IR (ATR): 2979, 2926, 2120, 2066 cm^{-1}; ^{1}H NMR (400 MHz, $CDCl_3$) δ 7.48 – 7.43 (m, 2H), 7.40 – 7.34 (m, 2H), 7.31 – 7.26 (m, 1H), 1.63 (s, 6H); ^{13}C NMR (101 MHz, $CDCl_3$) δ 160.5, 144.4, 128.6, 127.1, 125.2, 64.8, 62.2, 35.4, 30.7; HRMS (+APPI) m/z [M^+] Calcd. for $C_{12}H_{11}N$ 169.0886, found 169.0895.

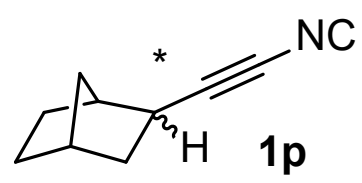

1p

(1S,4R*)-2-(Isocyanoethynyl)bicyclo[2.2.1]heptane (**1p**)*. Following the general isocyanoalkyne procedure with (*Z*)-2-(1-bromo-2-isocyanovinyl)bicyclo[2.2.1]heptane[7] (43.7 mg, 0.19 mmol) and *t*-BuOK (27.1 mg, 0.24 mmol), for 4 h, with purification on a column packed with C-2 silica[2] (0.5 cm x 4 cm using 100% Hexanes as the eluant) afforded 1:1 mixture of diastereomers of **1p**, 18.6 mg (66%), as a clear oil: IR (ATR) 2995, 2873, 2264, 2065 cm^{-1}; ^{1}H NMR (400 MHz, $CDCl_3$) δ 2.68 – 2.59 (m, 1H), 2.38 – 2.24 (m, 4H), 1.97 – 1.88 (m, 1H), 1.77 – 1.07 (m, 5H); ^{13}C NMR (101 MHz, $CDCl_3$) δ 158.6 (157.9), 63.7 (62.8), 43.2 (41.2), 39.1 (38.1), 37.2 (36.84), 36.82 (36.1), 31.7 (30.4), 29.4 (28.7), 28.5 (24.3); HRMS (+APPI) m/z [M^+] Calcd. for $C_{10}H_{11}N$ 145.0886, found 145.0894.

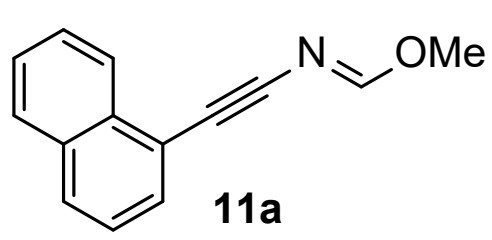

11a

*Methyl (E)-N-(naphthalen-1-ylethynyl)formimidate (**11a**).* Neat $Et_3N{\cdot}HCl$ (22.2 mg, 0.16 mmol) was added to a methanolic solution (0.1 M) of 1-(isocyanoethynyl)naphthalene (**1j**, 19 mg, 0.11 mmol). After 48 h, the reaction was diluted with CH_2Cl_2, water was added, and then the phases were separated. The aqueous phase was washed with CH_2Cl_2 (3 x 10 mL) and the combined organic layer was then washed sequentially, with H_2O and brine. The resulting crude was dried (Na_2SO_4), concentrated, and then purified by silica gel chromatography (1 x 4 cm column) to provide 10.2 mg (45%) of **11a** as a clear oil: IR 3055, 2945, 2207, 1616 cm^{-1}; ^{1}H NMR (400 MHz, $CDCl_3$) δ 8.39 – 8.30 (m, 2H), 7.83 (d, *J* = 7.9 Hz, 1H),

7.77 (d, *J* = 8.2 Hz, 1H), 7.61 (d, *J* = 7.1 Hz, 1H), 7.57 – 7.47 (m, 2H), 7.41 (t, *J* = 7.7 Hz, 1H), 3.94 (s, 3H); ^{13}C NMR (101 MHz, $CDCl_3$) δ 164.3, 133.3, 133.2, 129.6, 128.1, 127.6, 126.4, 126.3, 126.2, 125.3, 122.0, 92.6, 71.0, 54.6; HRMS (+EI) m/z [M^+] Calcd. for $C_{14}H_{11}NO$ 209.0841, found 209.0833.

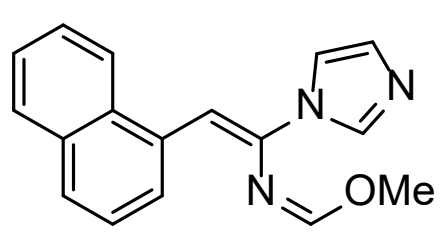

13a

*Methyl (E)-N-((Z)-1-(1H-imidazol-1-yl)-2-(naphthalen-1-yl)vinyl)formimidate (**13a**)*. Imidazole (15.4 mg, 0.23 mmol) and $Et_3N{\cdot}HCl$ (15.5 mg, 0.11 mmol) were added to a methanolic solution (0.1 M) of naphthylisocyanoalkyne (**1j**, 20 mg, 0.11 mmol). After 16 h, the reaction was diluted with CH_2Cl_2, water was added, and then the phases were separated. The aqueous phase was washed with CH_2Cl_2 (3 x 10 mL) and the combined organic layer was then washed sequentially with H_2O and brine, dried (Na_2SO_4), and concentrated. Purification of the crude azadiene on a silica gel column (1 x 4 cm) provided 26 mg (83%) of **13a** as yellow oil: IR 3059, 2944, 2850, 1630 cm^{-1}; ^{1}H NMR (400 MHz, $CDCl_3$) δ 8.06 – 7.99 (m, 1H), 7.91 (br s, 1H), 7.89 – 7.84 (m, 1H), 7.77 (d, *J* = 8.2 Hz, 1H), 7.67 (d, *J* = 7.2 Hz, 1H), 7.60 – 7.49 (m, 3H), 7.45 (t, *J* = 7.7 Hz, 1H), 7.31 (br s, 1H), 7.19 (br s, 1H), 6.63 (s, 1H), 3.81 (s, 3H); ^{13}C NMR (101 MHz, $CDCl_3$) δ 159.7, 139.3, 136.0, 133.7, 131.8, 131.0, 130.0, 128.7, 127.7, 127.6, 126.2, 126.0, 125.4, 124.3, 117.5, 104.4, 54.4; HRMS (+ESI) m/z [$M+H^+$] Calcd. for $C_{17}H_{15}N_3O$ 278.1293, found 278.1302.

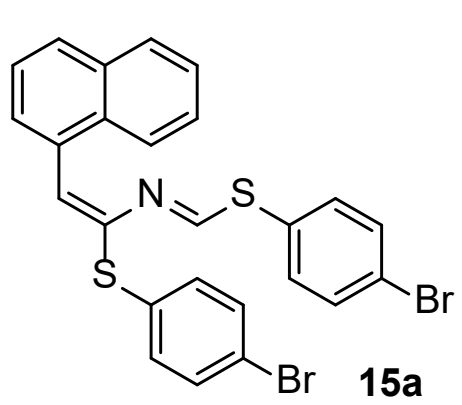

15a

*4-Bromophenyl-(E)-N-((Z)-1-((4-bromophenyl)thio)-2-(naphthalen-1-yl)vinyl)methanimidothioate (**15a**)*. A CH_2Cl_2 solution (0.1 M) 4-bromobenzenethiol (23.5 mg, 0.13 mmol) was added to a -78 °C, CH_2Cl_2 solution (0.1 M) of naphthylisocyanoalkyne (**1j**, 10 mg, 56 µmol), $Et_3N{\cdot}HCl$ (7.7 mg, 56 µmol).

After 1 h, the reaction was allowed to warm to rt and then passed through a 1 cm x 1 cm pad of silica gel. The resulting filtrate was concentrated and was then purified on a silica gel column (2 cm x 1 cm column using 2% CH_2Cl_2/ Hexanes as the eluant) to provide 22 mg (70% yield) of **15a** as a yellow solid mp 82.2 - 86.4 °C: IR 3058, 2923, 1550 cm^{-1}; ^1H NMR (400 MHz, $CDCl_3$) δ 8.76 (s, 1H), 8.08 (d, *J* = 7.8 Hz, 1H), 7.88 – 7.83 (m, 2H), 7.81 (d, *J* = 8.2 Hz, 1H), 7.55 (s, 1H), 7.53 – 7.46 (m, 2H), 7.45 – 7.40 (m, 4H), 7.36 (t, *J* = 7.8 Hz, 1H), 7.20 – 7.15 (m, 2H), 7.13 – 7.08 (m, 2H); ^{13}C NMR (101 MHz, $CDCl_3$) δ 163.5, 137.5, 134.9, 134.1, 133.5, 132.5, 132.3, 131.7, 131.0, 130.7, 129.9, 129.5, 128.8, 128.7, 128.3, 126.4, 125.6, 125.3, 123.7, 123.6, 119.9; HRMS (+ESI) m/z [$M+H^+$] Calcd. for $C_{25}H_{18}Br_2NS_2$ 553.9247, found 553.9230.

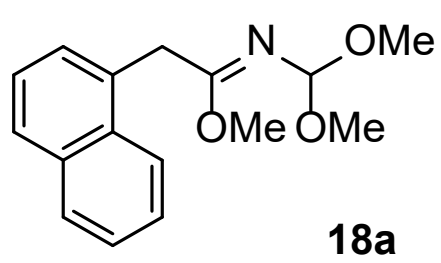

18a

*Methyl (Z)-N-(dimethoxymethyl)-2-(naphthalen-1-yl)acetimidate (**18a**).* A methanolic solution (0.5 M) of NaOMe (54 µL, 27 µmol) was added to a methanolic solution (0.1 M) of **1j** (9.6 mg, 54 µmol). After 3 h, one drop of $NaHCO_3$ was added, the reaction mixture was concentrated, and the resulting crude material was dissolved in EtOAc. The solution was washed with brine (1 mL) and was then concentrated to provide 22.3 mg (68 %) of **18a** as a pure, clear oil: IR 3045, 2945, 2833, 1670 cm^{-1}; ^1H NMR (400 MHz, $CDCl_3$) δ 7.95 (d, *J* = 8.2 Hz, 1H), 7.89 – 7.84 (m, 1H), 7.78 (d, *J* = 8.2 Hz, 1H), 7.56 – 7.47 (m, 2H), 7.45 – 7.39 (m, 1H), 7.31 (d, *J* = 7.1 Hz, 1H), 5.59 (s, 1H), 4.12 (s, 2H), 3.71 (s, 3H), 3.37 (s, 6H); ^{13}C NMR (101 MHz, C_6D_6) δ 164.9, 133.9, 132.3, 131.7, 128.7, 127.5, 126.8, 126.1, 125.5, 125.3, 123.6, 105.6, 52.7, 49.9, 34.1; HRMS (+EI) m/z [$M-CH(OMe)_2^+$] Calcd. for 198.0913, found 198.0919.

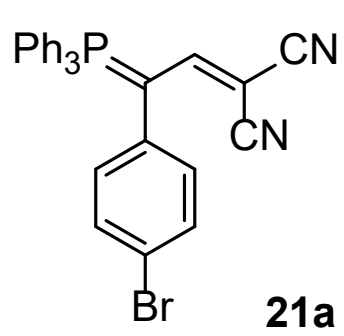

21a

*2-(2-(4-Bromophenyl)-2-(triphenyl-l5-phosphaneylidene)ethylidene)-malononitrile (**21a**).* Solid triphenylphosphine (28.0 mg, 0.11 mmol), neat

malonitrile (7.1 mg, 0.11 mmol), and neat mesitylene (9.5 mg, 80 μmol) were added to a CH_2Cl_2 solution (0.1 M) of **1k** (20 mg, 97 μmol). After 5 h, the crude mixture was concentrated and then redissolved in $CDCl_3$ to obtain a yield based on ^{1}H NMR integration. A yield of 78% was calculated using the integral value of the mesitylene resonance at 6.78 ppm (s) and the resonance of **21a** at 6.72 ppm (doublet) according to the following equation:

$$Product_{mass}(mg) = (product\ _{MW}[\frac{Mesitylene}{120.19g/mol} * \frac{Intetration\ Ratio}{3}$$

Purification on C-2 silica[2] (2 cm x 1 cm column using 100% CH_2Cl_2 as the eluant) provided 21.2 mg **21a** (47% yield) as yellow solid: mp 289.9 - 290.4 °C; IR 3060, 2194, 2173, 1530 cm^{-1}; ^{1}H NMR (400 MHz, $CDCl_3$) δ 7.76 – 7.69 (m, 3H), 7.60 – 7.53 (m, 6H), 7.45 – 7.36 (m, 6H), 7.28 (d, *J* = 8.4 Hz, 2H), 6.84 (dd, *J* = 8.4, 2.0 Hz, 2H), 6.72 (d, *J* = 19.3 Hz, 1H); ^{13}C NMR δ 152.3 (d, *J* = 18.3 Hz), 135.0 (d, *J* = 4.6 Hz), 134.03 (d, *J* = 3.4 Hz), 134.02 (d, *J* = 9.6 Hz), 131.9 (d, *J* = 9.6 Hz), 131.4 (d, *J* = 2.0 Hz), 129.6 (d, *J* = 12.3 Hz), 123.1, 122.5 (d, *J* = 3.3 Hz), 122.4, 121.5, 118.2; ^{31}P{^{1}H} NMR (161.9 MHz, $CDCl_3$): δ 22.5 (s) ppm; HRMS (+ESI) m/z [M+H$^+$] Calcd. for $C_{29}H_{21}BrNP$ 507.0626, found 507.0632.

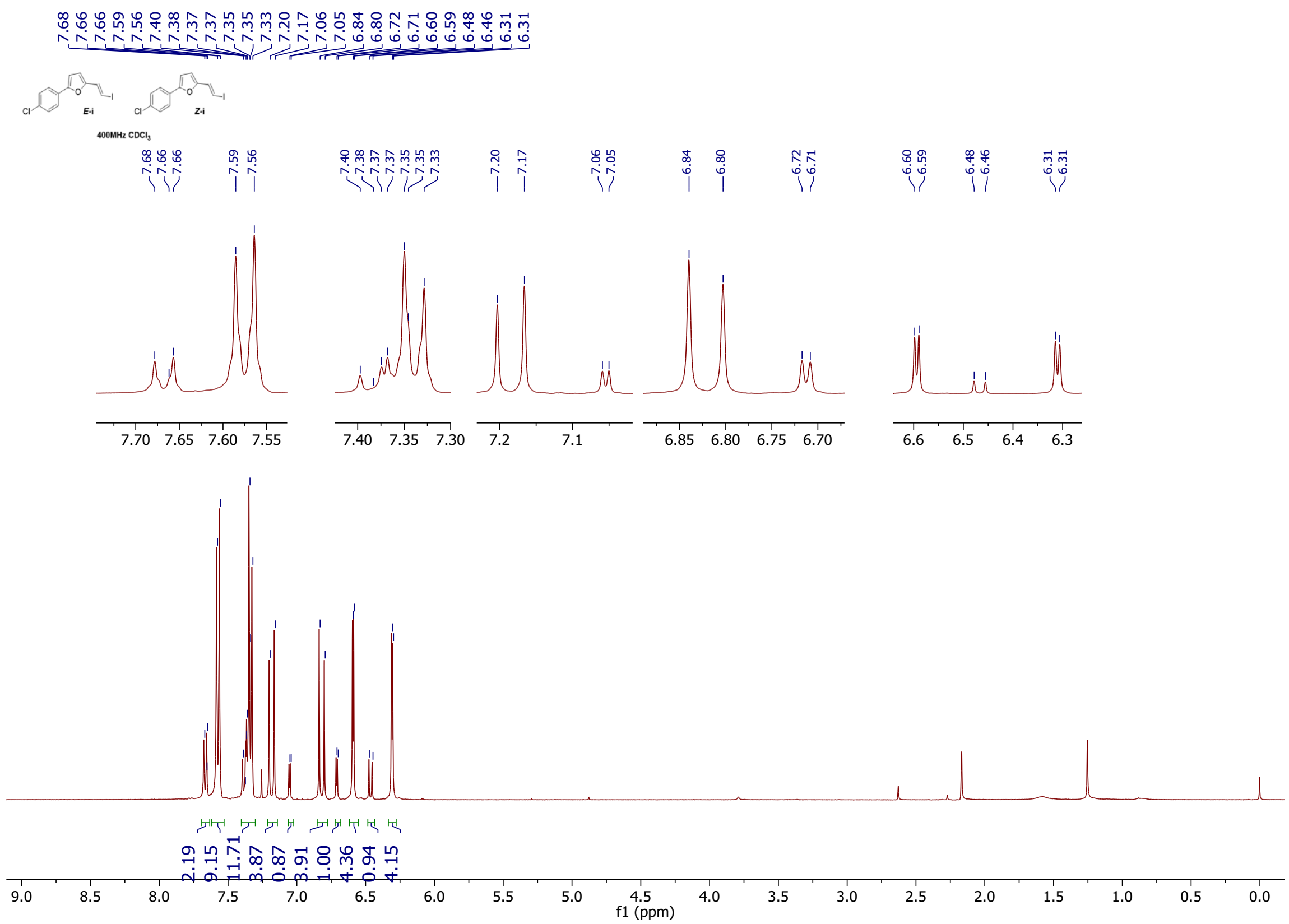
7.68 7.66 7.66 7.59 7.56 7.40 7.38 7.37 7.37 7.35 7.35 7.33 7.20 7.17 7.06 7.05 6.84 6.80 6.72 6.71 6.60 6.59 6.48 6.46 6.31 6.31
E-I
Z-I
400MHz CDCl3
2.19 9.15 11.71 3.87 0.87 3.91 1.00 4.36 0.94 4.15
f1 (ppm)

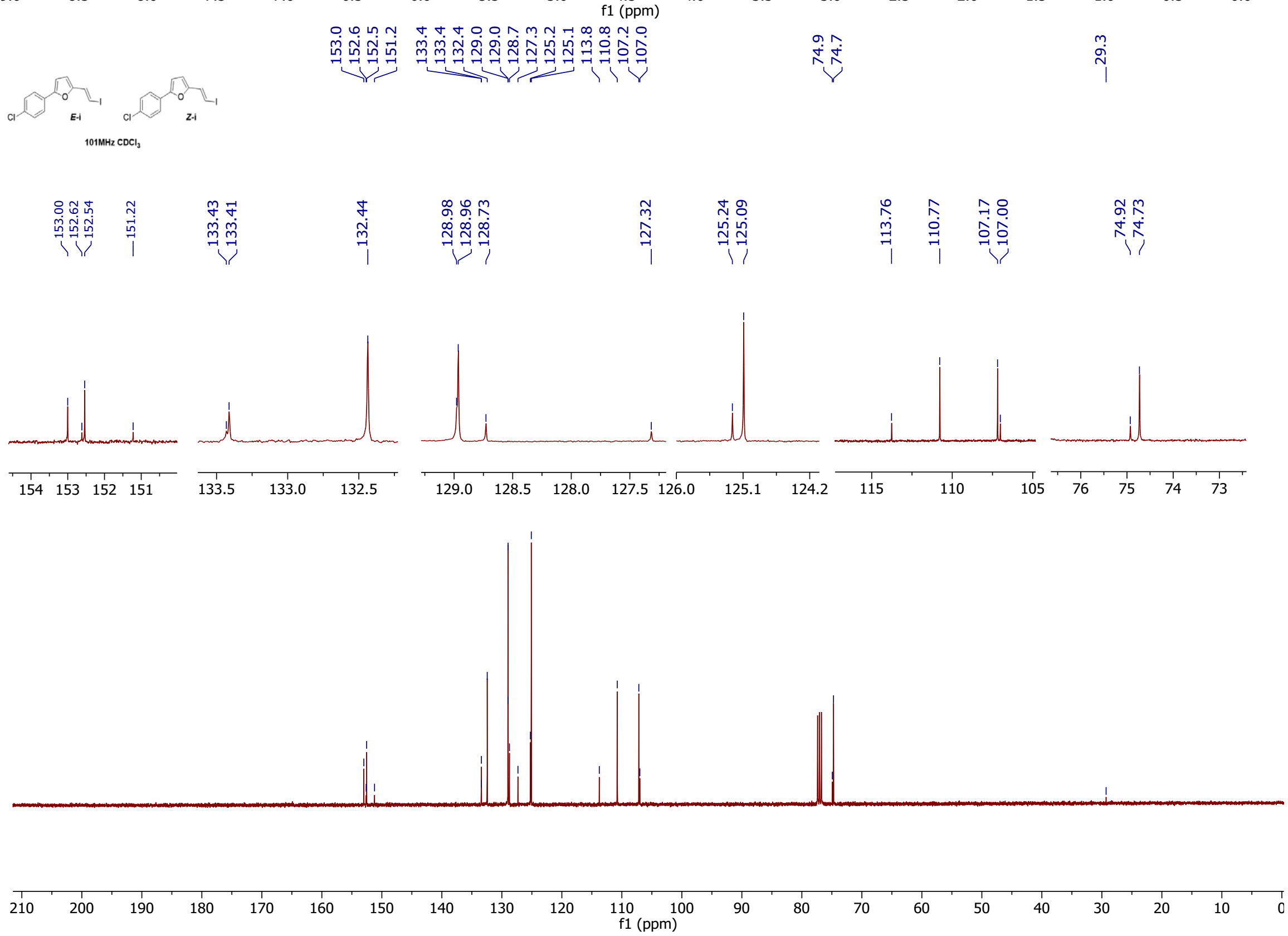
153.0 152.6 152.5 151.2 133.4 133.4 132.4 129.0 129.0 128.7 127.3 125.2 125.1 113.8 110.8 107.2 107.0 74.9 74.7 29.3
E-I
Z-I
101MHz CDCl3
153.00 152.62 152.54 151.22 133.43 133.41 132.44 128.98 128.96 128.73 127.32 125.24 125.09 113.76 110.77 107.17 107.00 74.92 74.73
f1 (ppm)

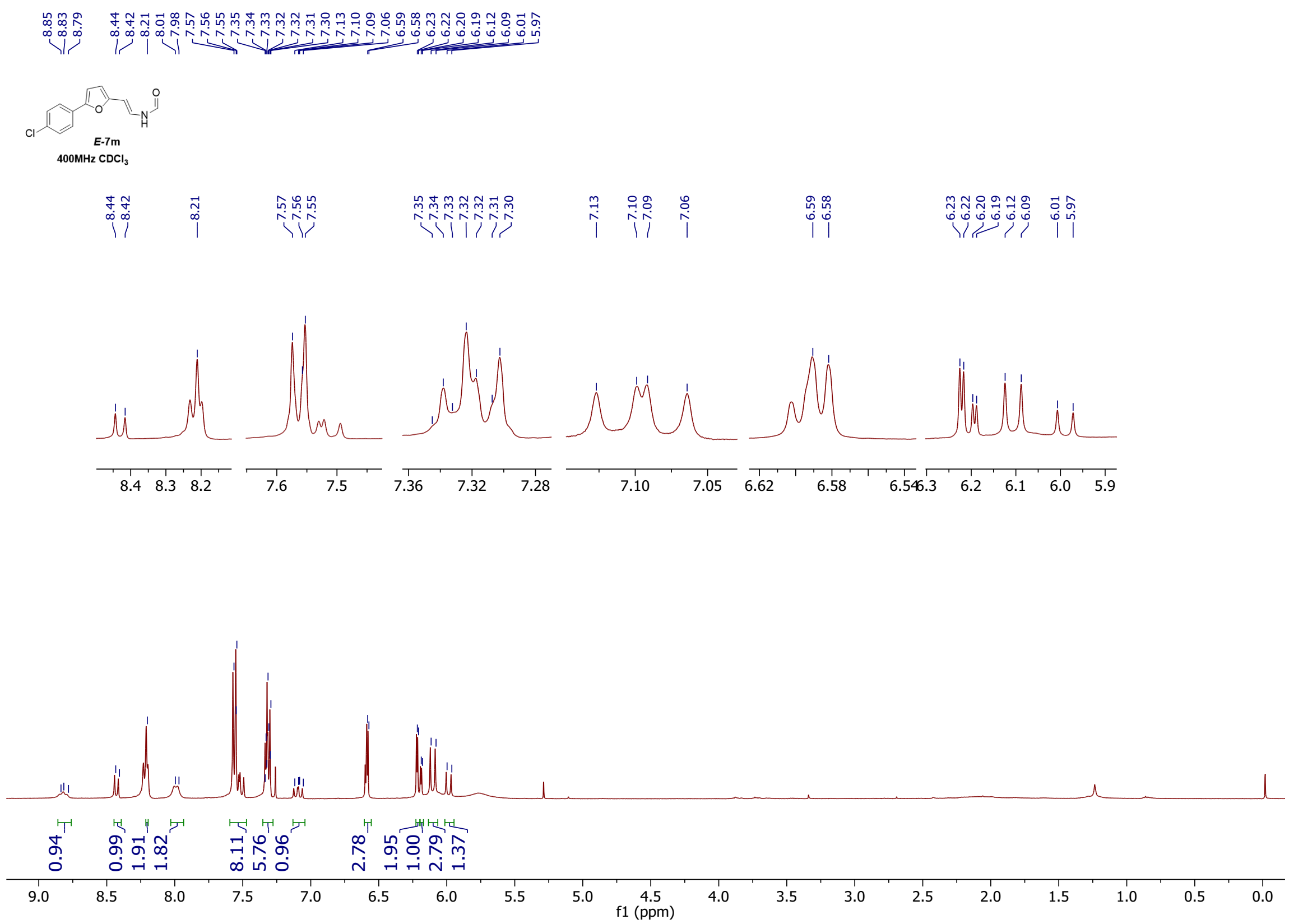
Cl
O
N
H
O
E-7m
400MHz CDCl3
f1 (ppm)

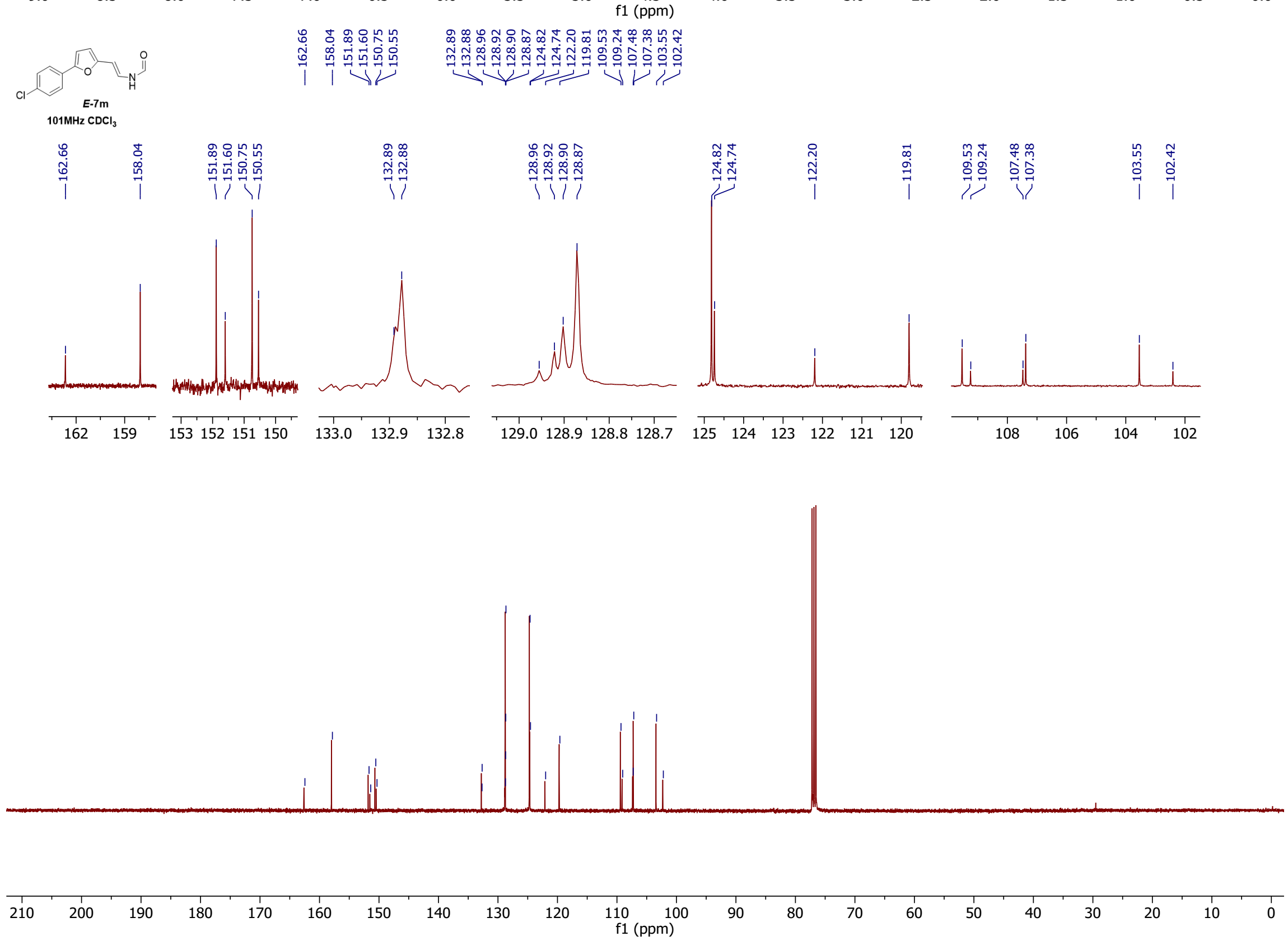
Cl
O
N
H
O
E-7m
101MHz CDCl3
f1 (ppm)

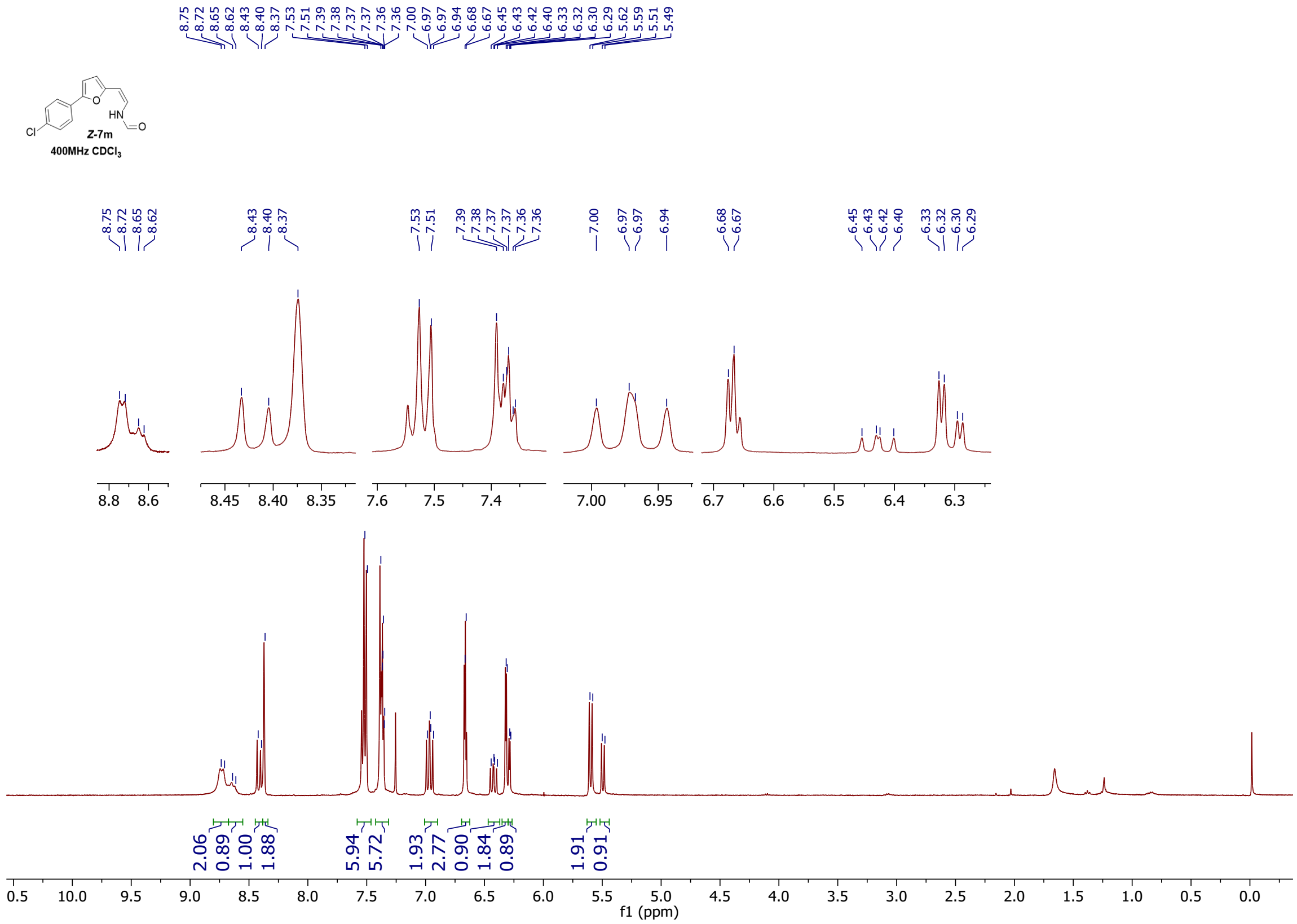

8.75 8.72 8.65 8.62 8.43 8.40 8.37 7.53 7.51 7.39 7.38 7.37 7.37 7.36 7.36 7.00 6.97 6.97 6.94 6.68 6.67 6.45 6.43 6.42 6.40 6.33 6.32 6.30 6.29 5.62 5.59 5.51 5.49
Cl
HN
O
Z-7m
400MHz CDCl3
2.06 0.89 1.00 1.88 5.94 5.72 1.93 2.77 0.90 1.84 0.89 1.91 0.91
f1 (ppm)

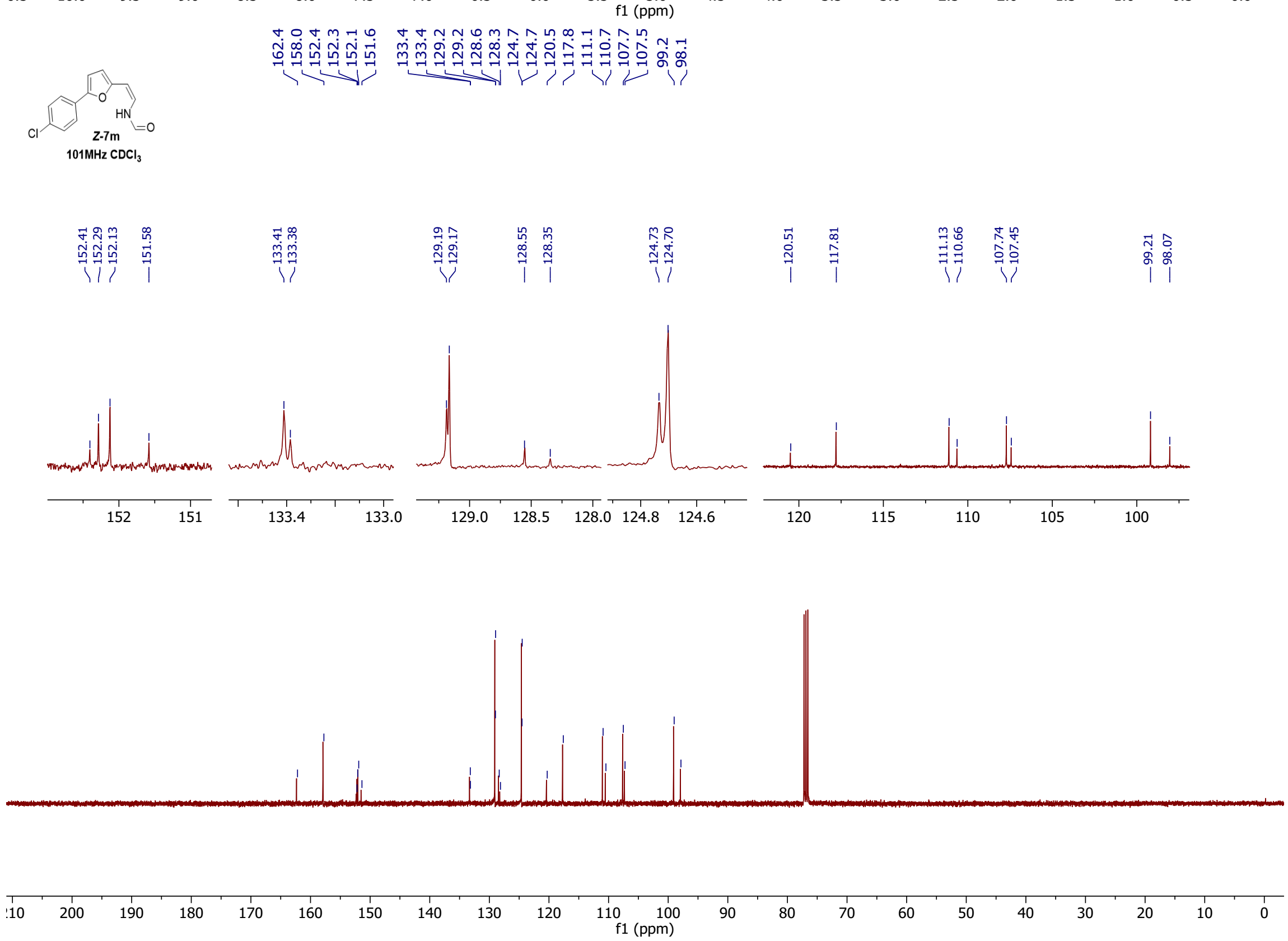

162.4 158.0 152.4 152.3 152.1 151.6 133.4 133.4 129.2 129.2 128.6 128.3 124.7 124.7 120.5 117.8 111.1 110.7 107.7 107.5 99.2 98.1
Cl
HN
O
Z-7m
101MHz CDCl3
152.41 152.29 152.13 151.58 133.41 133.38 129.19 129.17 128.55 128.35 124.73 124.70 120.51 117.81 111.13 110.66 107.74 107.45 99.21 98.07
f1 (ppm)

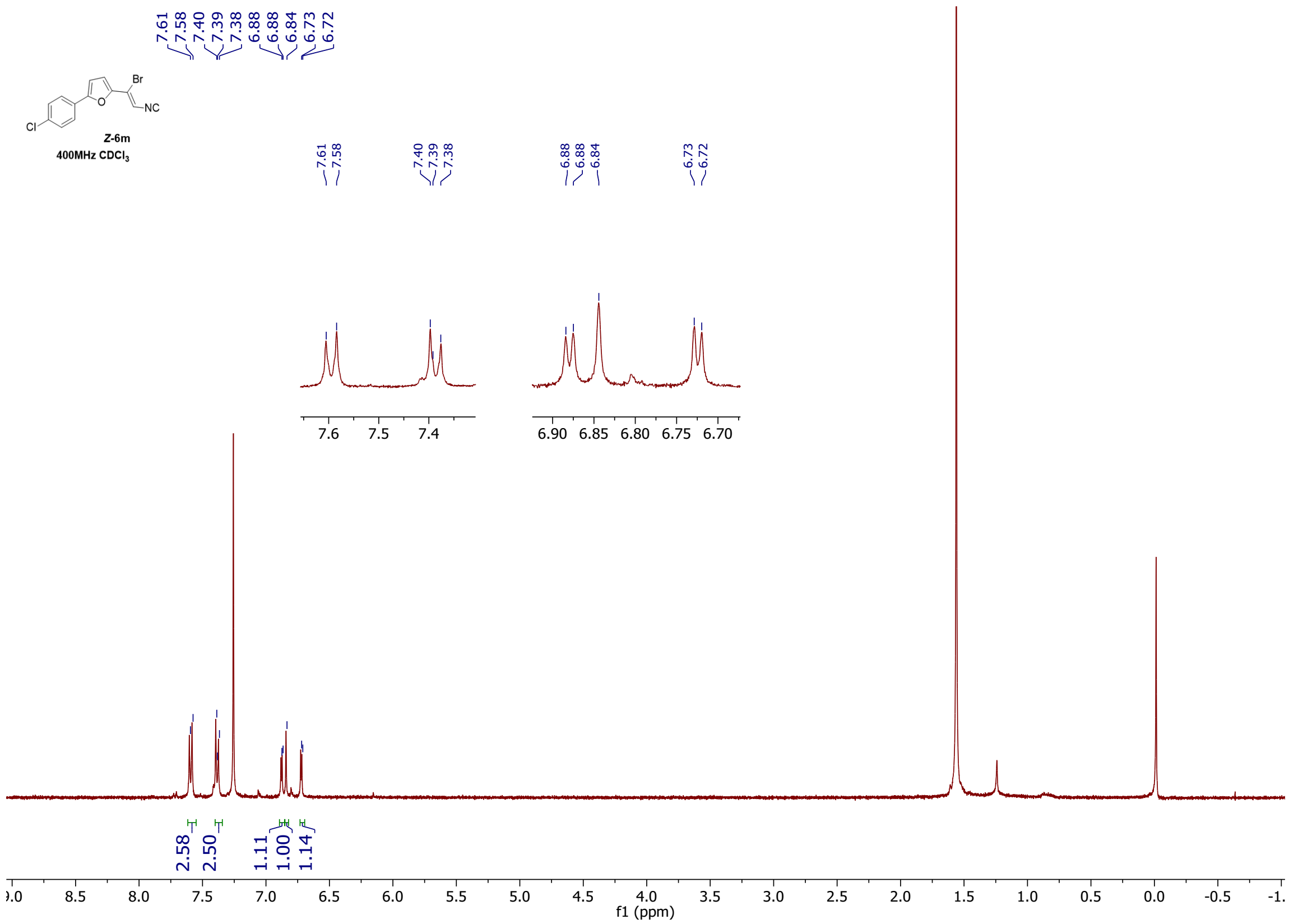
7.61
7.58
7.40
7.39
7.38
6.88
6.88
6.84
6.73
6.72
Br
NC
O
Cl
Z-6m
400MHz CDCl3
2.58
2.50
1.11
1.00
1.14
f1 (ppm)

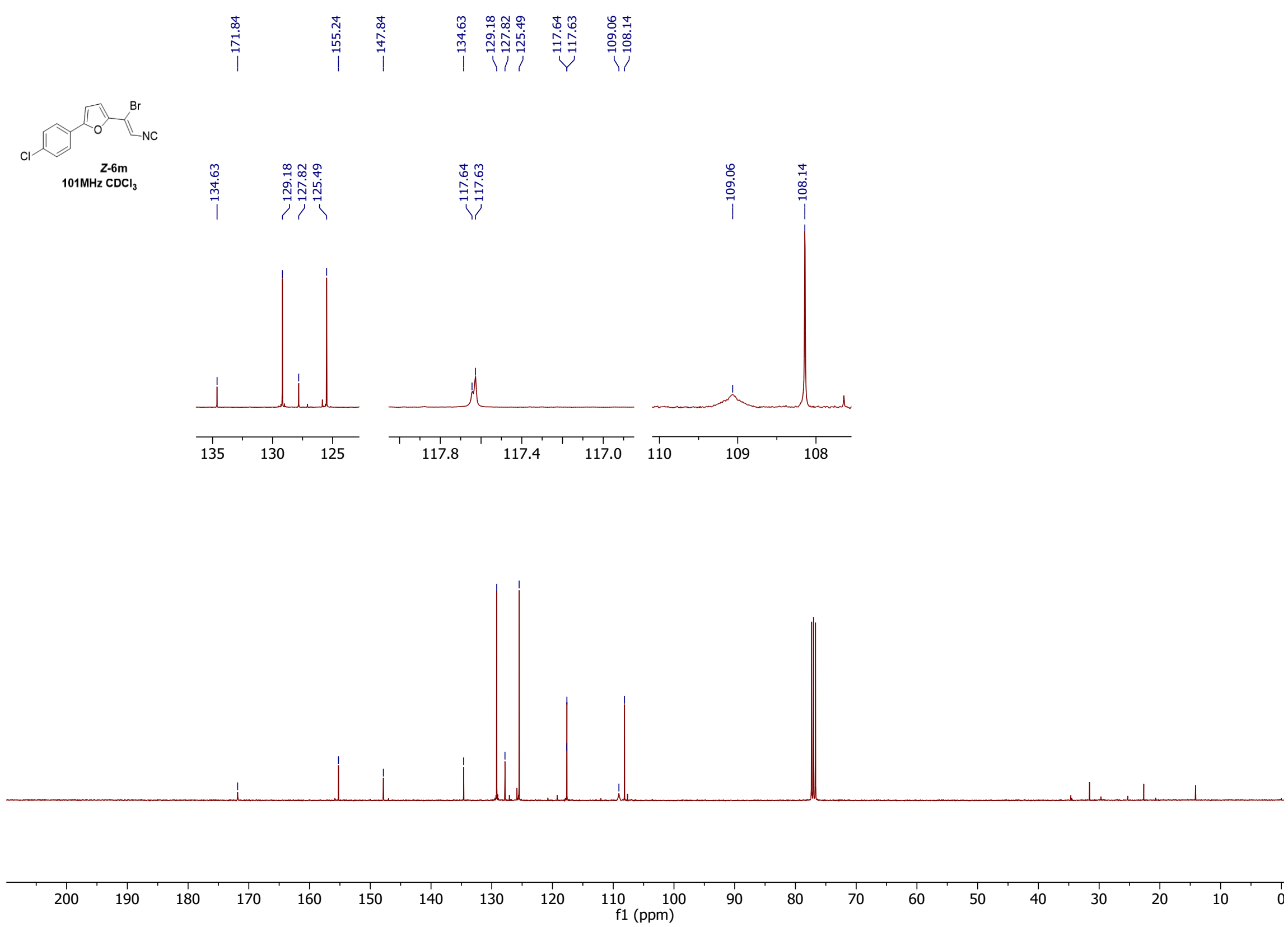
171.84
155.24
147.84
134.63
129.18
127.82
125.49
117.64
117.63
109.06
108.14
Br
NC
O
Cl
Z-6m
101MHz CDCl3
f1 (ppm)

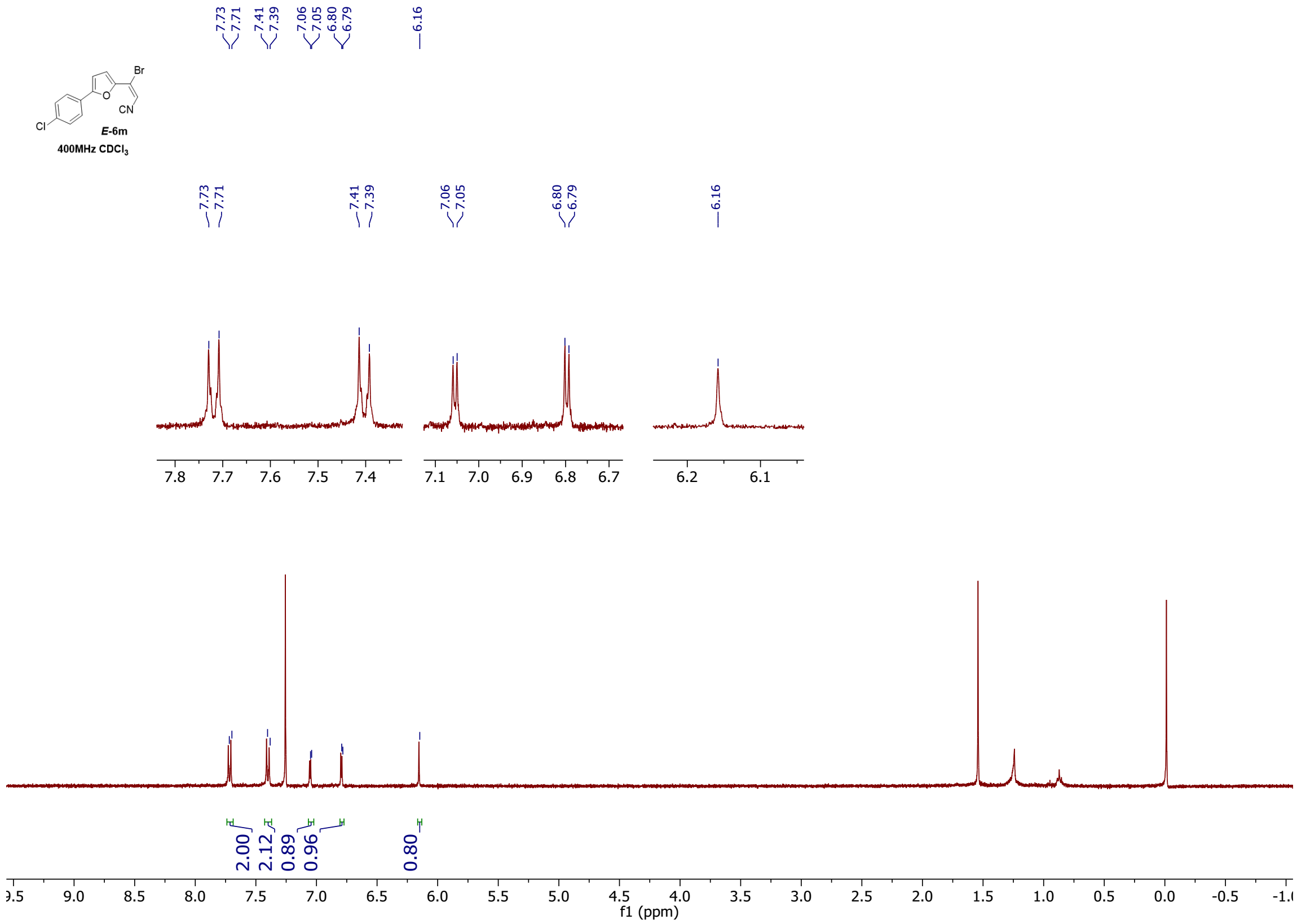

E-6m
400MHz CDCl3
7.73
7.71
7.41
7.39
7.06
7.05
6.80
6.79
6.16
2.00
2.12
0.89
0.96
0.80
f1 (ppm)

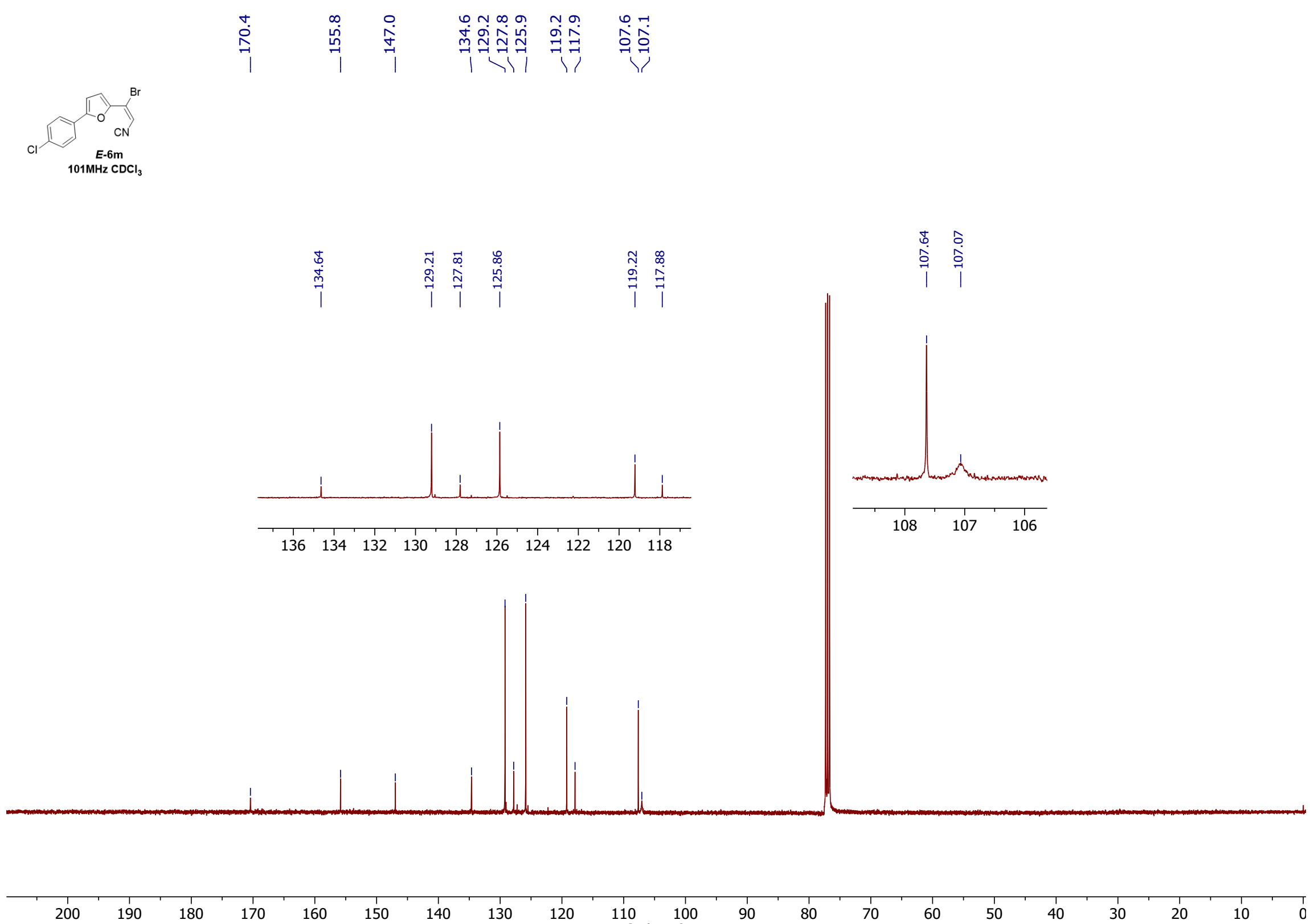

E-6m
101MHz CDCl3
170.4
155.8
147.0
134.6
129.2
127.8
125.9
119.2
117.9
107.6
107.1
134.64
129.21
127.81
125.86
119.22
117.88
107.64
107.07
f1 (ppm)

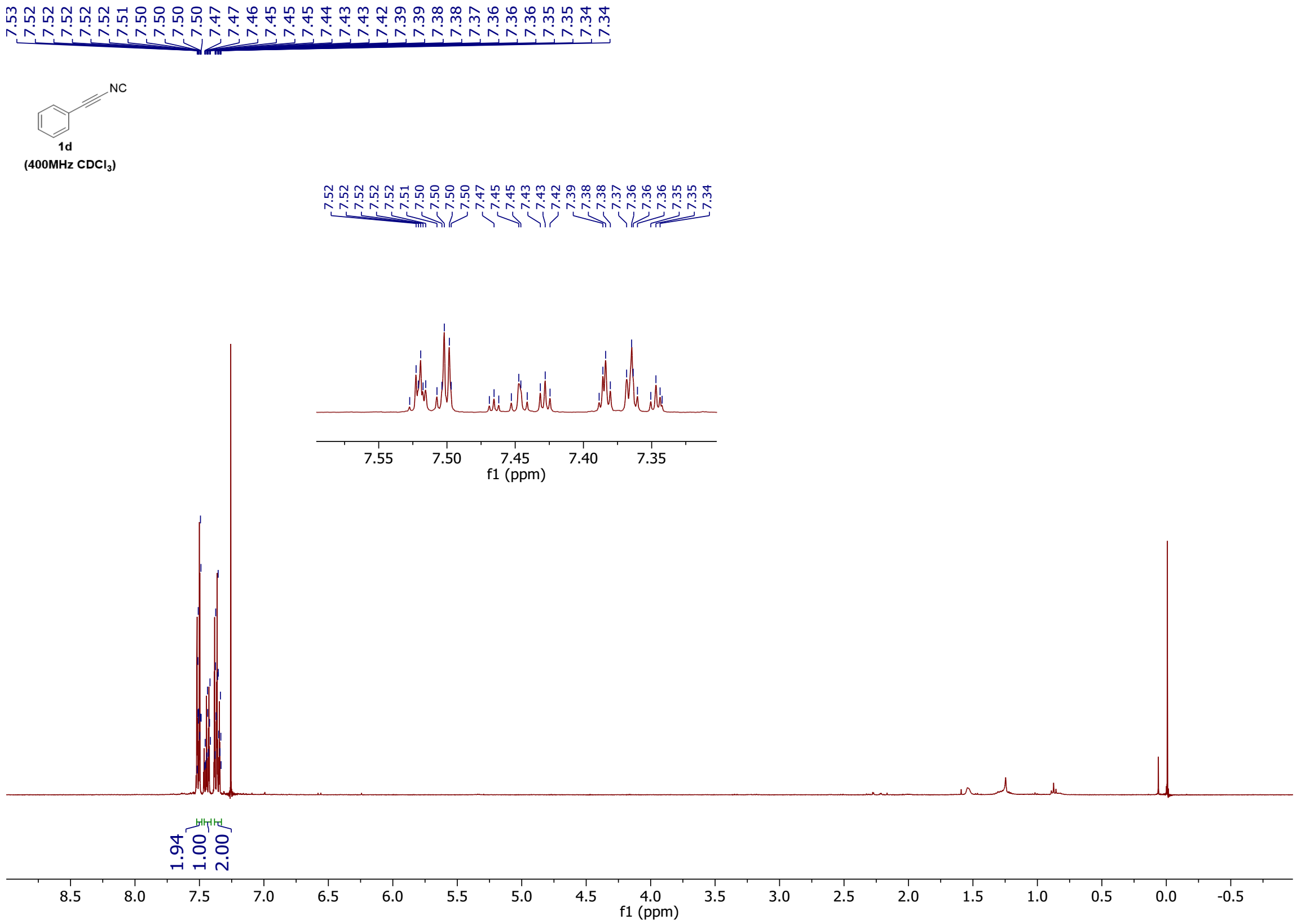
NC
1d
(400MHz CDCl3)
7.53 7.52 7.52 7.52 7.52 7.51 7.50 7.50 7.50 7.50 7.47 7.46 7.45 7.45 7.45 7.44 7.43 7.43 7.42 7.39 7.39 7.38 7.38 7.37 7.36 7.36 7.36 7.35 7.35 7.34 7.34
1.94
1.00
2.00
f1 (ppm)

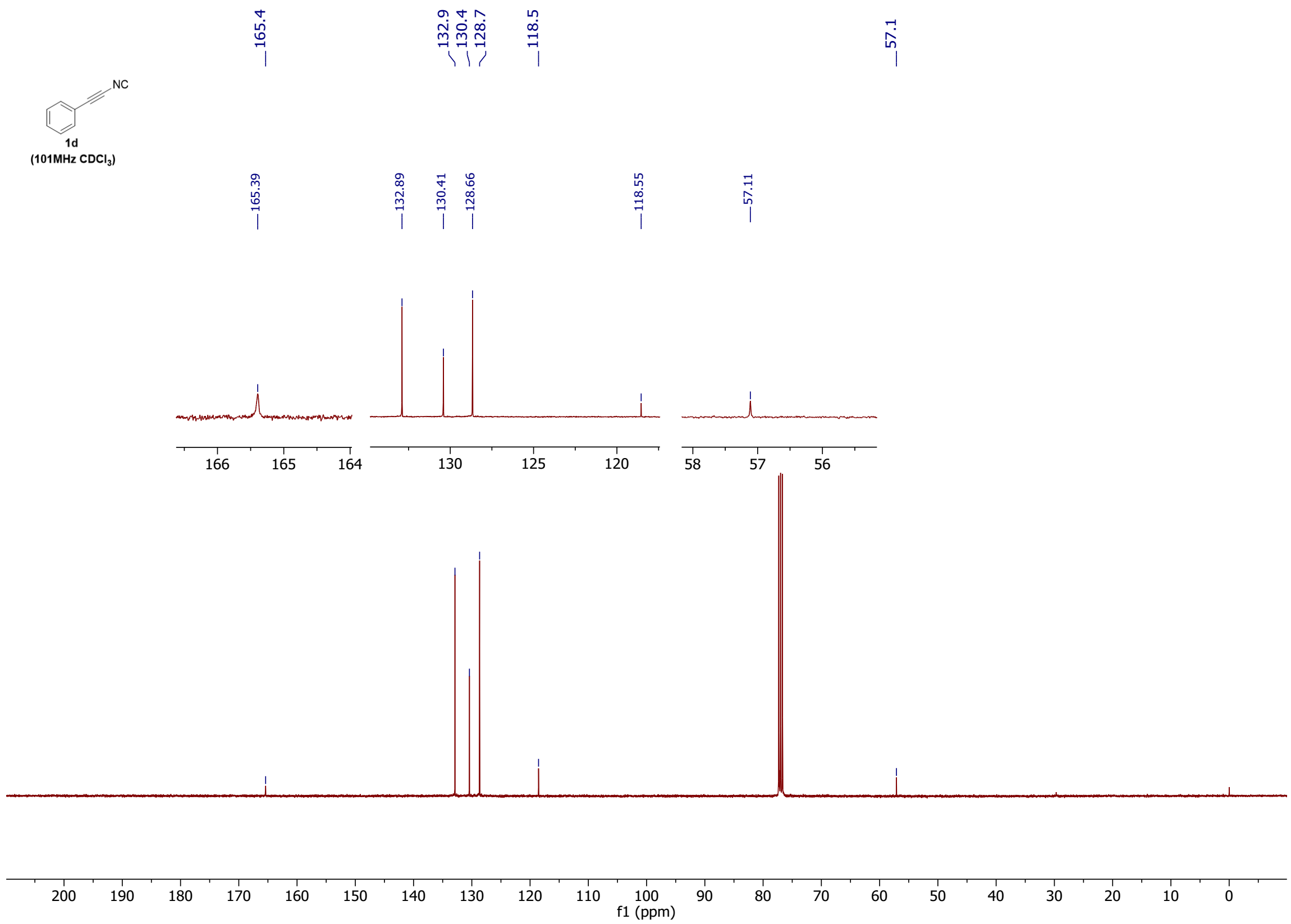
165.4
132.9
130.4
128.7
118.5
57.1
NC
1d
(101MHz CDCl3)
165.39
132.89
130.41
128.66
118.55
57.11
f1 (ppm)

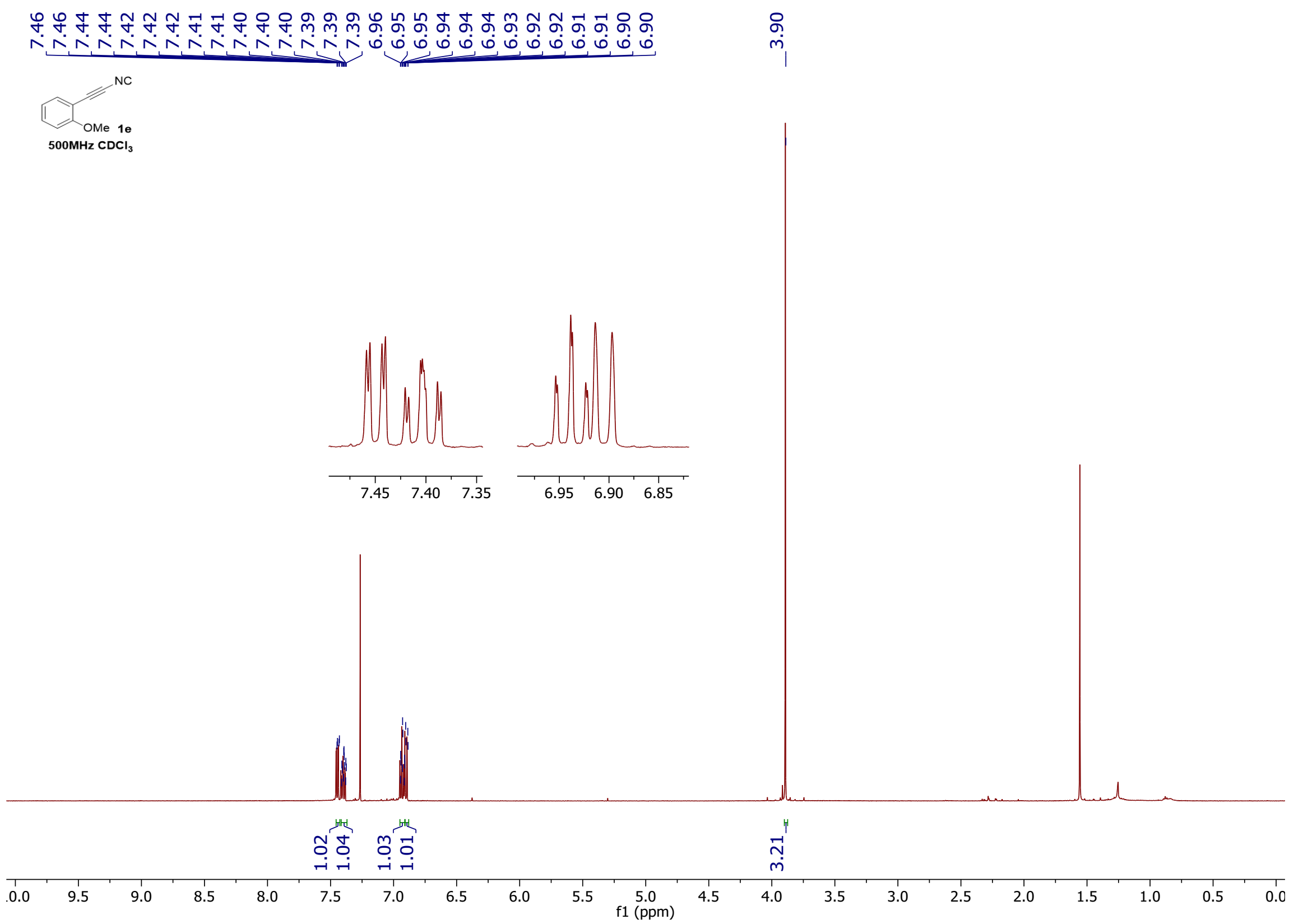
7.46
7.46
7.44
7.44
7.42
7.42
7.42
7.41
7.41
7.40
7.40
7.40
7.39
7.39
7.39
6.96
6.95
6.95
6.94
6.94
6.94
6.93
6.92
6.92
6.91
6.91
6.90
6.90
3.90
NC
OMe 1e
500MHz CDCl3
1.02
1.04
1.03
1.01
3.21
f1 (ppm)

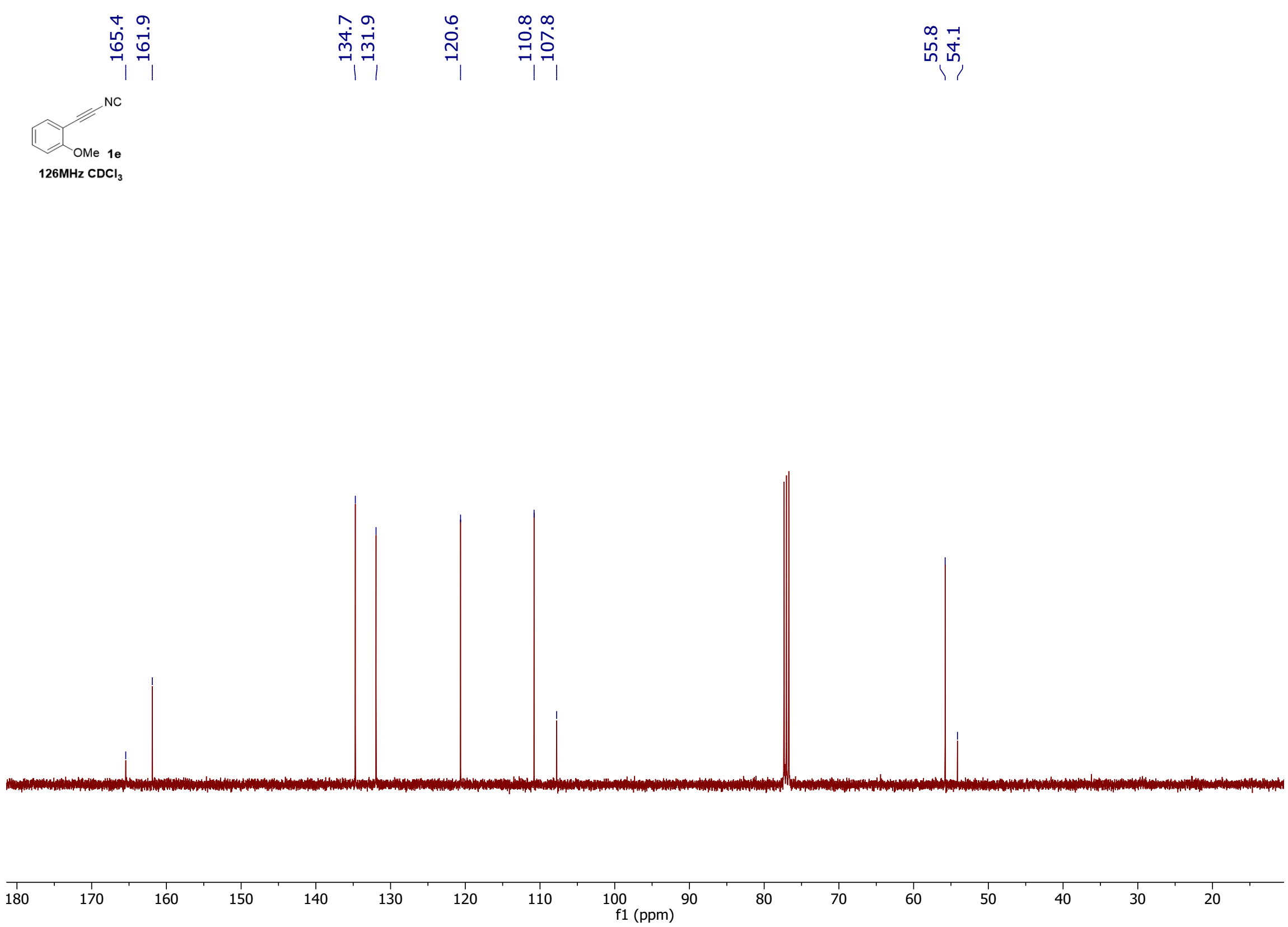
165.4
161.9
134.7
131.9
120.6
110.8
107.8
55.8
54.1
NC
OMe 1e
126MHz CDCl3
f1 (ppm)

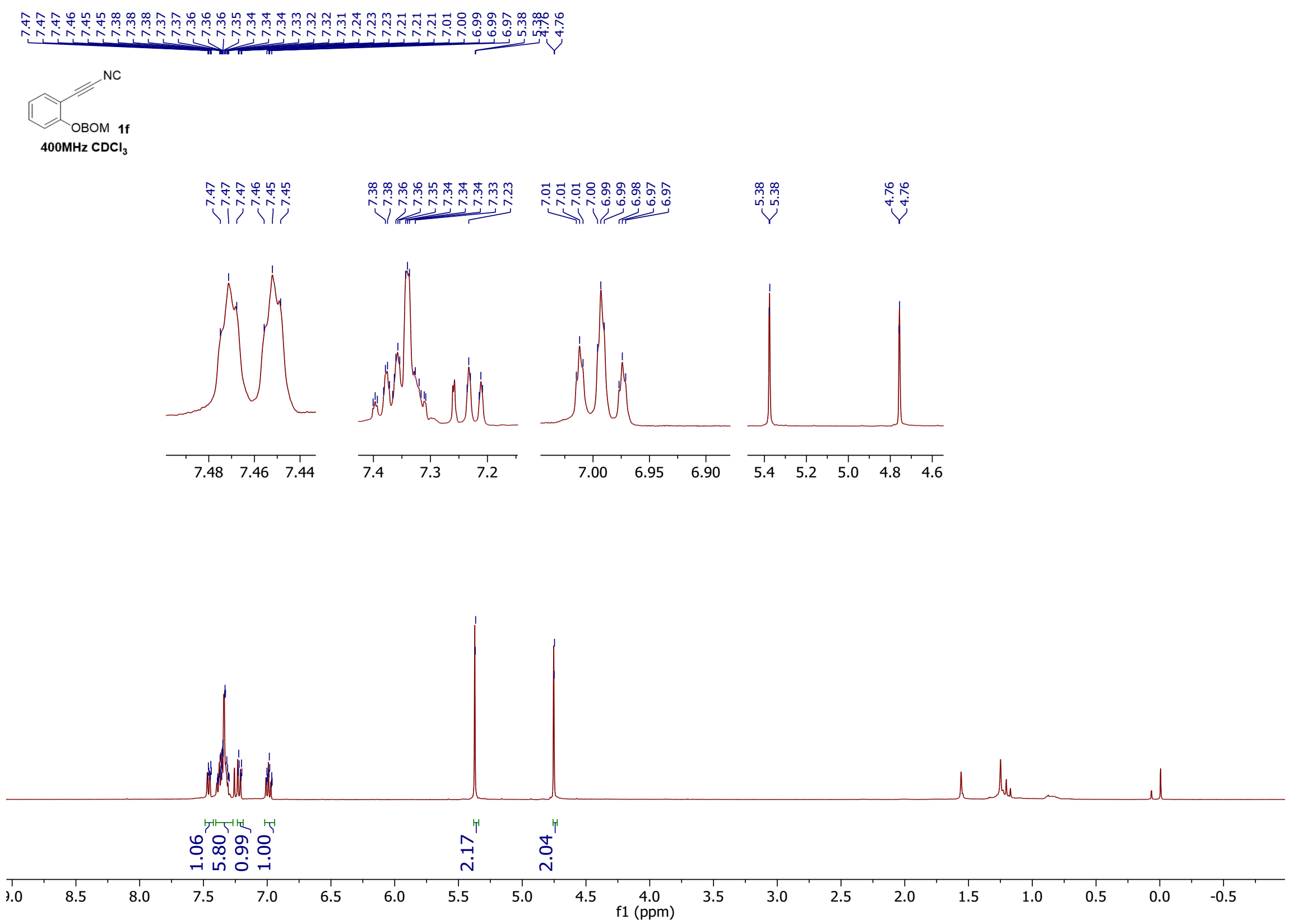
NC
OBOM 1f
400MHz CDCl3
1.06
5.80
0.99
1.00
2.17
2.04
f1 (ppm)

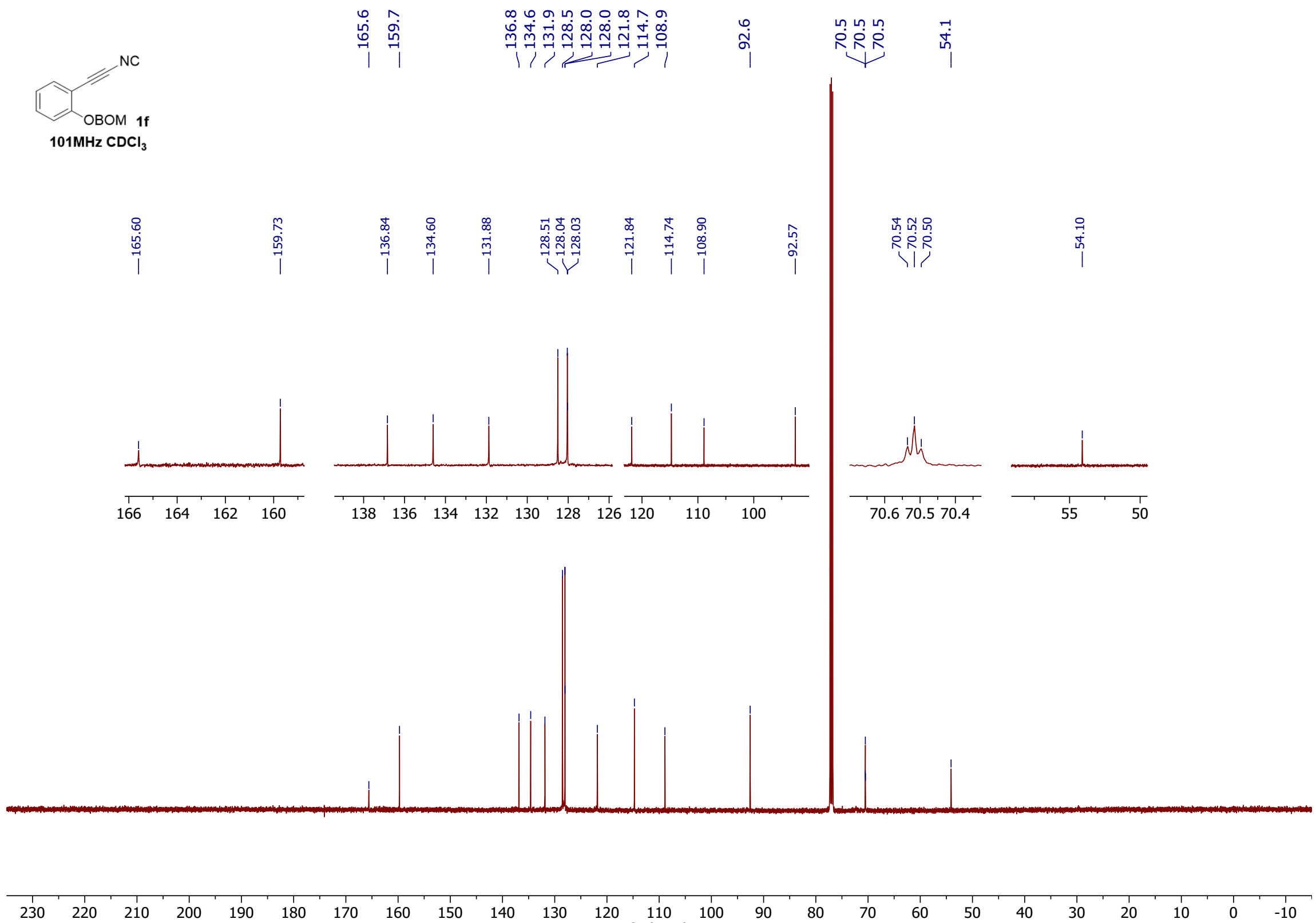
NC
OBOM 1f
101MHz CDCl3
165.6
159.7
136.8
134.6
131.9
128.5
128.0
128.0
121.8
114.7
108.9
92.6
70.5
70.5
70.5
54.1
f1 (ppm)

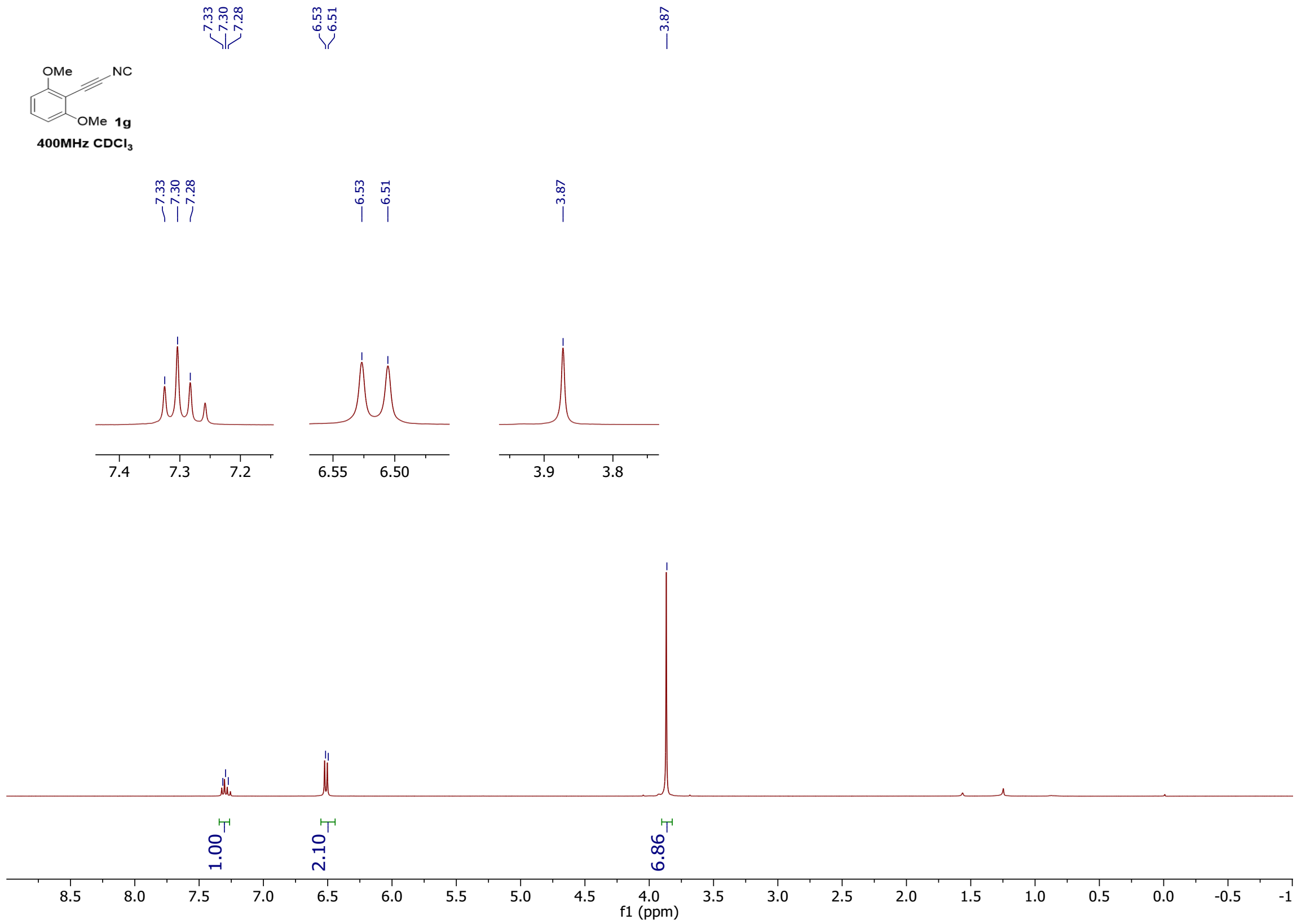
OMe
NC
OMe 1g
400MHz CDCl3
7.33
7.30
7.28
6.53
6.51
3.87
1.00
2.10
6.86
f1 (ppm)

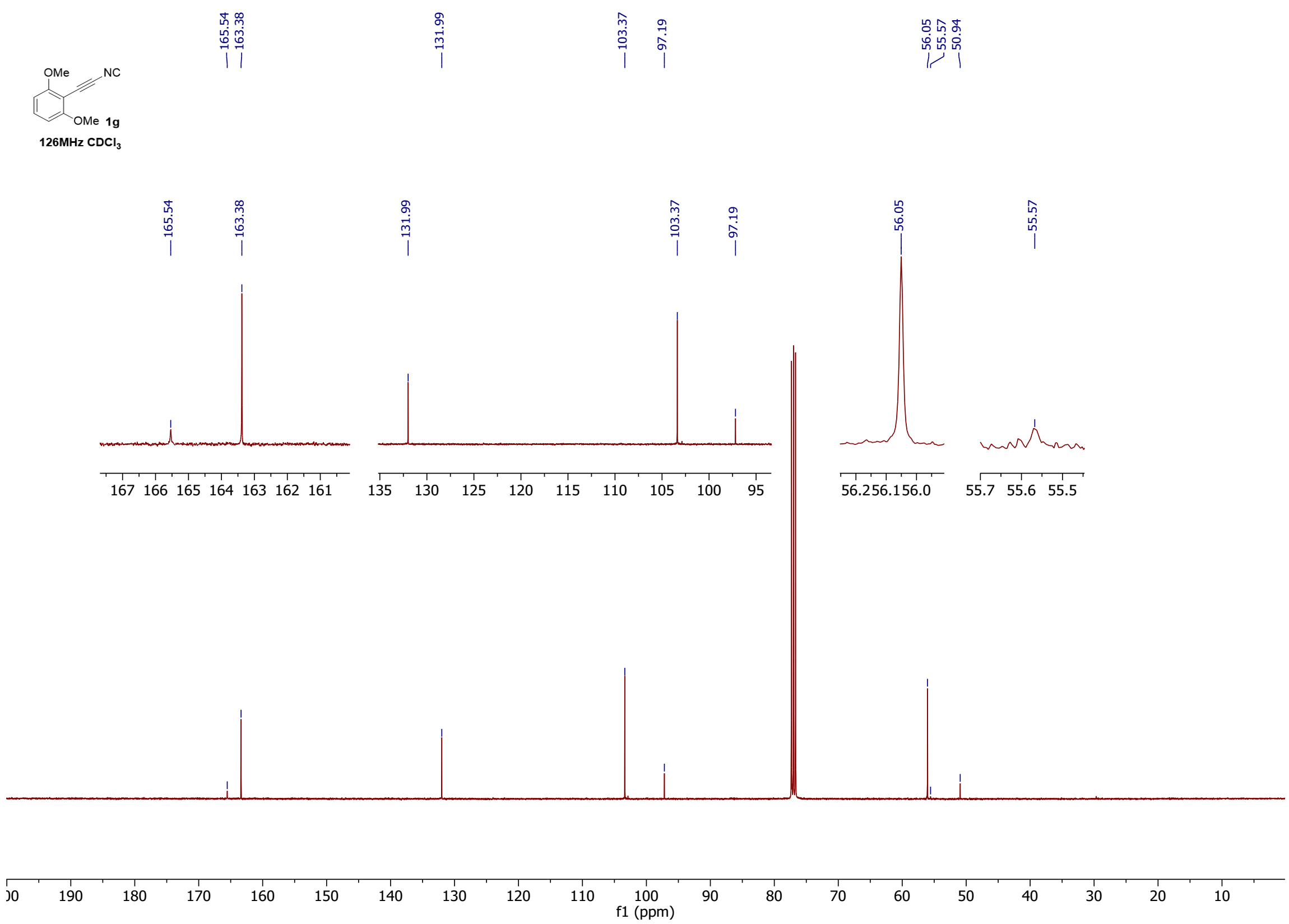
OMe
NC
OMe 1g
126MHz CDCl3
165.54
163.38
131.99
103.37
97.19
56.05
55.57
50.94
f1 (ppm)

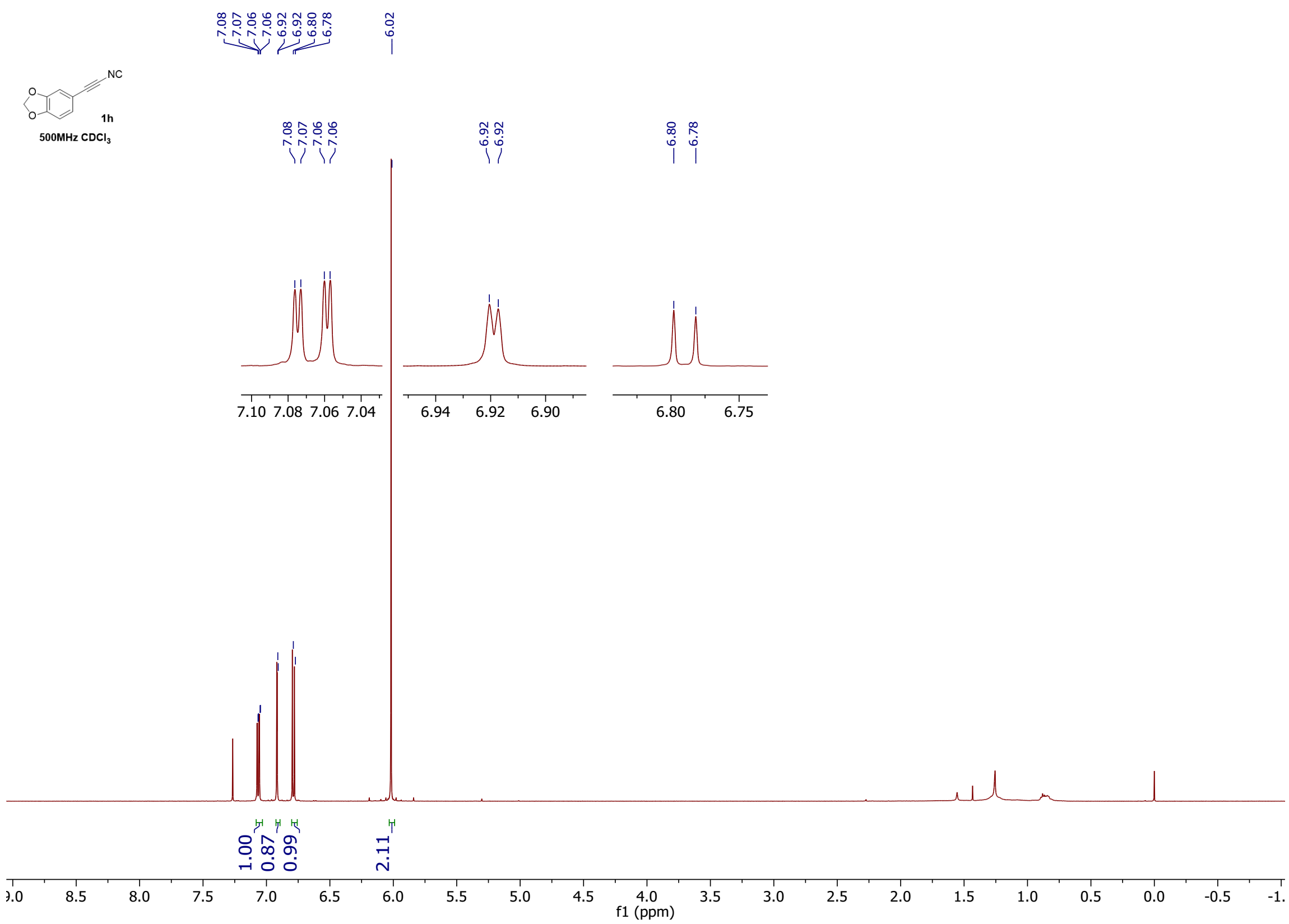
NC
1h
500MHz CDCl$_3$
7.08
7.07
7.06
7.06
6.92
6.92
6.80
6.78
6.02
1.00
0.87
0.99
2.11
f1 (ppm)

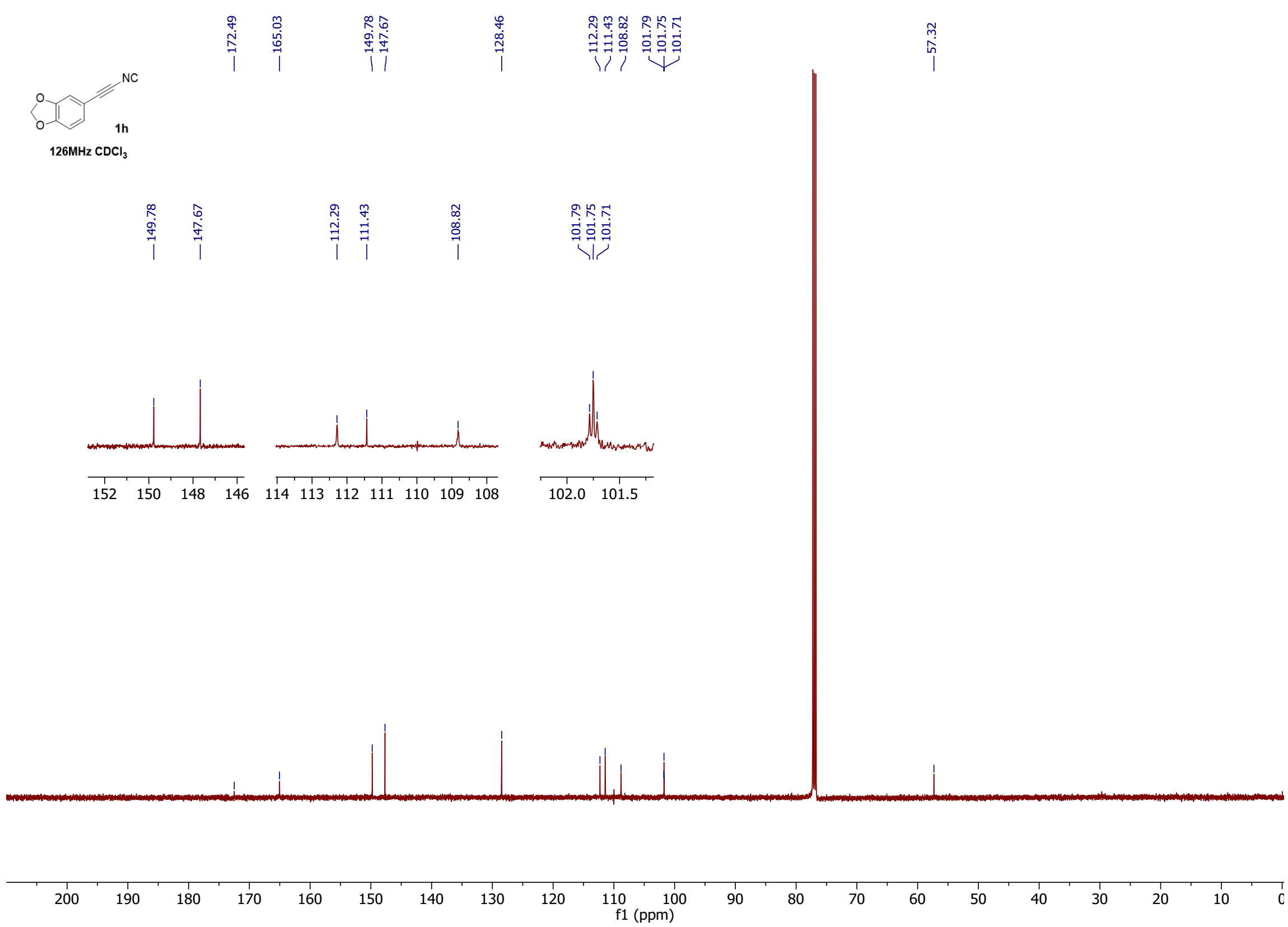
NC
1h
126MHz CDCl$_3$
172.49
165.03
149.78
147.67
128.46
112.29
111.43
108.82
101.79
101.75
101.71
57.32
f1 (ppm)

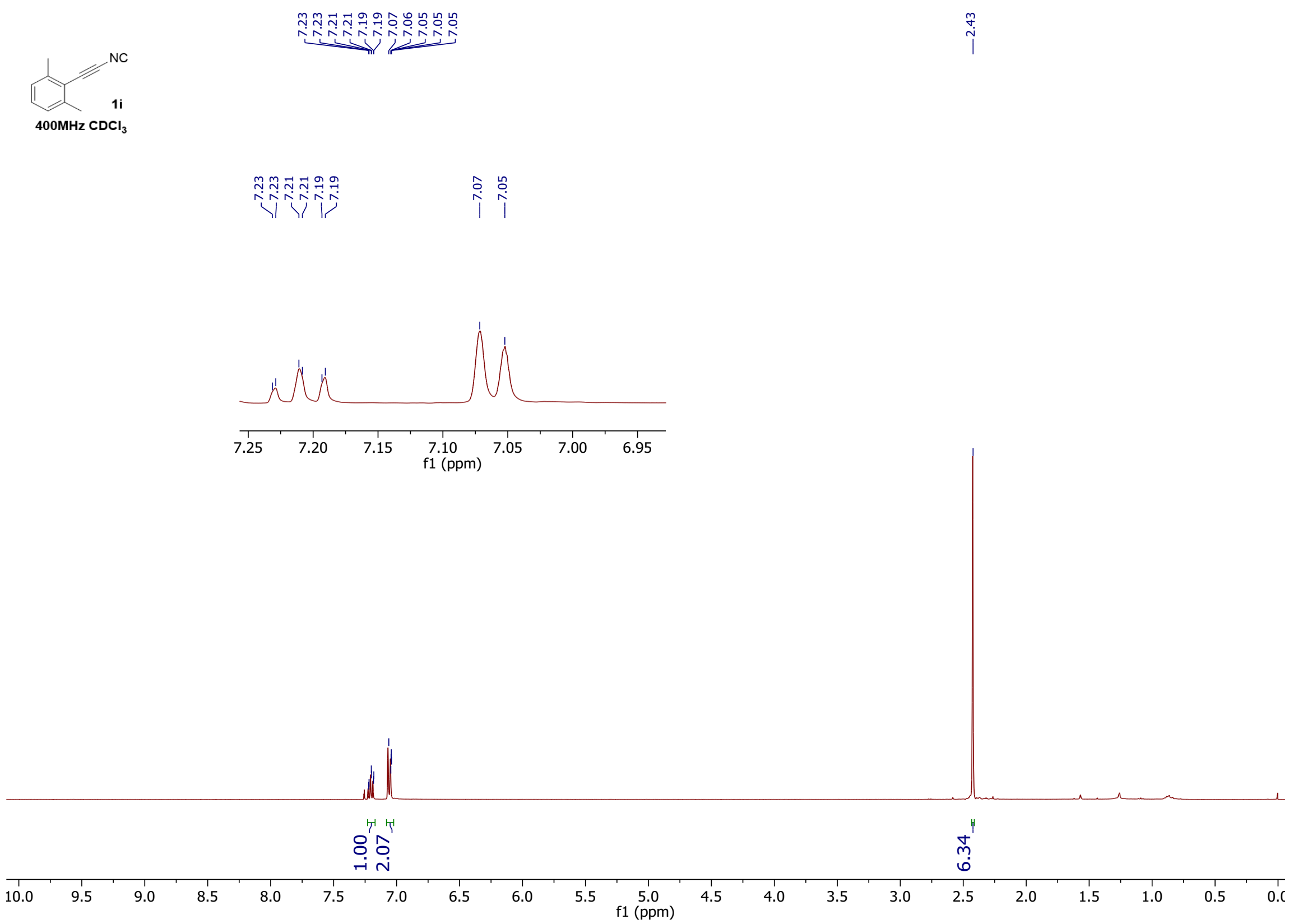

NC
1i
400MHz $CDCl_3$
7.23
7.23
7.21
7.21
7.19
7.19
7.07
7.06
7.05
7.05
7.05
2.43
7.07
7.05
1.00
2.07
6.34
f1 (ppm)

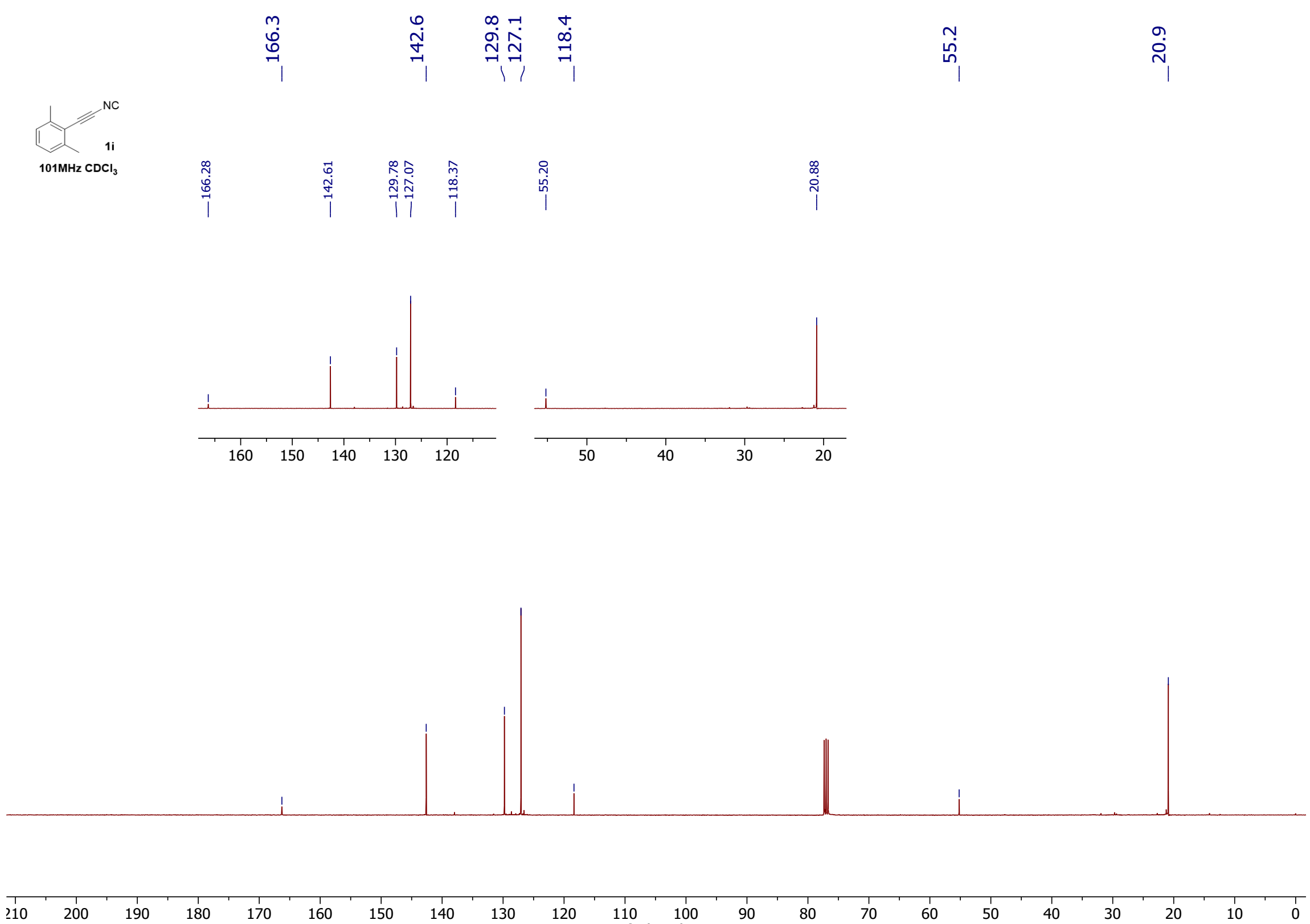

NC
1i
101MHz $CDCl_3$
166.3
142.6
129.8
127.1
118.4
55.2
20.9
166.28
142.61
129.78
127.07
118.37
55.20
20.88
f1 (ppm)

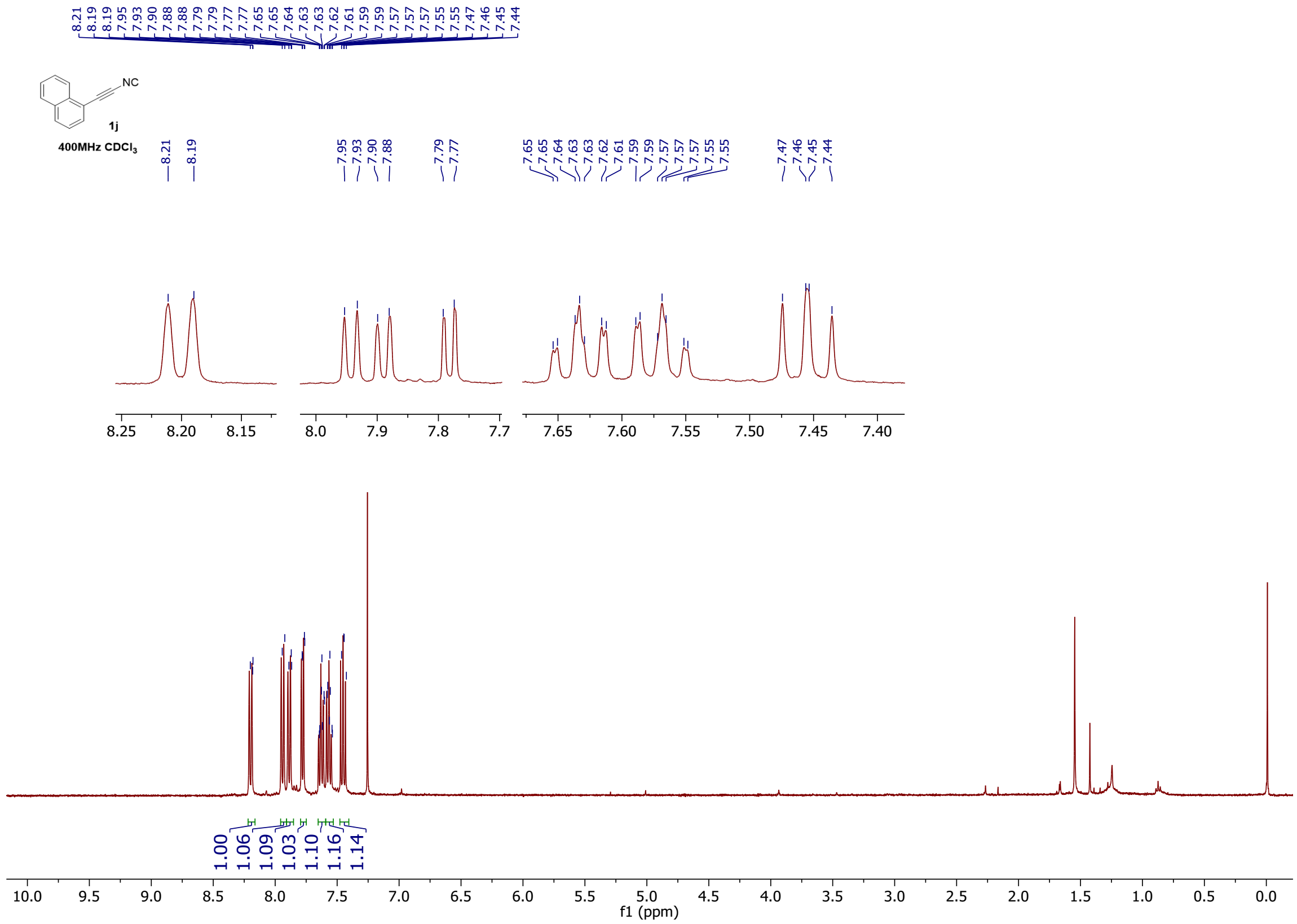
8.21 8.19 8.19 7.95 7.93 7.90 7.88 7.88 7.79 7.79 7.77 7.77 7.65 7.65 7.64 7.63 7.63 7.62 7.61 7.59 7.59 7.57 7.57 7.57 7.55 7.55 7.47 7.46 7.45 7.44
NC
1j
400MHz CDCl3
1.00
1.06
1.09
1.03
1.10
1.16
1.14
f1 (ppm)

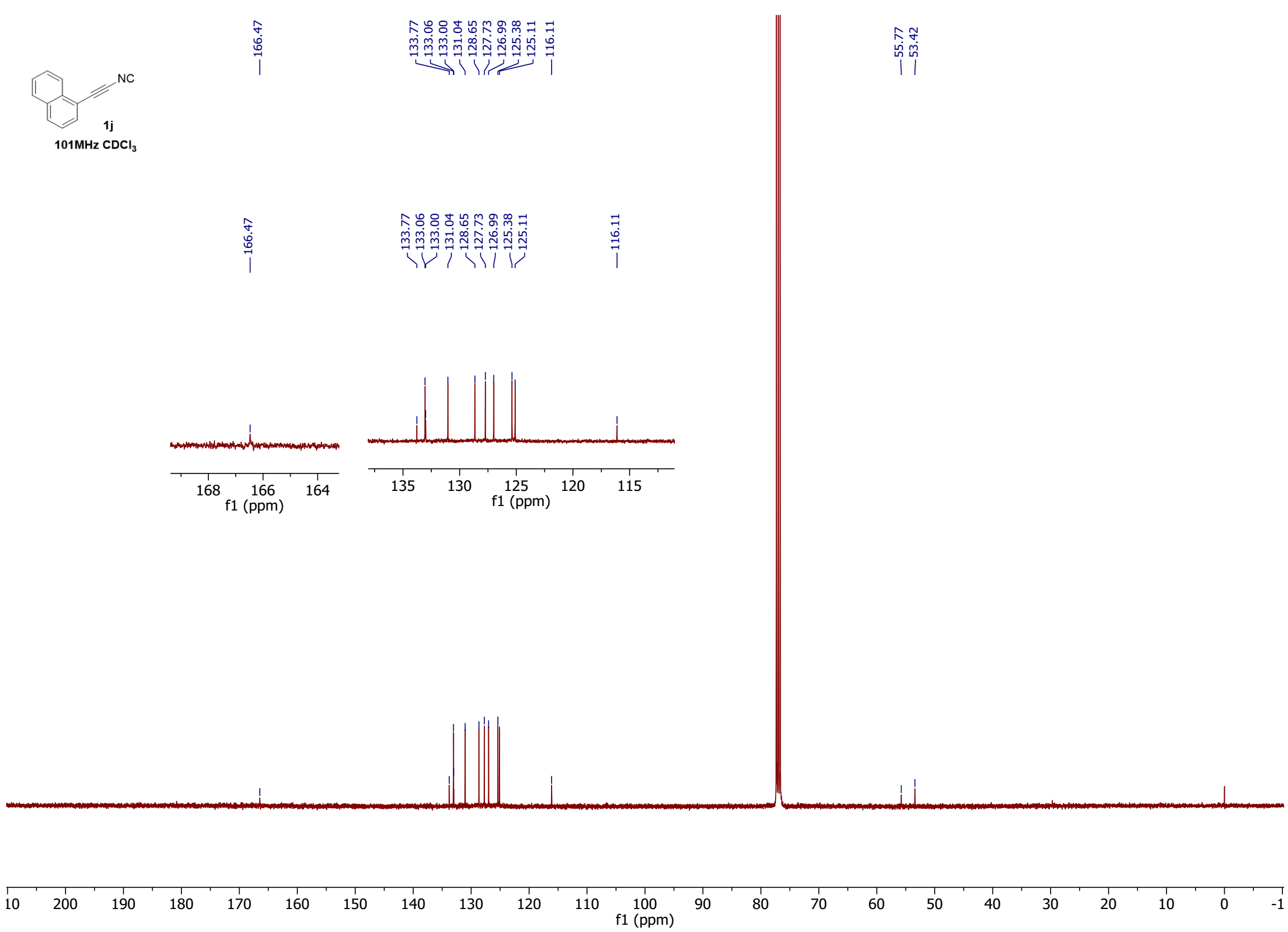
166.47
133.77 133.06 133.00 131.04 128.65 127.73 126.99 125.38 125.11
116.11
55.77 53.42
NC
1j
101MHz CDCl3
f1 (ppm)

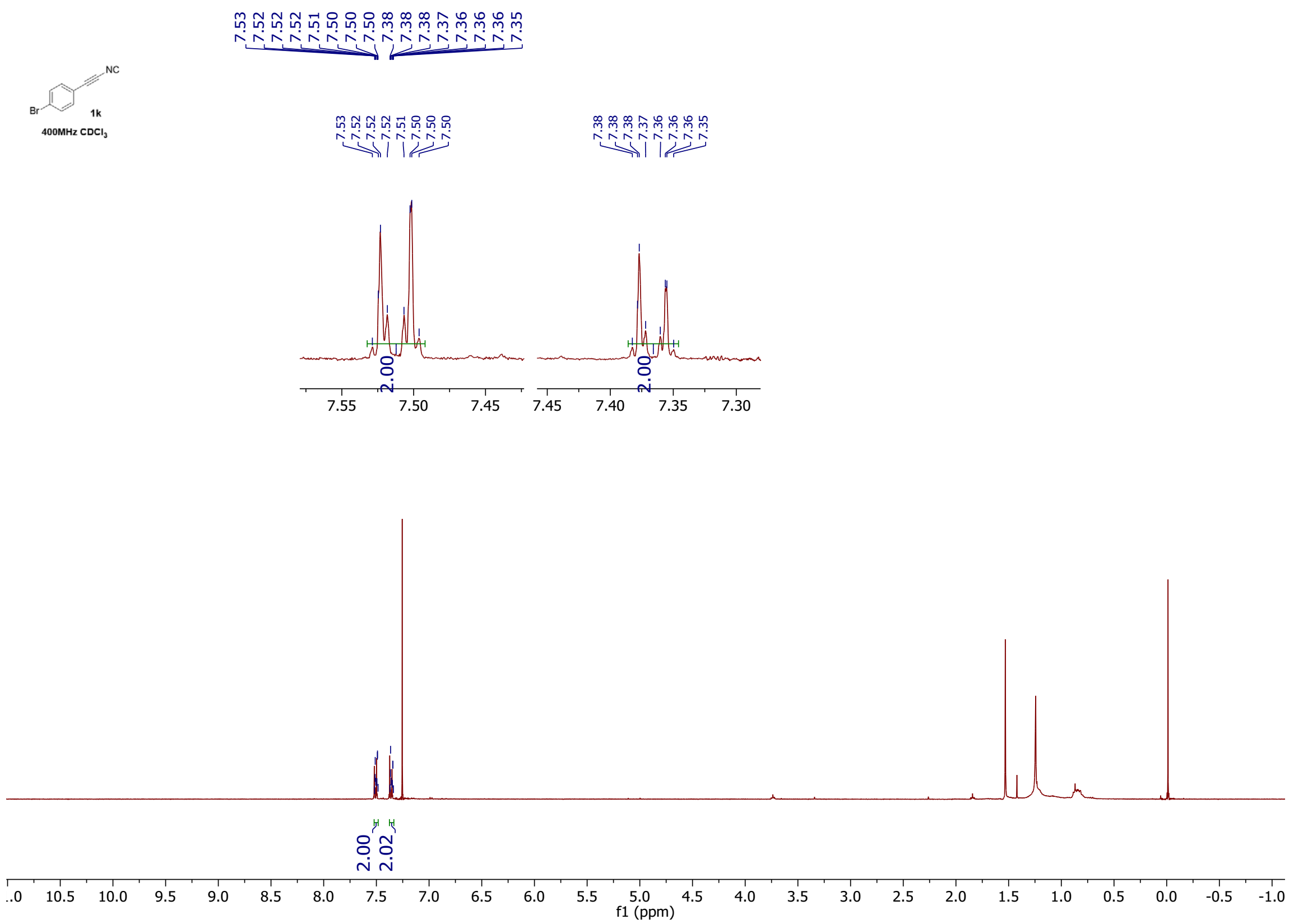
NC
Br
1k
400MHz CDCl3
7.53 7.52 7.52 7.52 7.51 7.50 7.50 7.50 7.38 7.38 7.38 7.37 7.36 7.36 7.36 7.35
2.00
2.00
2.02
f1 (ppm)

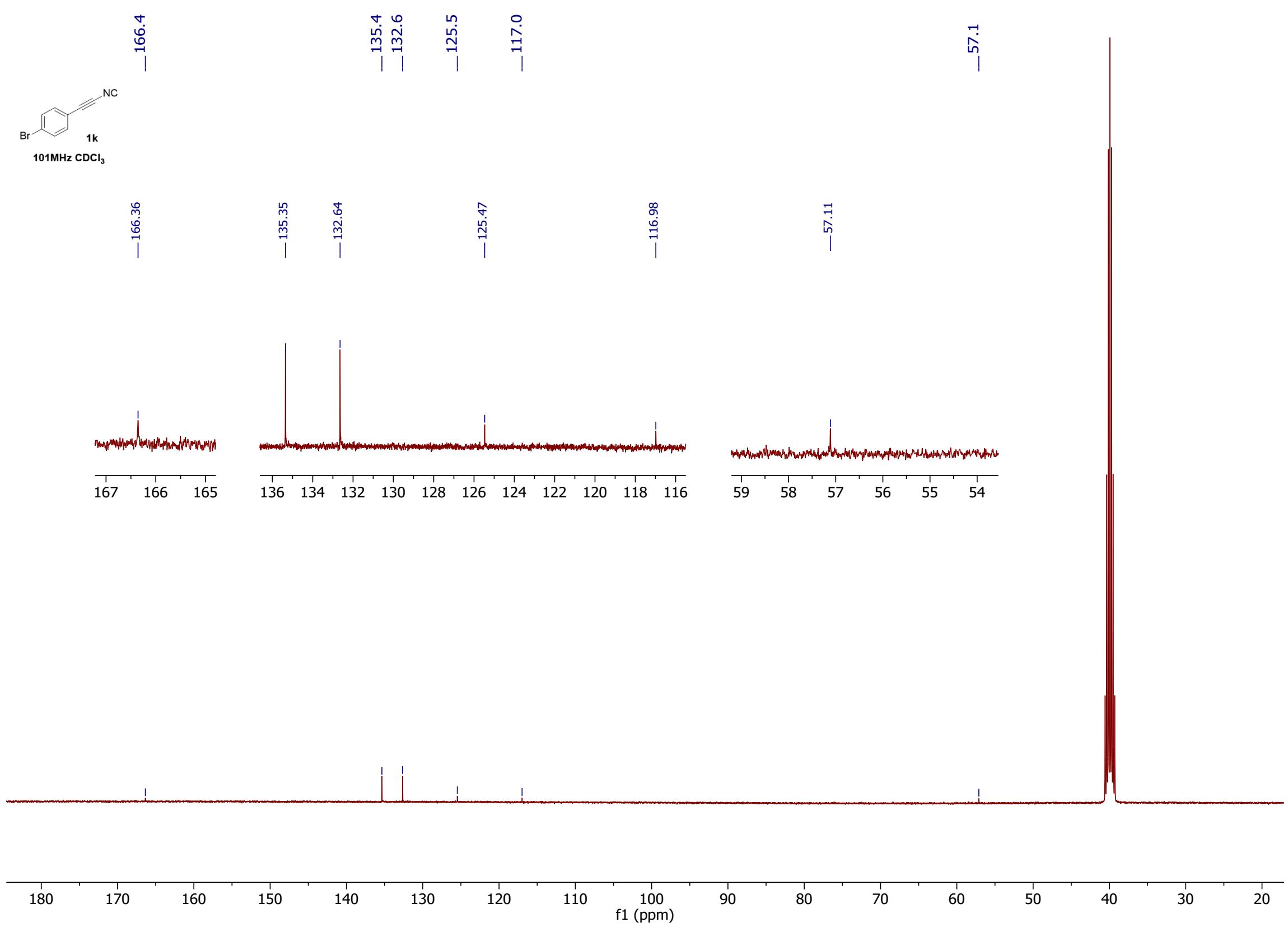
166.4
135.4
132.6
125.5
117.0
57.1
NC
Br
1k
101MHz CDCl3
166.36
135.35
132.64
125.47
116.98
57.11
f1 (ppm)

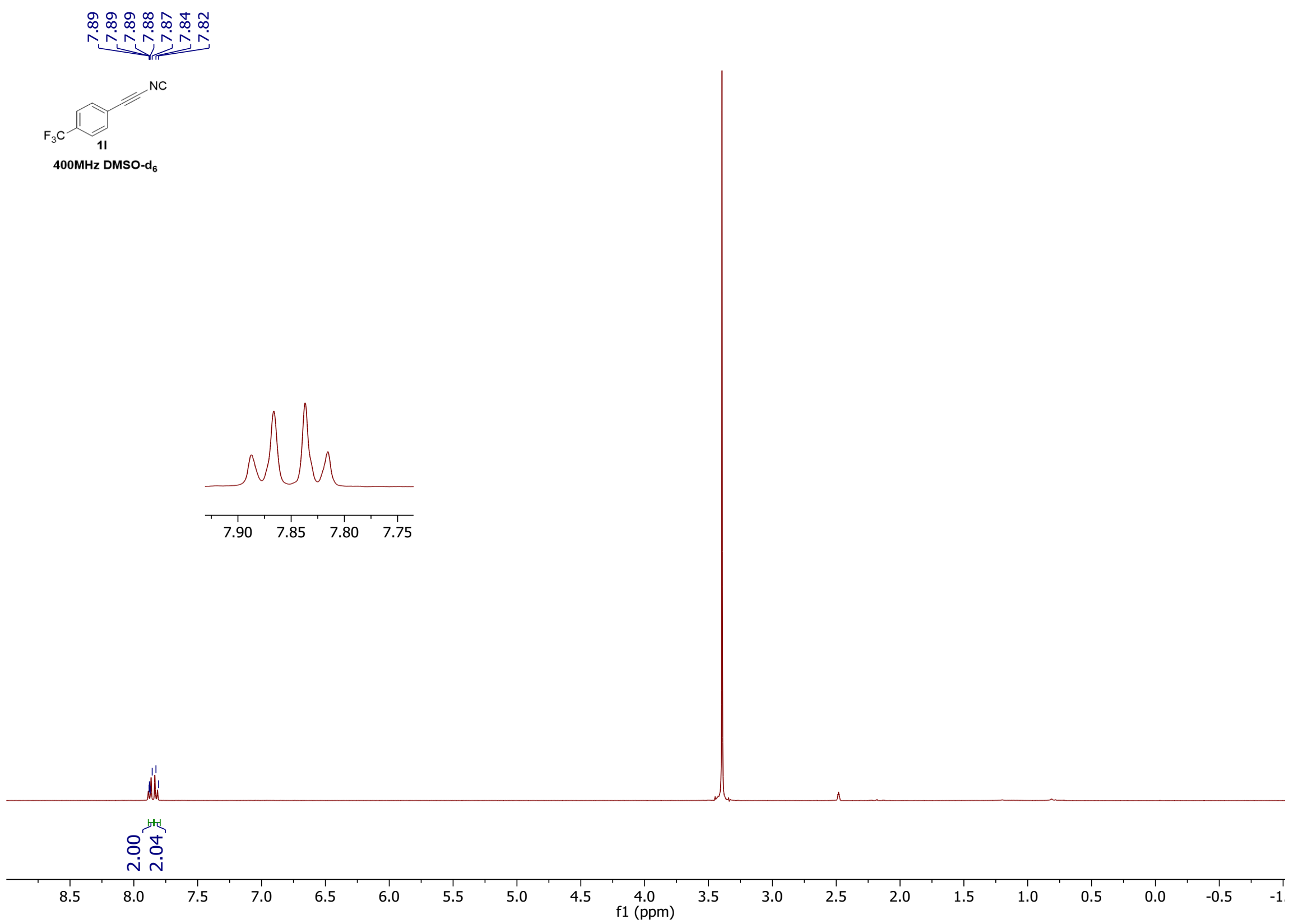
7.89
7.89
7.89
7.88
7.87
7.84
7.82
NC
F3C
1l
400MHz DMSO-d6
2.00
2.04
f1 (ppm)

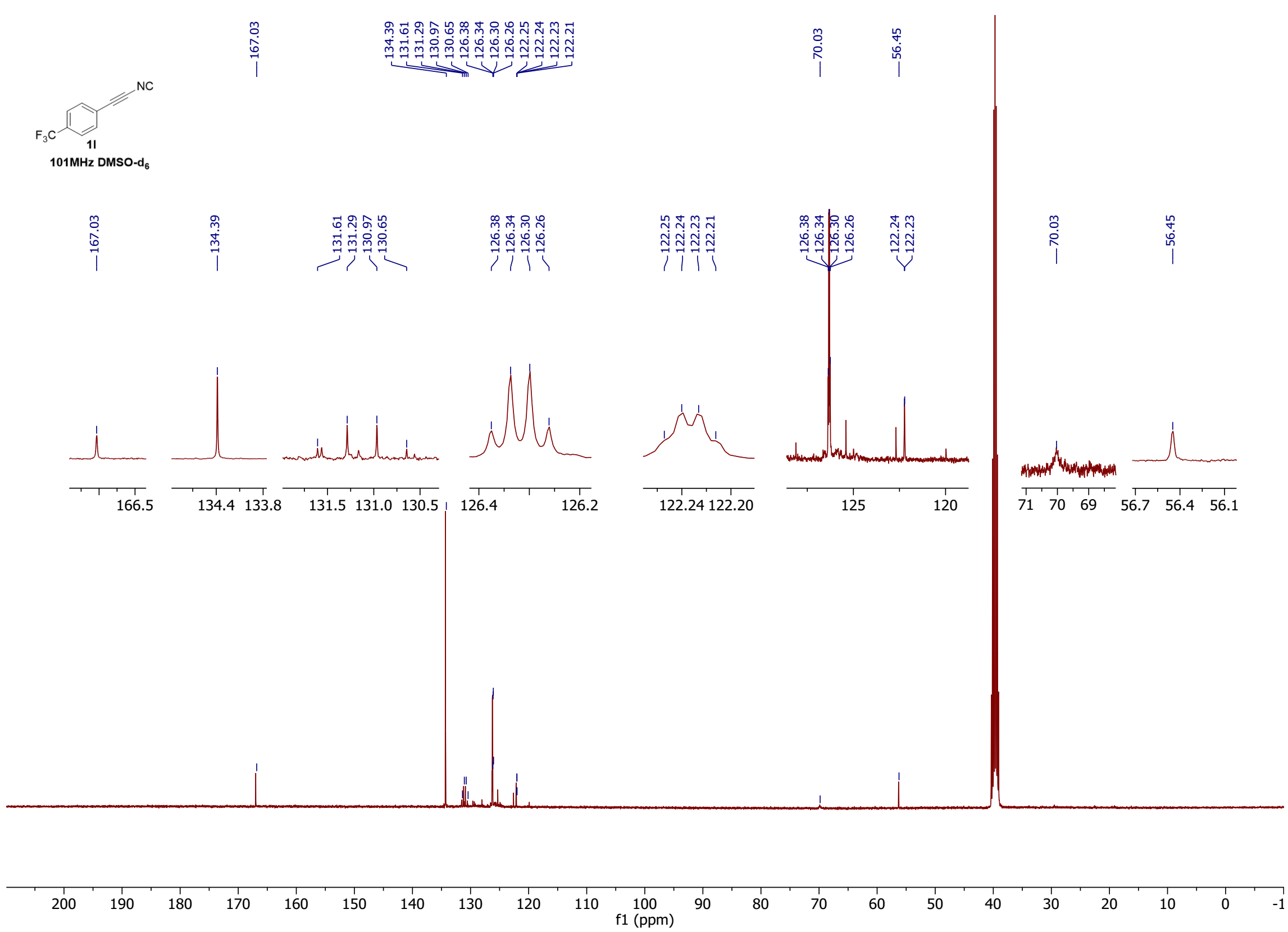
167.03
134.39
131.61
131.29
130.97
130.65
126.38
126.34
126.30
126.26
122.25
122.24
122.23
122.21
70.03
56.45
NC
F3C
1l
101MHz DMSO-d6
f1 (ppm)

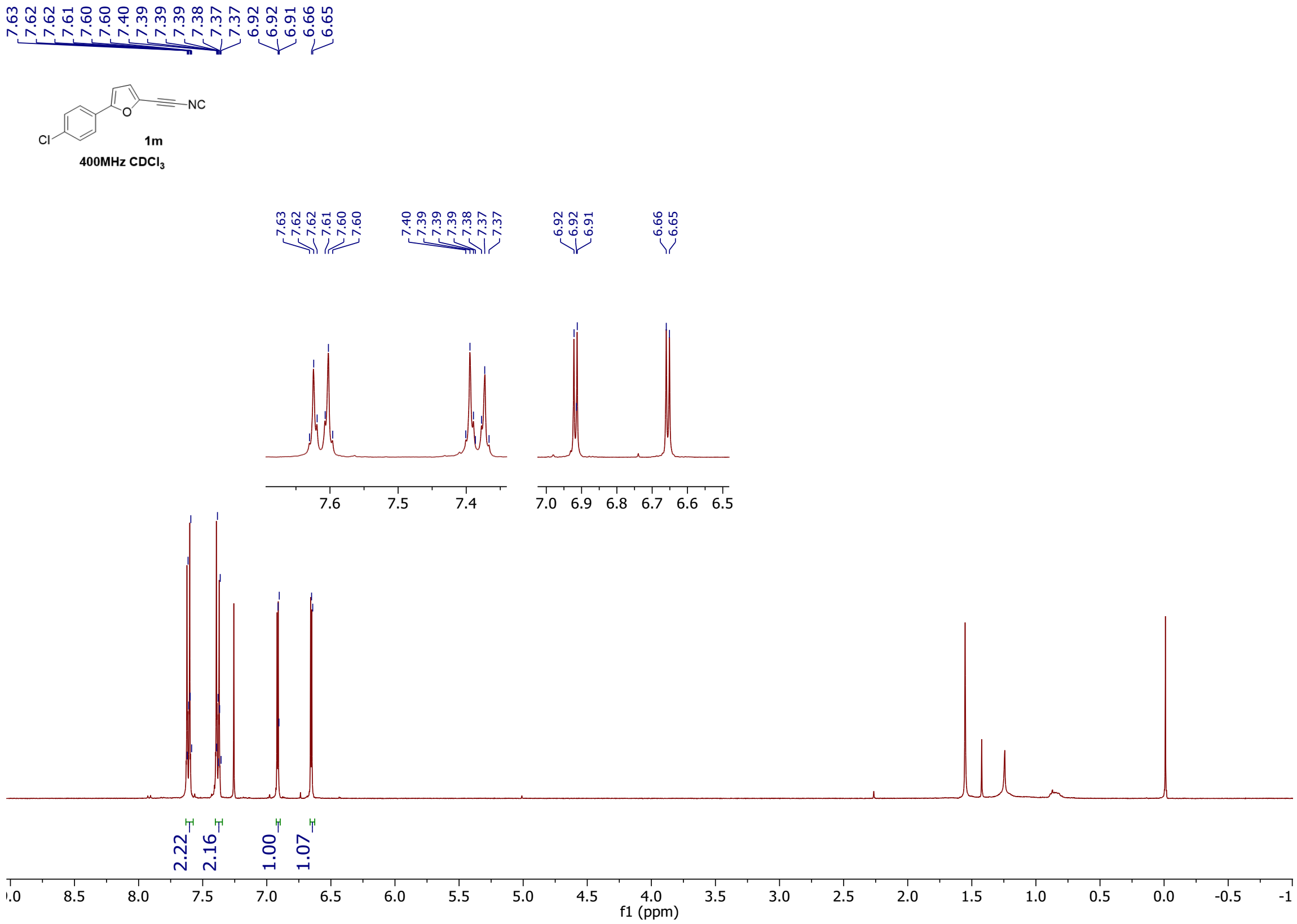
7.63
7.62
7.62
7.61
7.60
7.60
7.40
7.39
7.39
7.39
7.38
7.37
7.37
6.92
6.92
6.91
6.66
6.65
NC
Cl
O
1m
400MHz $CDCl_3$
2.22
2.16
1.00
1.07
f1 (ppm)

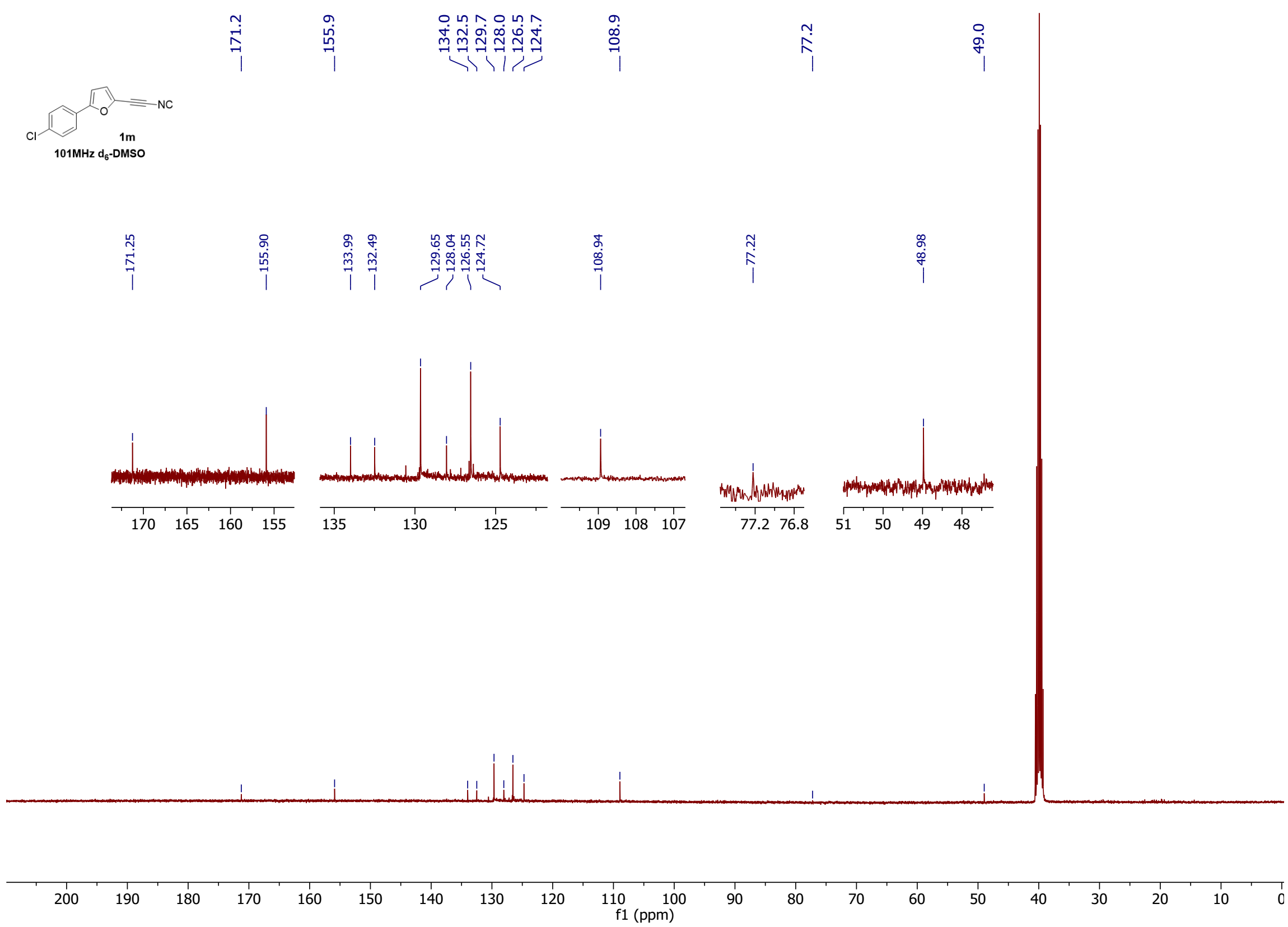
171.2
155.9
134.0
132.5
129.7
128.0
126.5
124.7
108.9
77.2
49.0
NC
Cl
O
1m
101MHz d_6-DMSO
171.25
155.90
133.99
132.49
129.65
128.04
126.55
124.72
108.94
77.22
48.98
f1 (ppm)

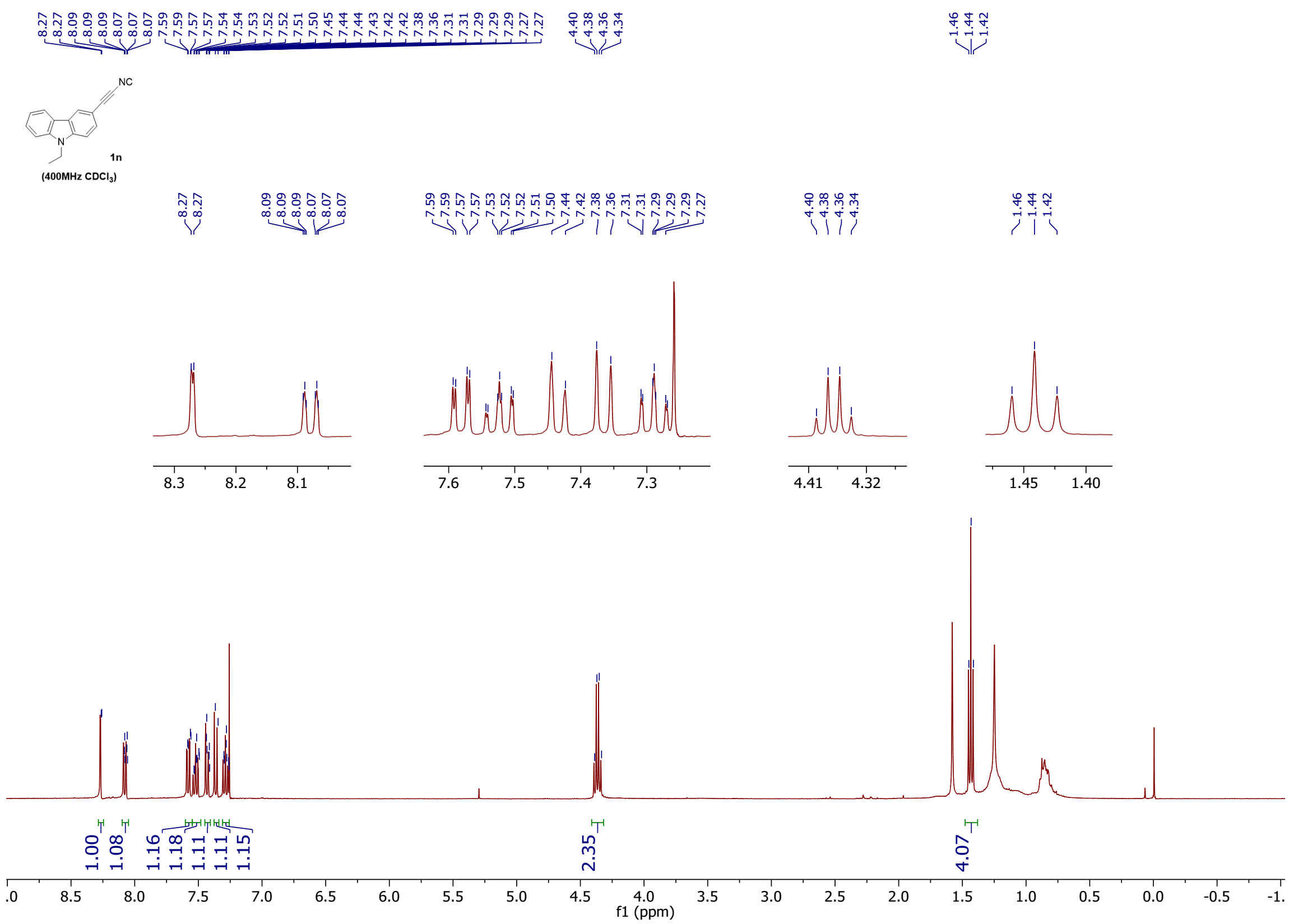
NC
1n
(400MHz CDCl3)
1.00
1.08
1.16
1.18
1.11
1.11
1.15
2.35
4.07
f1 (ppm)

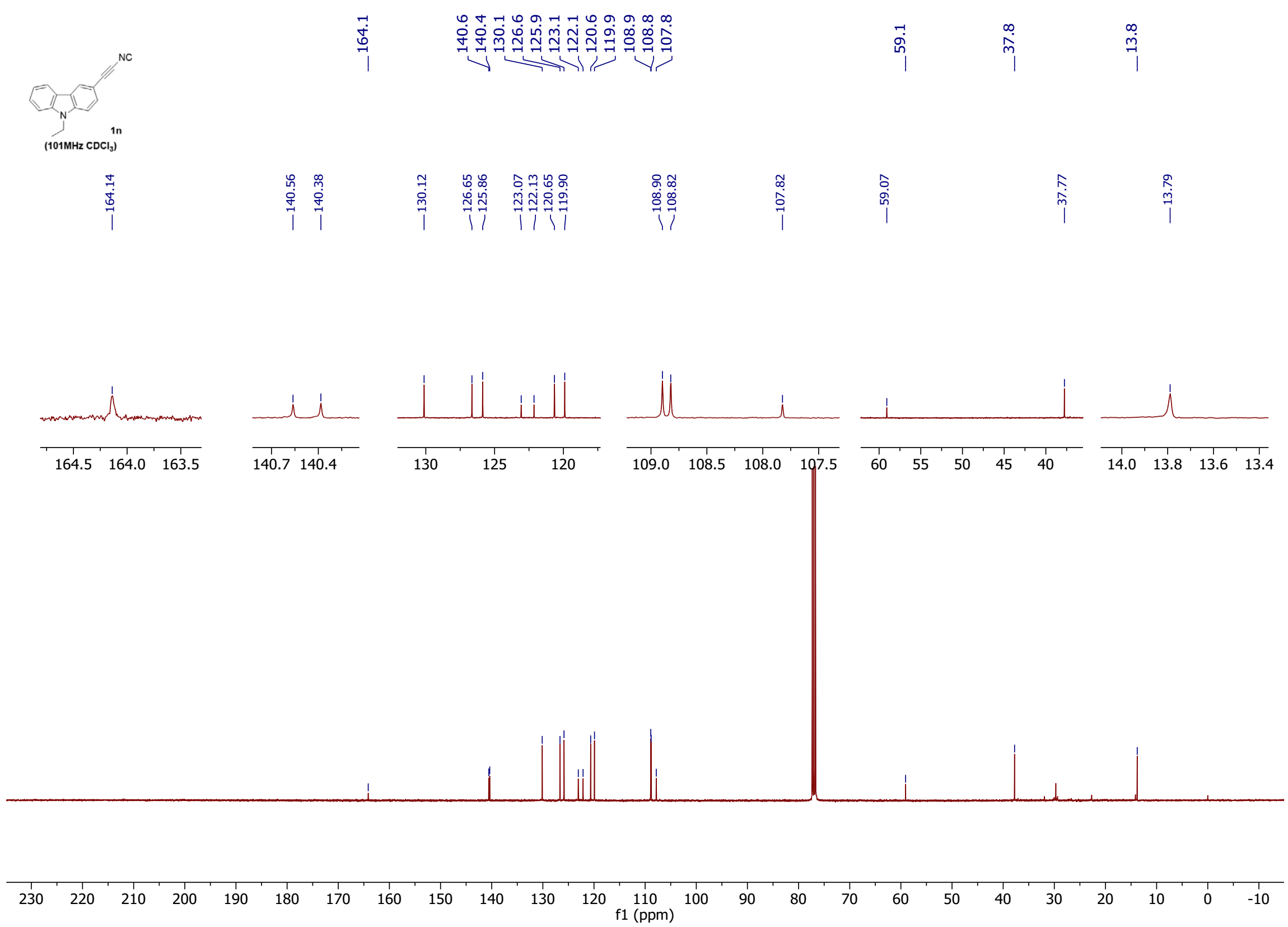
NC
1n
(101MHz CDCl3)
164.1
140.6
140.4
130.1
126.6
125.9
123.1
122.1
120.6
119.9
108.9
108.8
107.8
59.1
37.8
13.8
f1 (ppm)

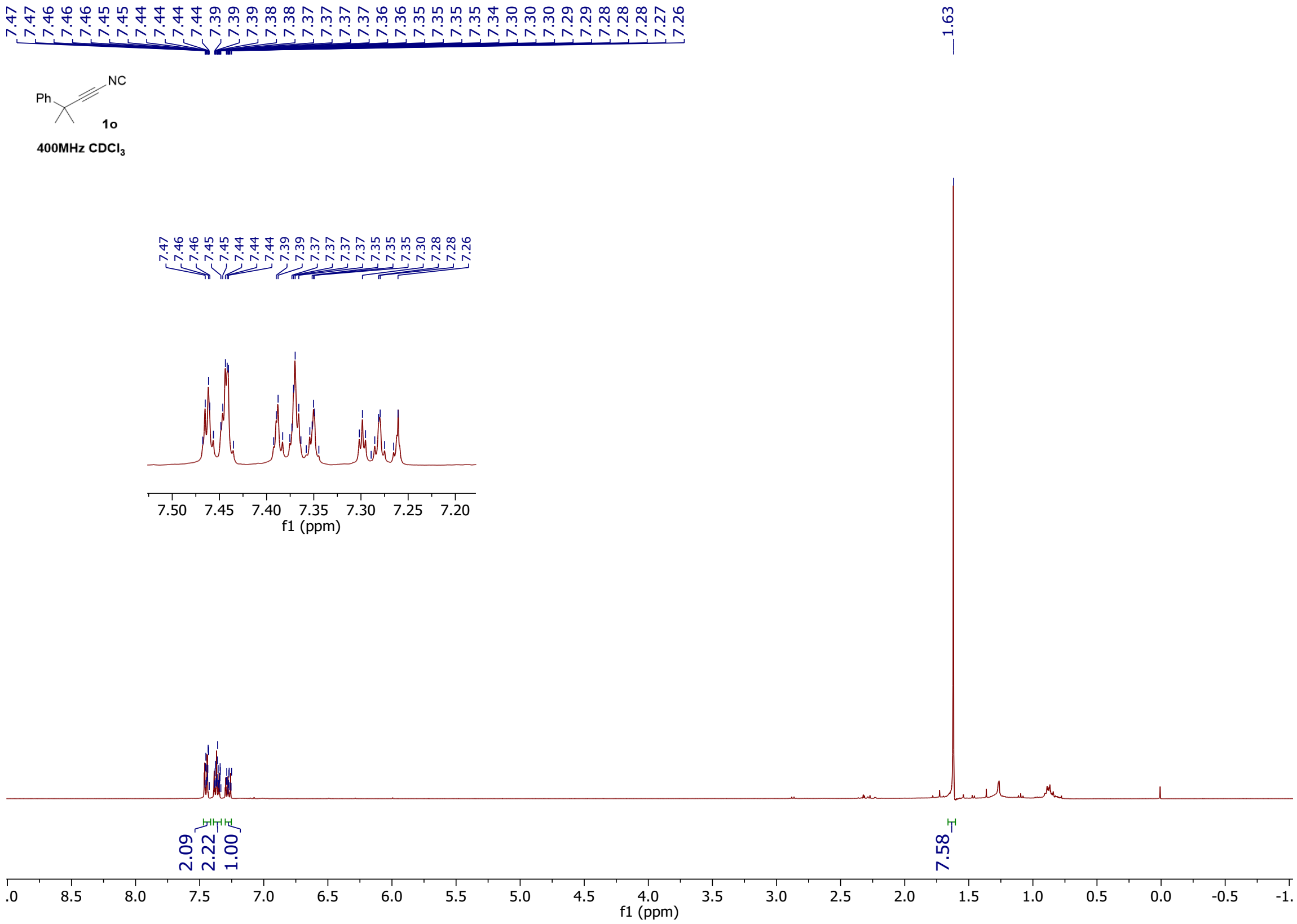

7.47 7.47 7.46 7.46 7.45 7.45 7.44 7.44 7.44 7.44 7.39 7.39 7.39 7.38 7.37 7.37 7.37 7.37 7.36 7.36 7.35 7.35 7.35 7.35 7.34 7.30 7.30 7.29 7.29 7.28 7.28 7.28 7.27 7.26
1.63
Ph
NC
1o
400MHz $CDCl_3$
7.47 7.46 7.46 7.45 7.45 7.44 7.44 7.44 7.39 7.39 7.37 7.37 7.37 7.37 7.35 7.35 7.35 7.30 7.28 7.28 7.26
7.50 7.45 7.40 7.35 7.30 7.25 7.20
f1 (ppm)
2.09
2.22
1.00
7.58
9.0 8.5 8.0 7.5 7.0 6.5 6.0 5.5 5.0 4.5 4.0 3.5 3.0 2.5 2.0 1.5 1.0 0.5 0.0 -0.5 -1.0
f1 (ppm)

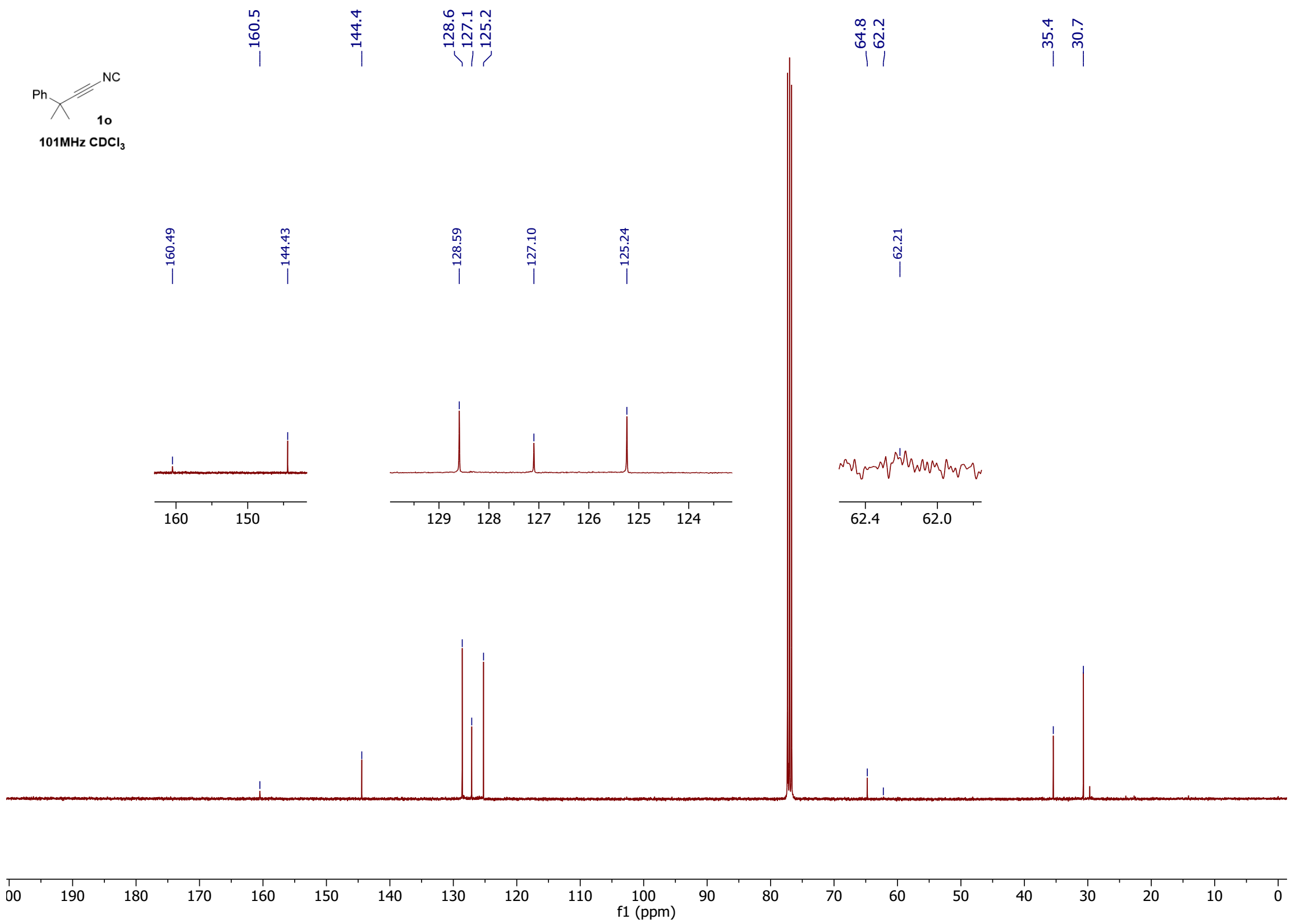

160.5
144.4
128.6
127.1
125.2
64.8
62.2
35.4
30.7
Ph
NC
1o
101MHz $CDCl_3$
160.49
144.43
128.59
127.10
125.24
62.21
160 150
129 128 127 126 125 124
62.4 62.0
200 190 180 170 160 150 140 130 120 110 100 90 80 70 60 50 40 30 20 10 0
f1 (ppm)

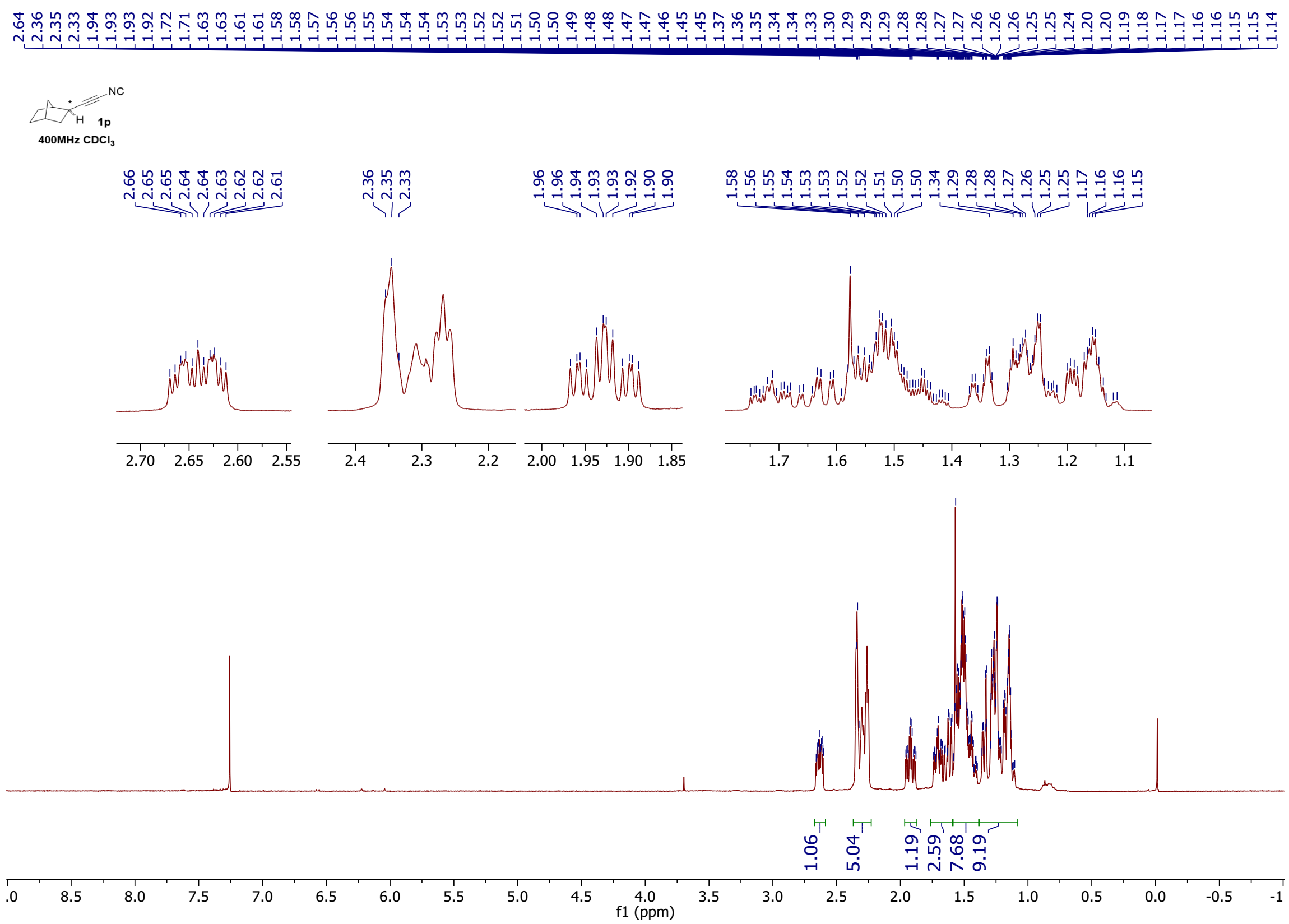
2.64 2.36 2.35 2.33 1.94 1.93 1.93 1.92 1.72 1.71 1.63 1.63 1.61 1.61 1.58 1.58 1.57 1.56 1.56 1.55 1.54 1.54 1.54 1.53 1.53 1.52 1.52 1.51 1.50 1.50 1.49 1.48 1.48 1.47 1.47 1.46 1.45 1.45 1.37 1.36 1.35 1.34 1.34 1.33 1.30 1.29 1.29 1.29 1.28 1.28 1.27 1.27 1.26 1.26 1.26 1.25 1.25 1.24 1.20 1.20 1.19 1.18 1.17 1.17 1.16 1.16 1.15 1.15 1.14
NC
H
1p
400MHz CDCl3
1.06
5.04
1.19
2.59
7.68
9.19
f1 (ppm)

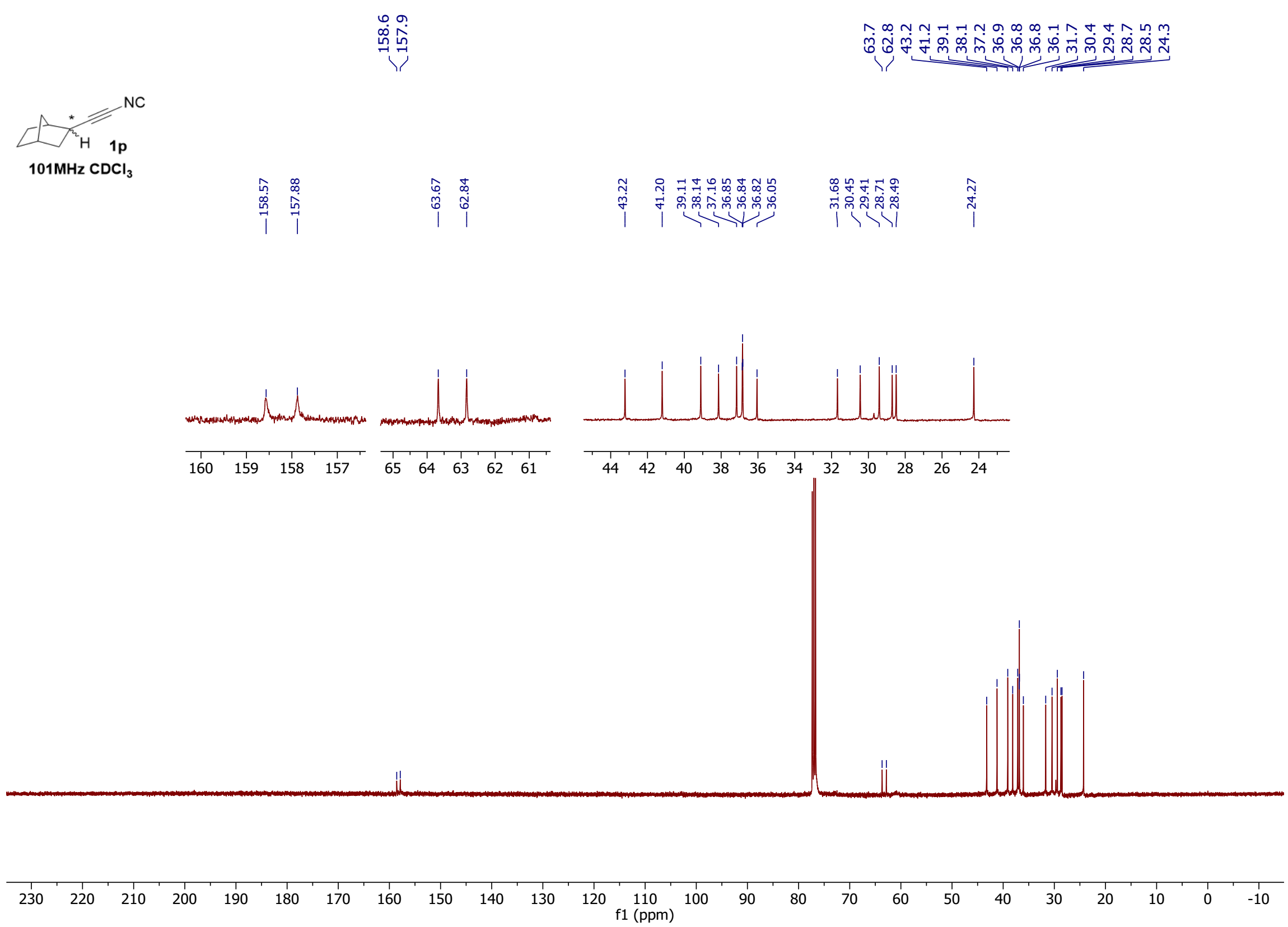
158.6 157.9
63.7 62.8 43.2 41.2 39.1 38.1 37.2 36.9 36.8 36.8 36.1 31.7 30.4 29.4 28.7 28.5 24.3
NC
H
1p
101MHz CDCl3
158.57 157.88
63.67 62.84
43.22 41.20 39.11 38.14 37.16 36.85 36.84 36.82 36.05 31.68 30.45 29.41 28.71 28.49 24.27
f1 (ppm)

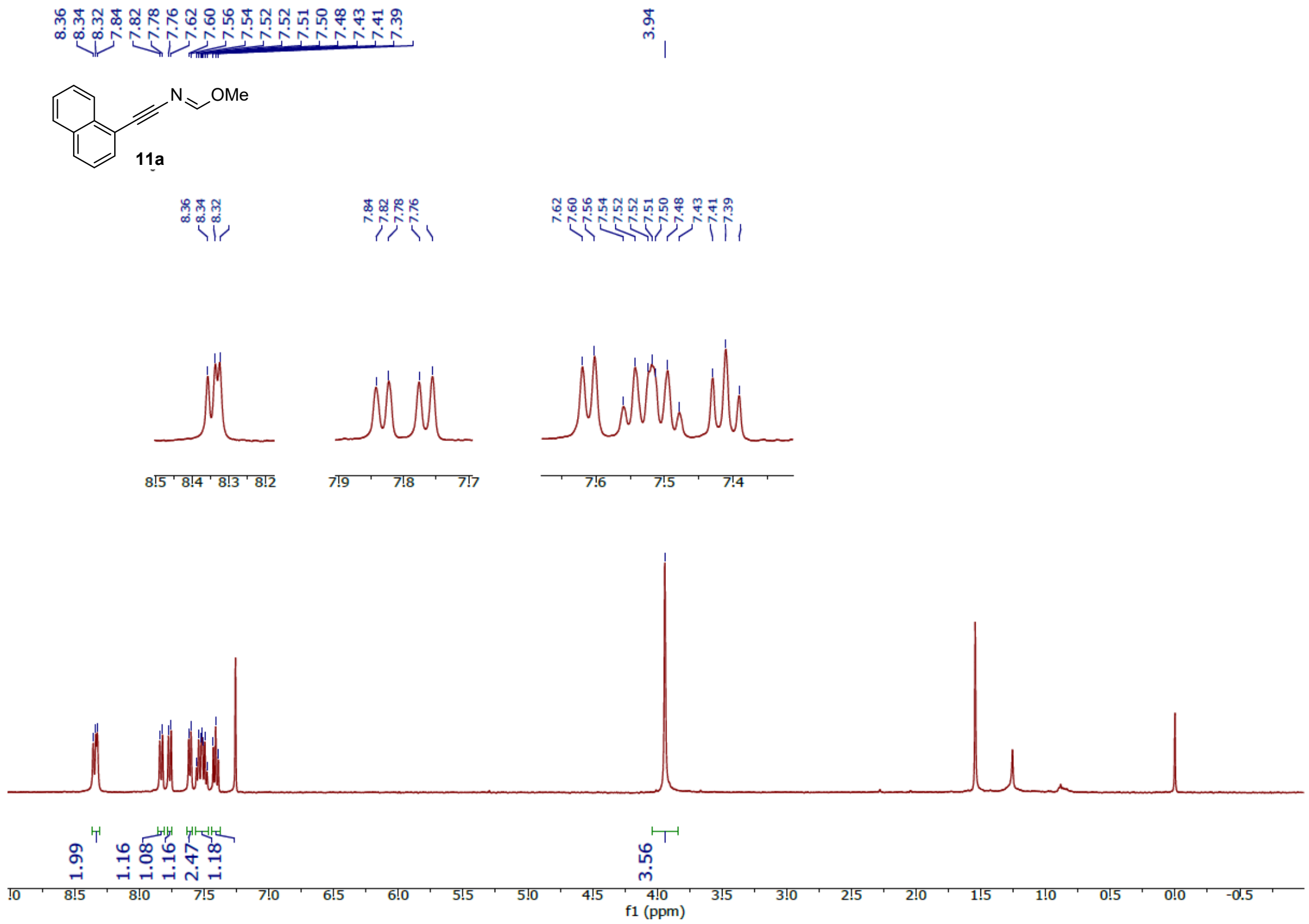
8.36
8.34
8.32
7.84
7.82
7.78
7.76
7.62
7.60
7.56
7.54
7.52
7.52
7.51
7.50
7.48
7.43
7.41
7.39
3.94
OMe
N
11a
1.99
1.16
1.08
1.16
2.47
1.18
3.56
f1 (ppm)

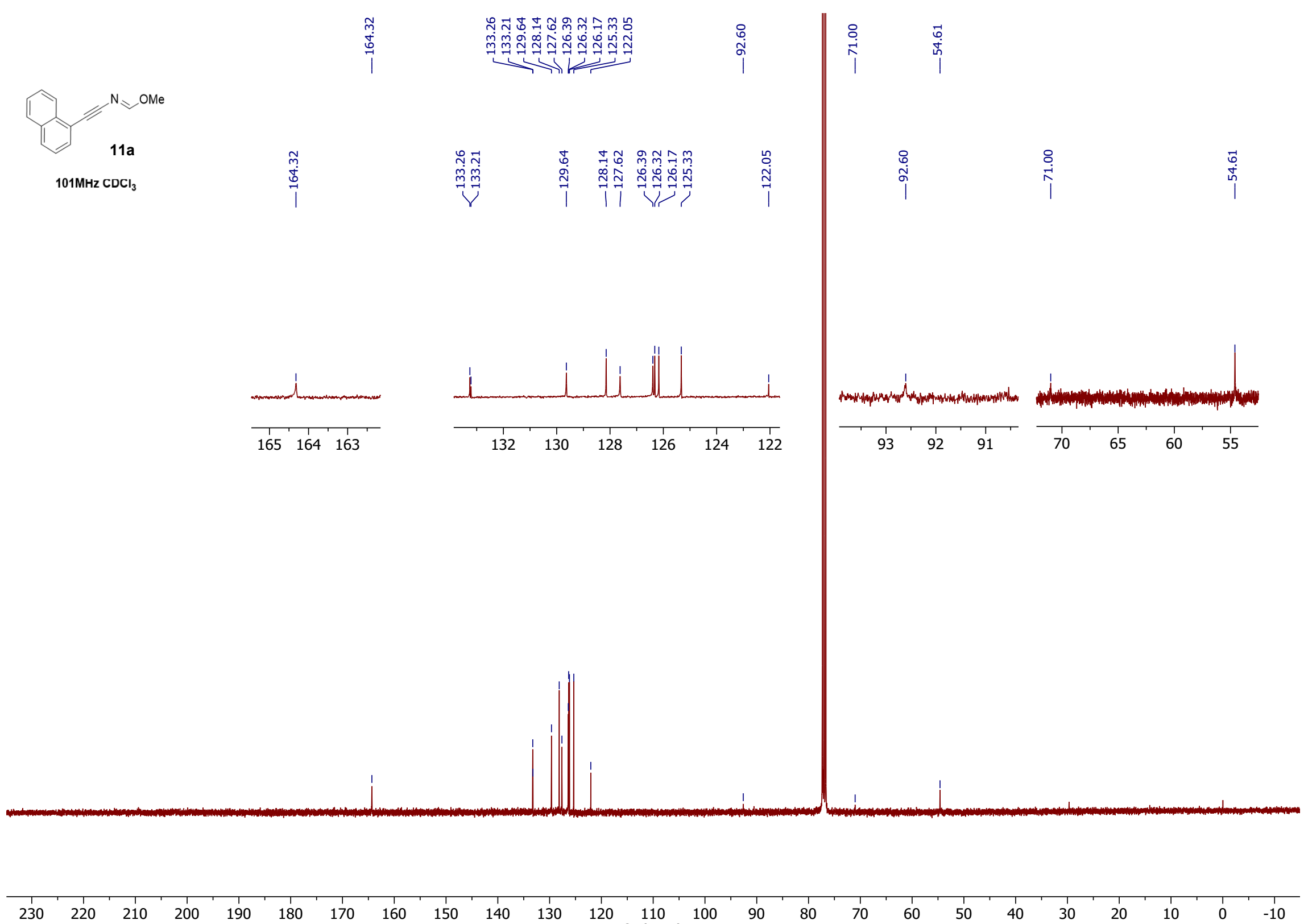
164.32
133.26
133.21
129.64
128.14
127.62
126.39
126.32
126.17
125.33
122.05
92.60
71.00
54.61
OMe
N
11a
101MHz CDCl3
f1 (ppm)

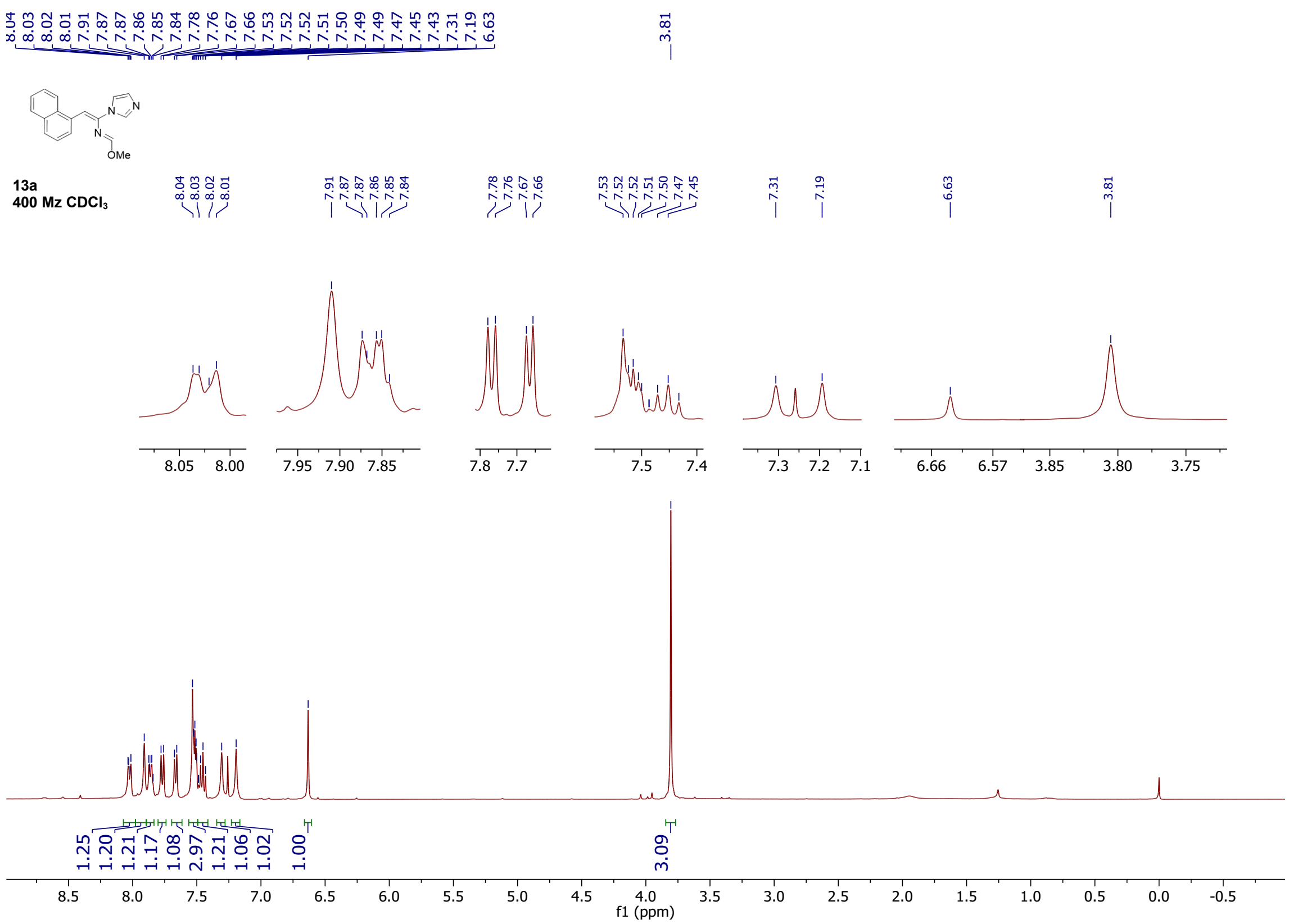
13a
400 Mz CDCl3
f1 (ppm)

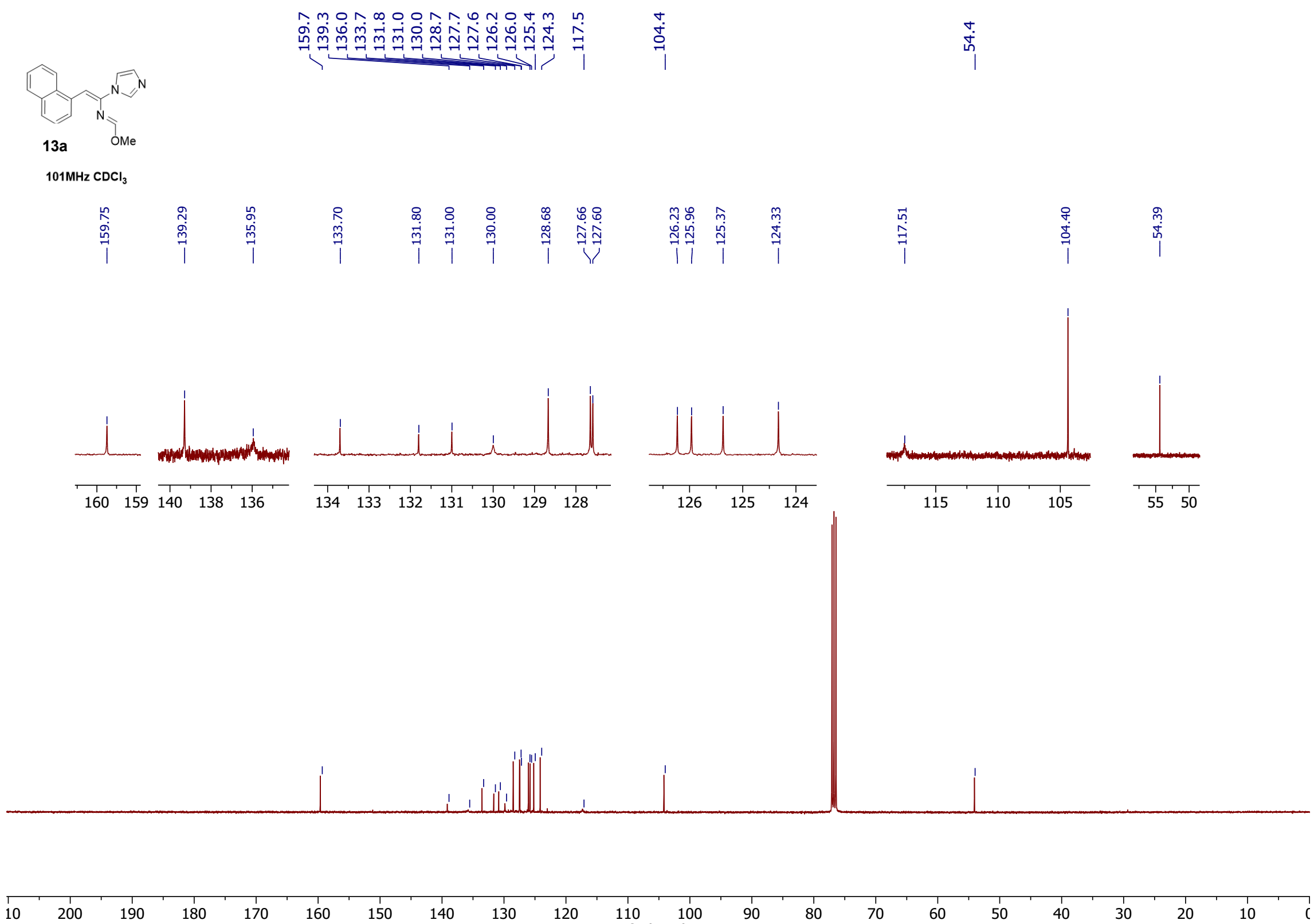
13a
101MHz CDCl3
f1 (ppm)

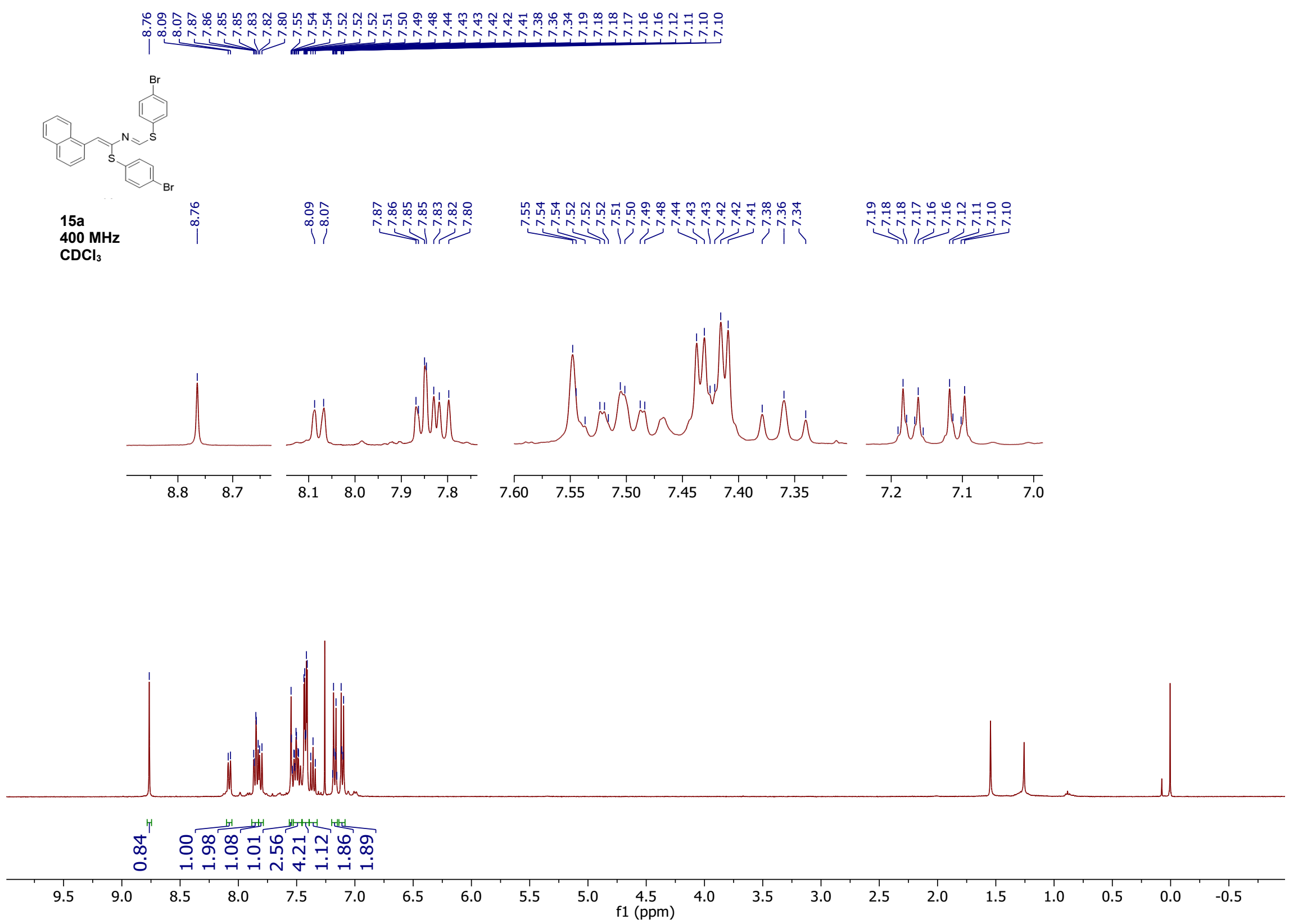

15a
400 MHz
CDCl3
8.76, 8.09, 8.07, 7.87, 7.86, 7.85, 7.85, 7.83, 7.82, 7.80, 7.55, 7.54, 7.54, 7.52, 7.52, 7.52, 7.51, 7.50, 7.49, 7.48, 7.44, 7.43, 7.43, 7.42, 7.42, 7.41, 7.38, 7.36, 7.34, 7.19, 7.18, 7.18, 7.17, 7.16, 7.16, 7.12, 7.11, 7.10, 7.10
0.84
1.00
1.98
1.08
1.01
2.56
4.21
1.12
1.86
1.89
f1 (ppm)

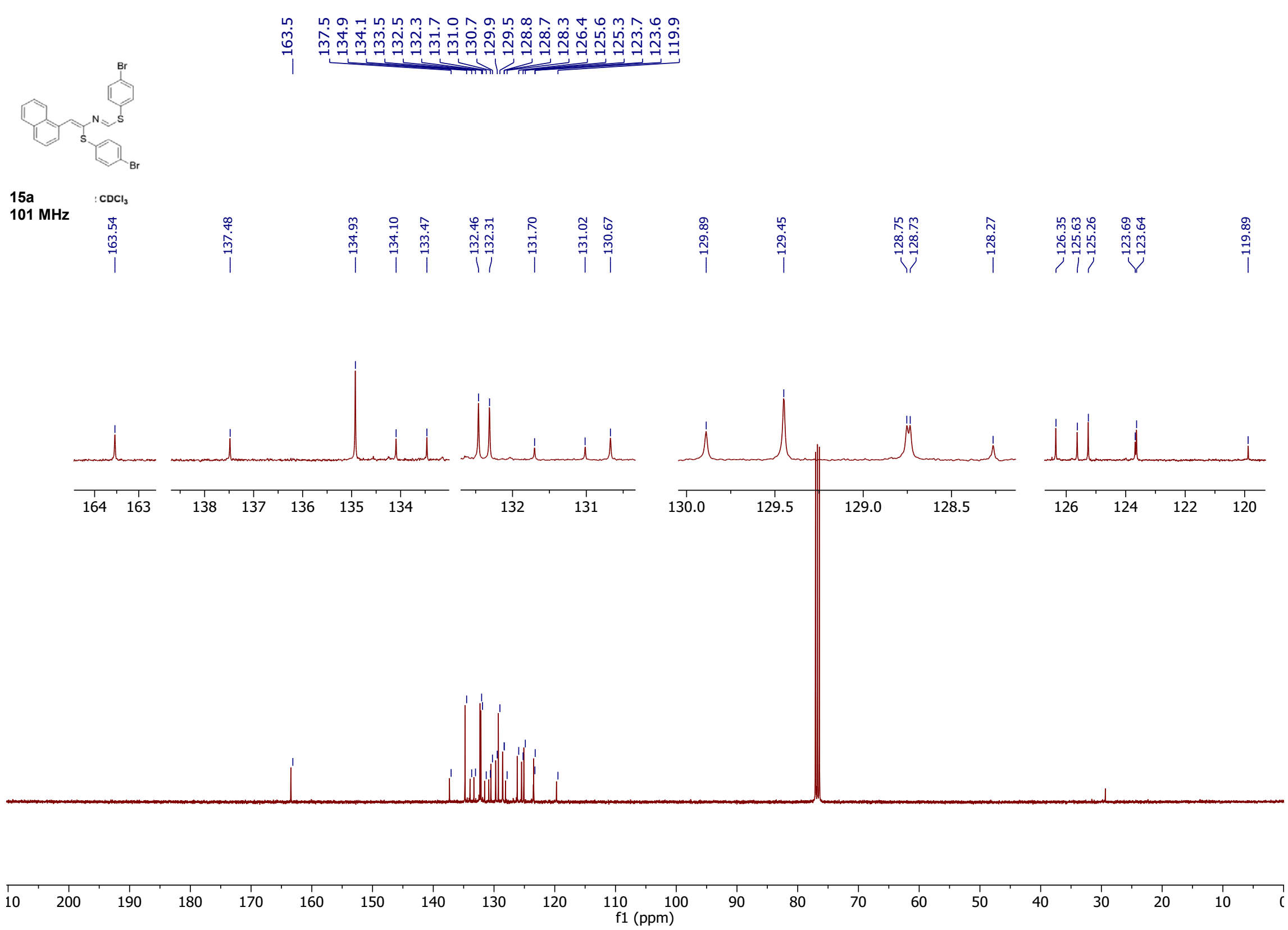

15a
101 MHz
CDCl3
163.5, 137.5, 134.9, 134.1, 133.5, 132.5, 132.3, 131.7, 131.0, 130.7, 129.9, 129.5, 128.8, 128.7, 128.3, 126.4, 125.6, 125.3, 123.7, 123.6, 119.9
163.54, 137.48, 134.93, 134.10, 133.47, 132.46, 132.31, 131.70, 131.02, 130.67, 129.89, 129.45, 128.75, 128.73, 128.27, 126.35, 125.63, 125.26, 123.69, 123.64, 119.89
f1 (ppm)

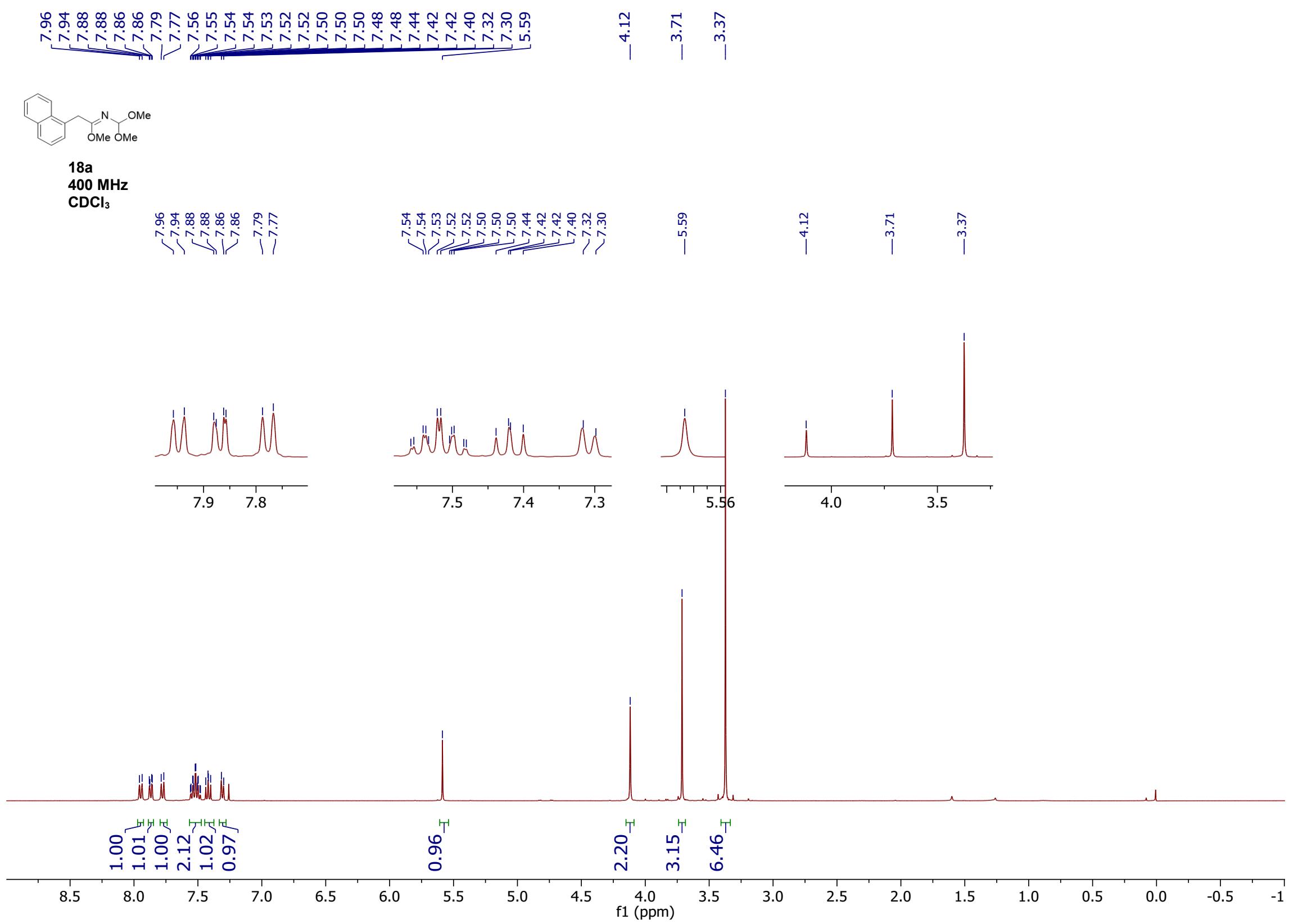
N
OMe
OMe OMe
18a
400 MHz
CDCl3
7.96 7.94 7.88 7.88 7.86 7.86 7.79 7.77 7.56 7.55 7.54 7.54 7.53 7.52 7.52 7.50 7.50 7.50 7.48 7.44 7.42 7.42 7.40 7.32 7.30 5.59
4.12
3.71
3.37
1.00 1.01 1.00 2.12 1.02 0.97
0.96
2.20
3.15
6.46
f1 (ppm)

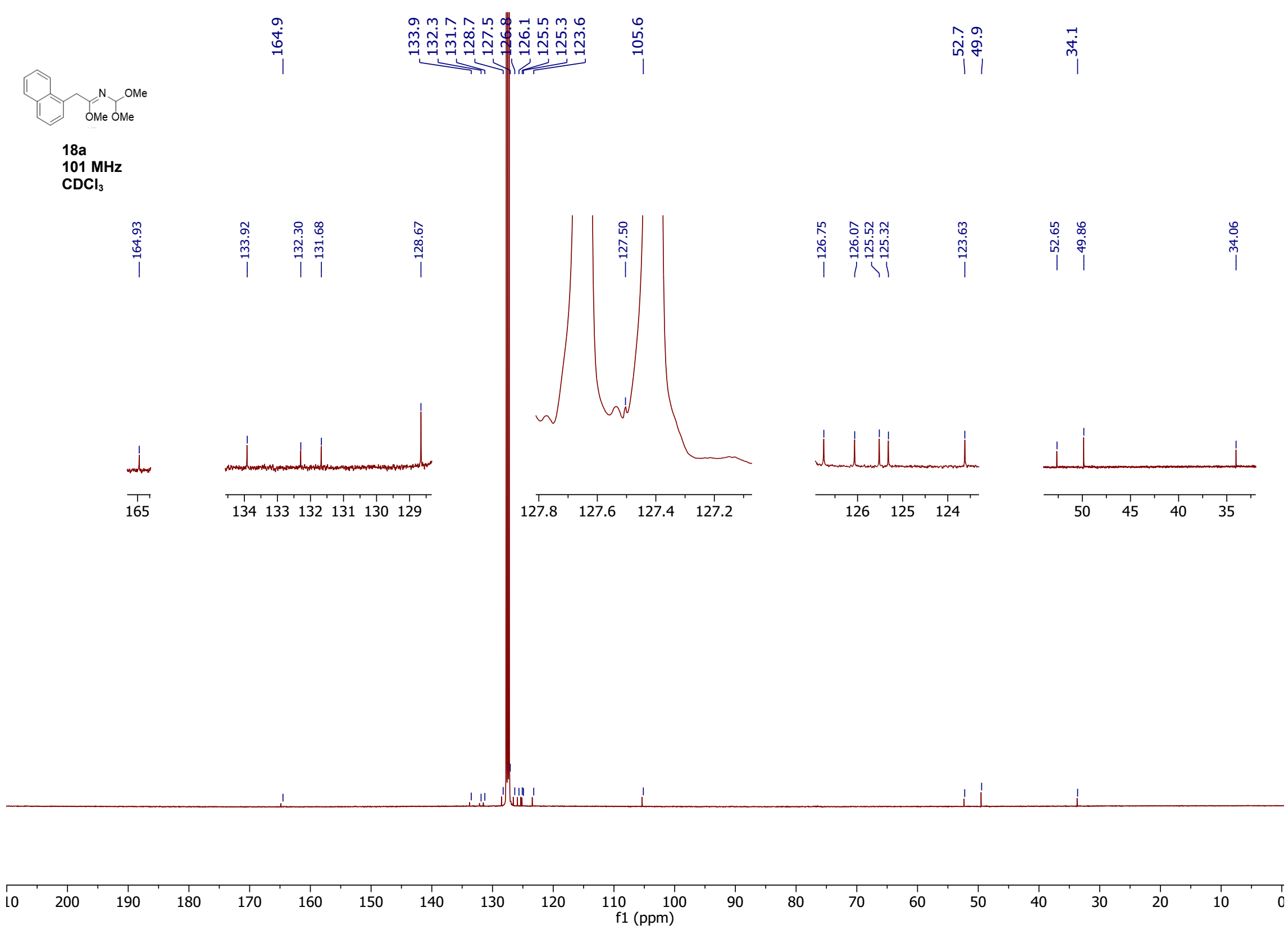
N
OMe
OMe OMe
18a
101 MHz
CDCl3
164.9
133.9 132.3 131.7 128.7 127.5 126.8 126.1 125.5 125.3 123.6
105.6
52.7 49.9
34.1
164.93
133.92
132.30 131.68
128.67
127.50
126.75 126.07 125.52 125.32
123.63
52.65 49.86
34.06
f1 (ppm)

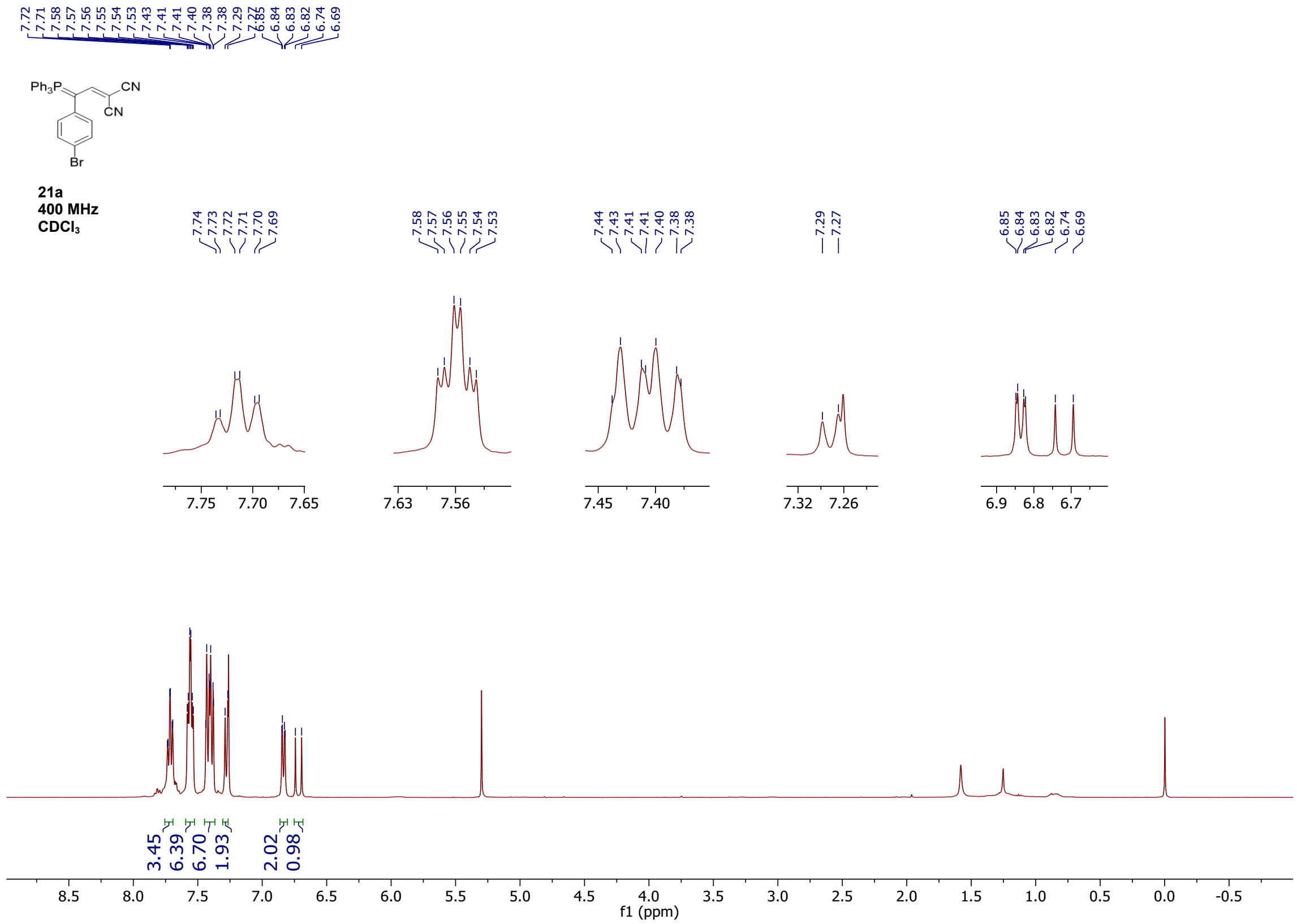
21a
400 MHz
CDCl3
7.72 7.71 7.58 7.57 7.56 7.55 7.54 7.53 7.43 7.41 7.41 7.40 7.38 7.38 7.29 7.27 6.85 6.84 6.83 6.82 6.74 6.69
7.74 7.73 7.72 7.71 7.70 7.69
7.58 7.57 7.56 7.55 7.54 7.53
7.44 7.43 7.41 7.41 7.40 7.38 7.38
7.29 7.27
6.85 6.84 6.83 6.82 6.74 6.69
3.45 6.39 6.70 1.93 2.02 0.98
f1 (ppm)

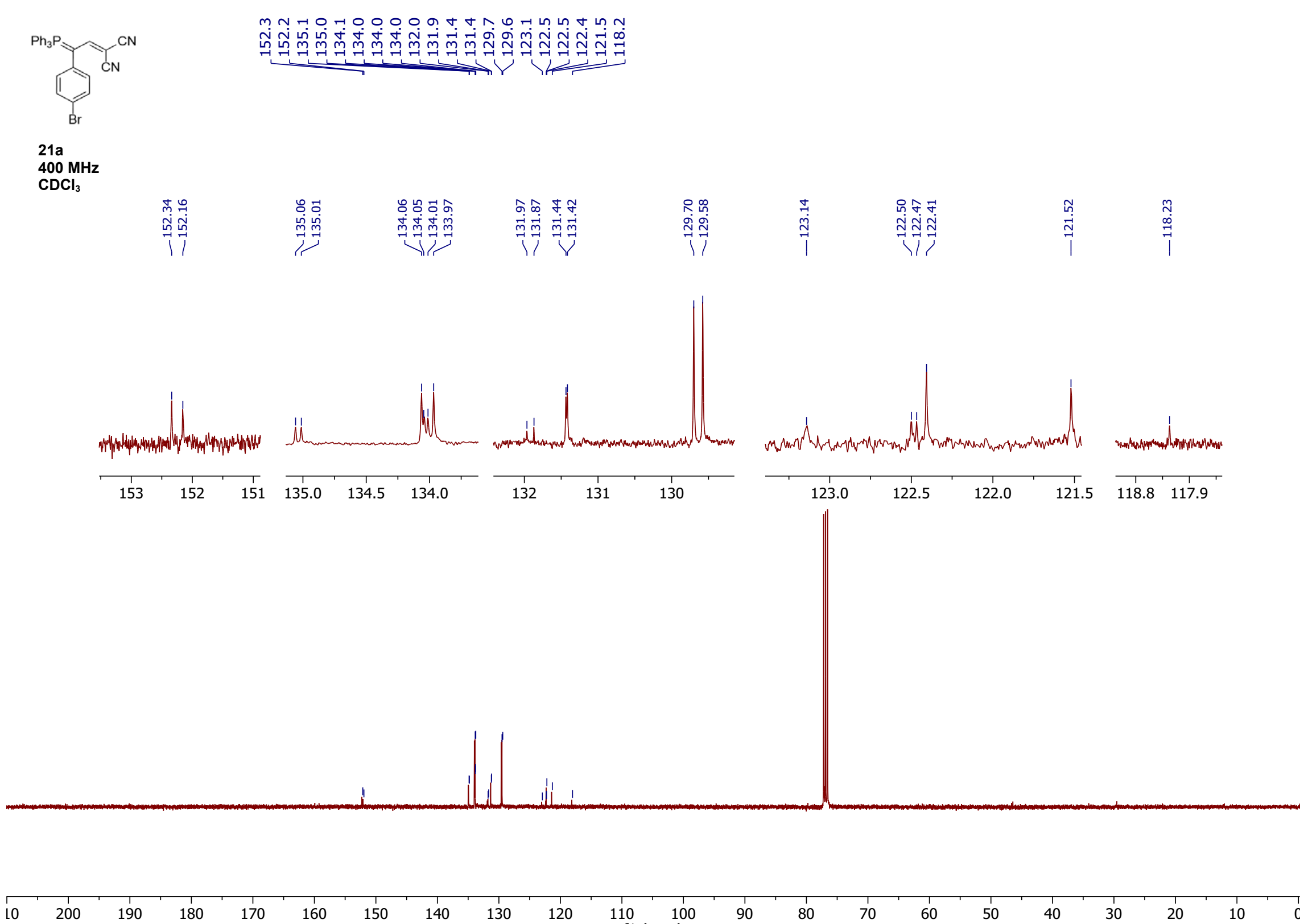
21a
400 MHz
CDCl3
152.3 152.2 135.1 135.0 134.1 134.0 134.0 134.0 132.0 131.9 131.4 131.4 129.7 129.6 123.1 122.5 122.5 122.4 121.5 118.2
152.34 152.16
135.06 135.01
134.06 134.05 134.01 133.97
131.97 131.87 131.44 131.42
129.70 129.58
123.14
122.50 122.47 122.41
121.52
118.23
f1 (ppm)

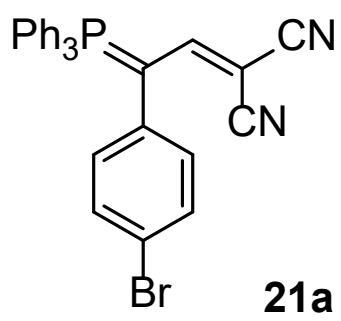

21a

^{31}P NMR
161.95 MHz
$CDCl_3$

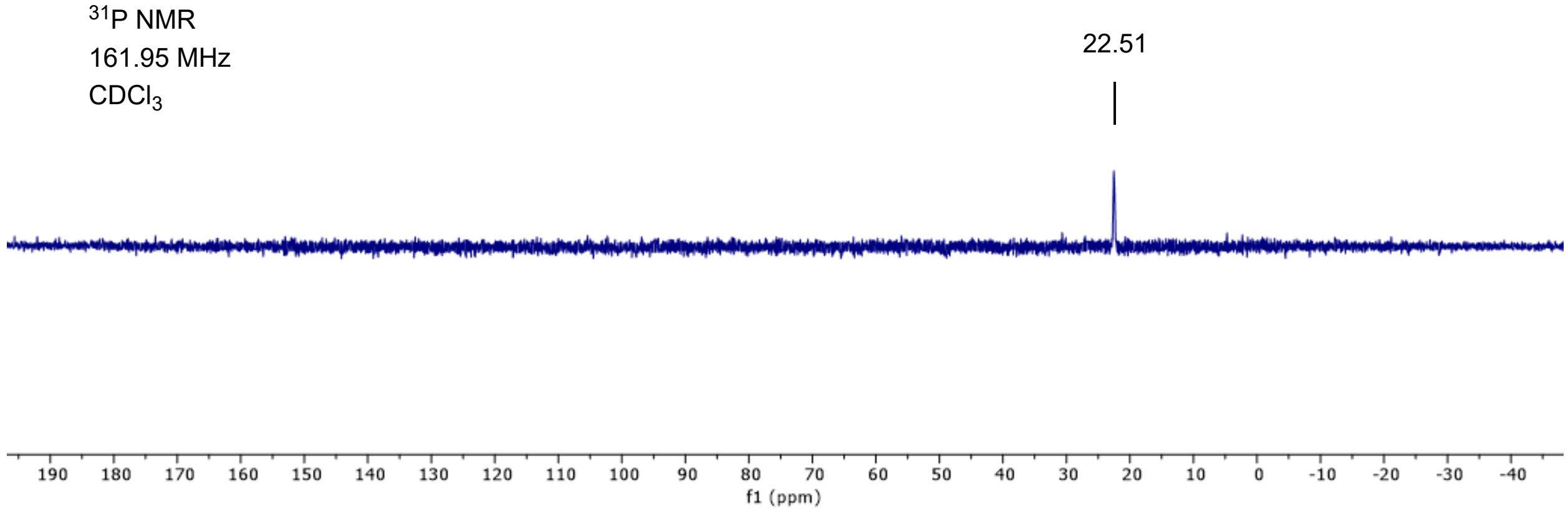

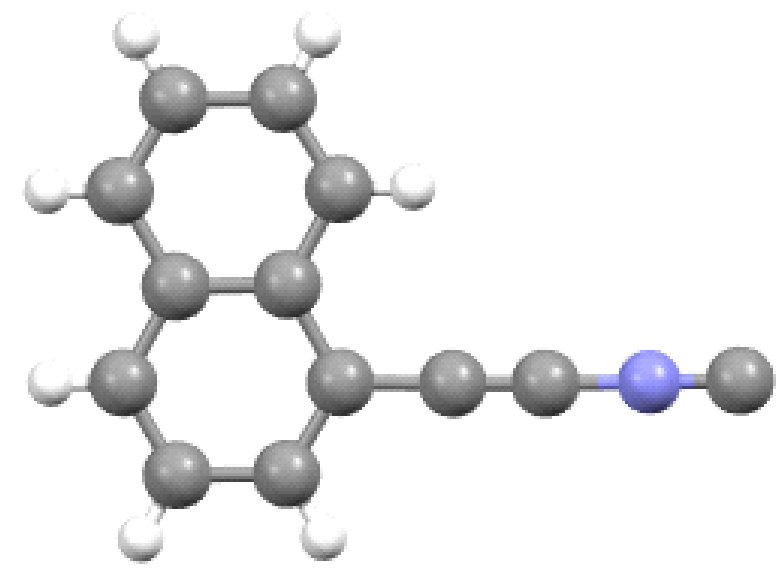

Figure S1: Structure of **1j** (CCDC # 2416456)

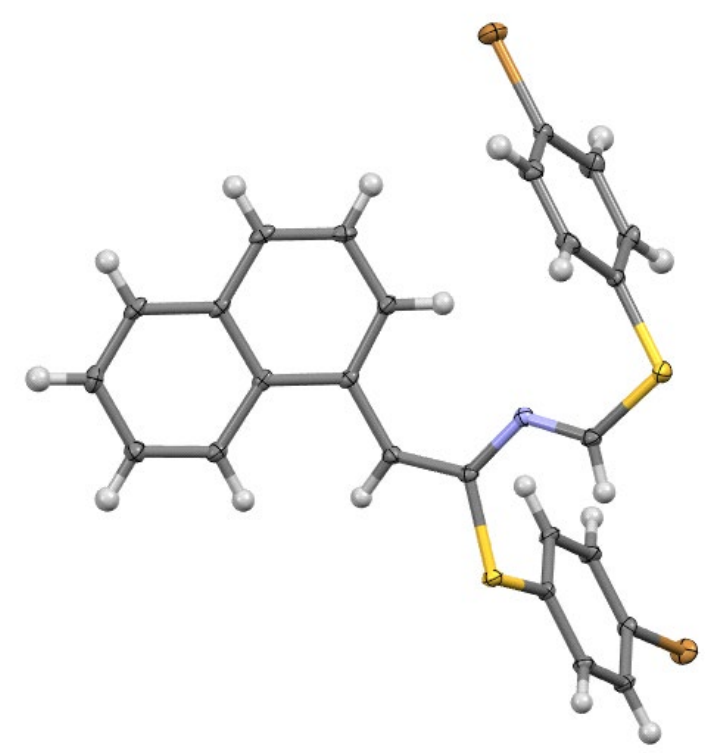

Figure S2: Structure of **15a** (CCDC # 2425384)

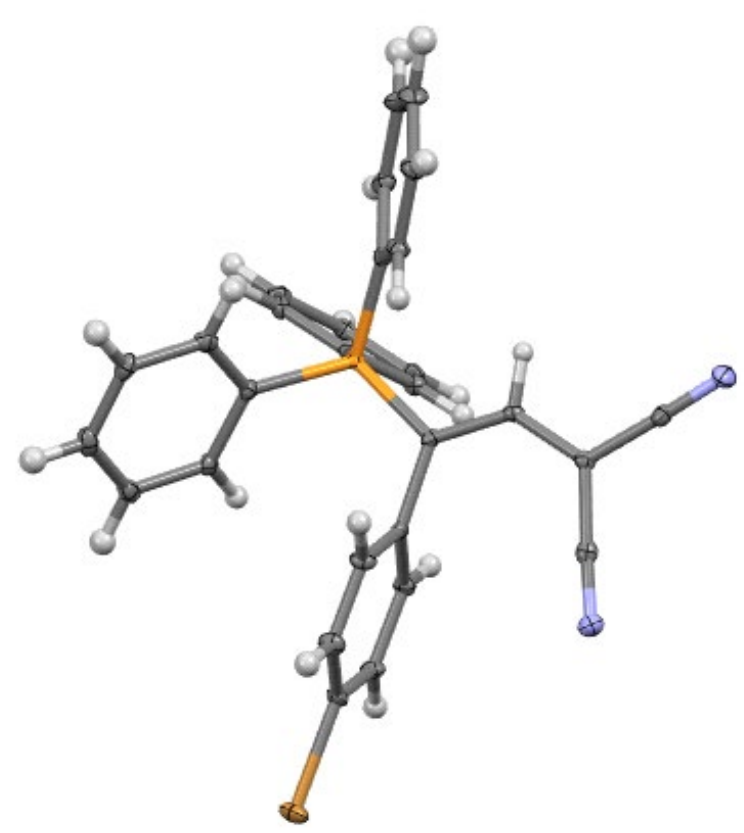

Figure S3: Structure of **21a** (CCDC# 2416617)

Table S2. Citations of the Top 10 Isocyanoalkanes

1

7188-38-7

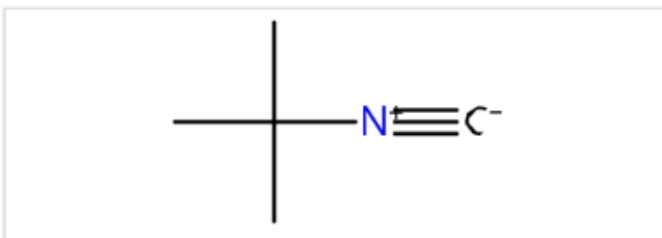

C_5H_9N

tert-Butyl isocyanide

References	Reactions	Suppliers
6,790	36K	57

2

931-53-3

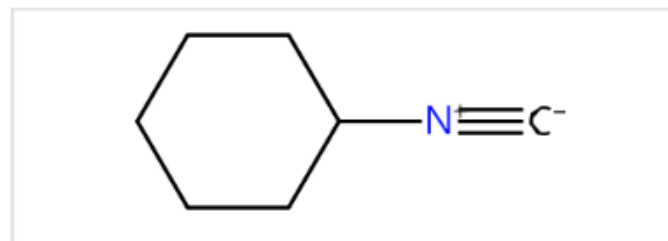

$C_7H_{11}N$

Cyclohexyl isocyanide

References	Reactions	Suppliers
4,333	19K	75

3

2999-46-4

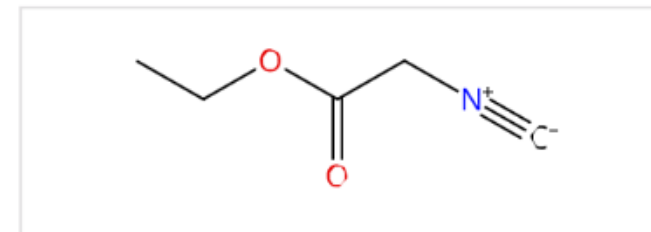

$C_5H_7NO_2$

Ethyl isocyanoacetate

References	Reactions	Suppliers
2,234	8,976	79

4

10340-91-7

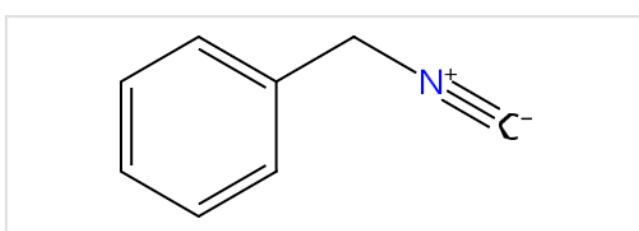

C_8H_7N

Benzyl isocyanide

References	Reactions	Suppliers
2,153	7,671	68

5

593-75-9

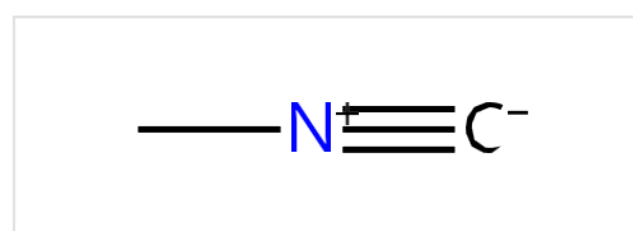

C_2H_3N

Methyl isocyanide

References	Reactions	Suppliers
1,591	924	35

6

39687-95-1

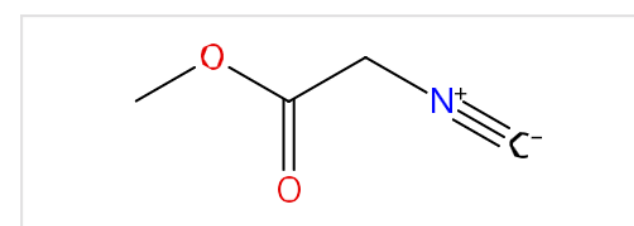

$C_4H_5NO_2$

Methyl isocyanoacetate

References	Reactions	Suppliers
1,240	3,770	77

7

2769-64-4

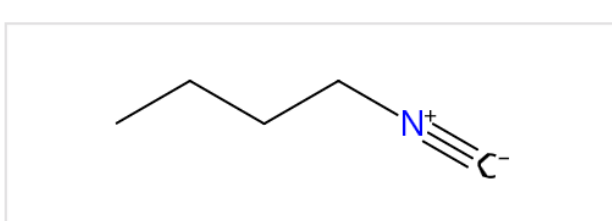

C_5H_9N

Butyl isocyanide

References	Reactions	Suppliers
1,152	2,388	56

8

14542-93-9

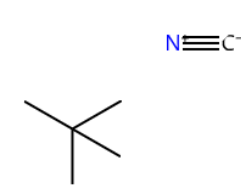

$C_9H_{17}N$

2-Isocyano-2,4,4-trimethylpentane

References	Reactions	Suppliers
1,073	3,398	62

9

598-45-8

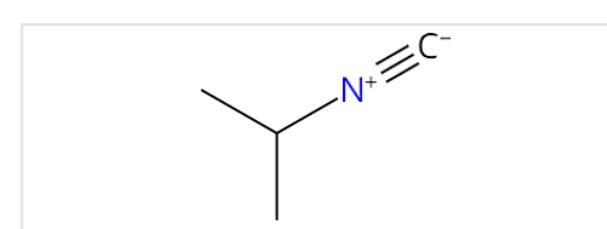

C_4H_7N

Propane, 2-isocyano-

References	Reactions	Suppliers
536	1,232	37

10

22110-53-8

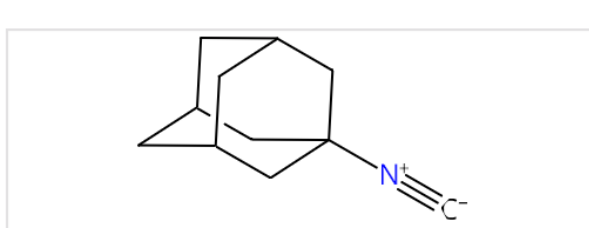

$C_{11}H_{15}N$

1-Adamantyl isocyanide

References	Reactions	Suppliers
492	1,131	70

Figure S4: Citations of the Top 10 Isocyanoalkanes over Time

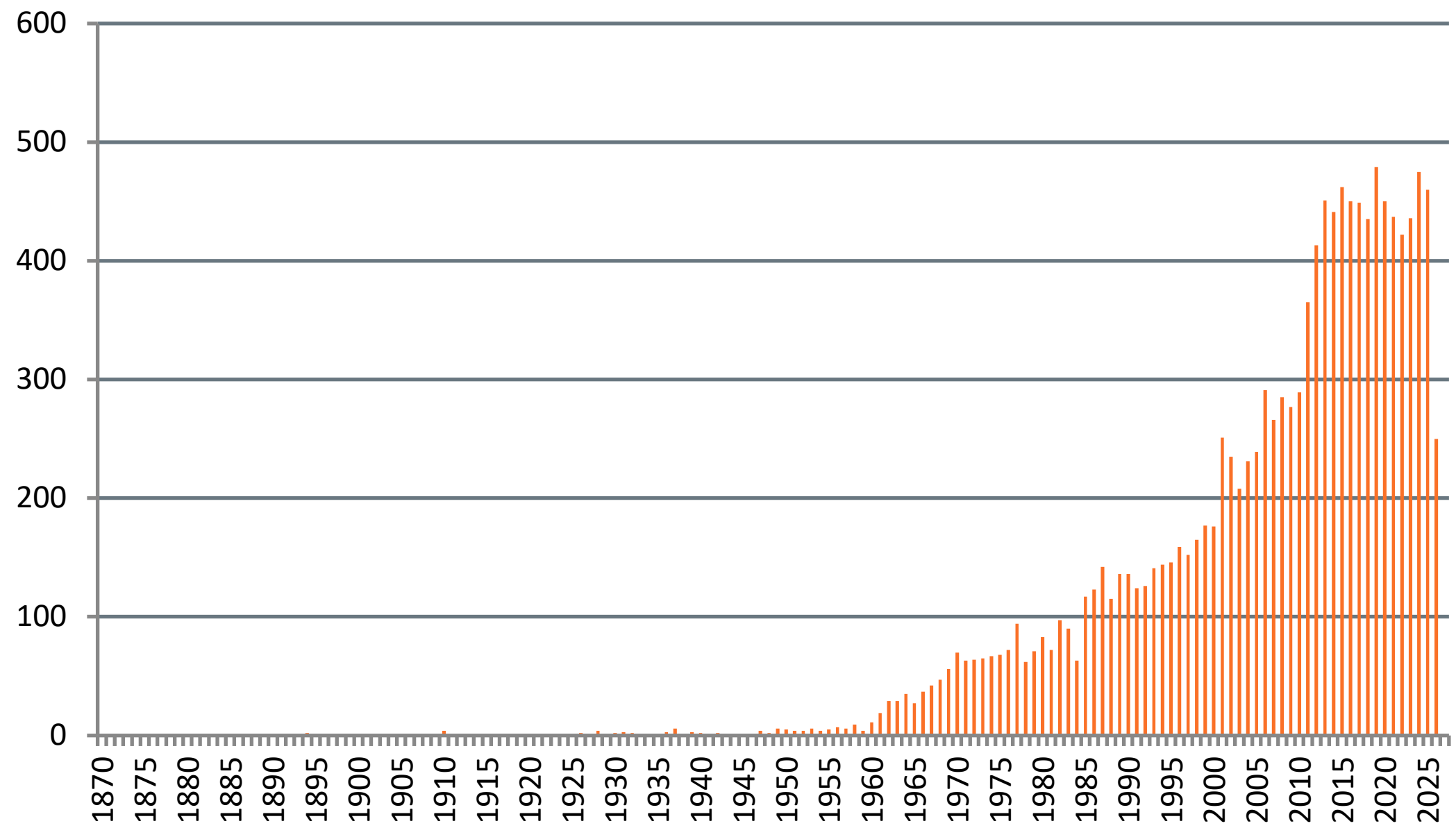

Table S3. Citations of the Top 10 Isocyano*alkenes*

1

580-74-5

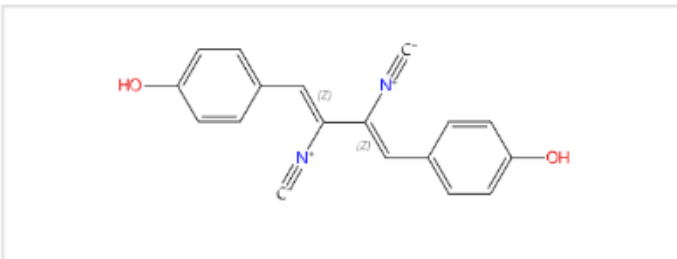
Double bond geometry shown

$C_{18}H_{12}N_2O_2$

Xanthocillin X

75 References | 11 Reactions | 15 Suppliers

2

1121-57-9

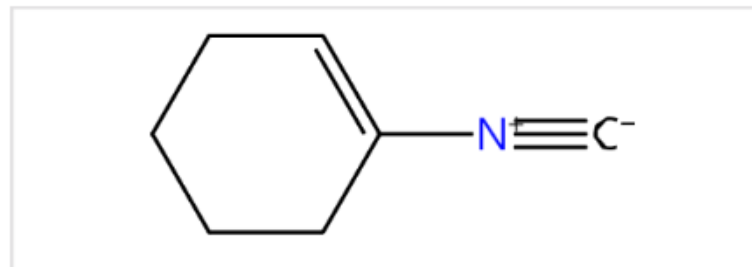

C_7H_9N

1-Isocyanocyclohexene

71 References | 481 Reactions | 27 Suppliers

3

14668-82-7

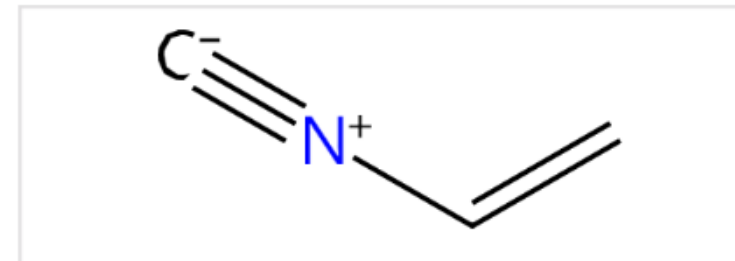

C_3H_3N

Ethene, isocyano-

69 References | 19 Reactions | 1 Supplier

4

42303-26-4

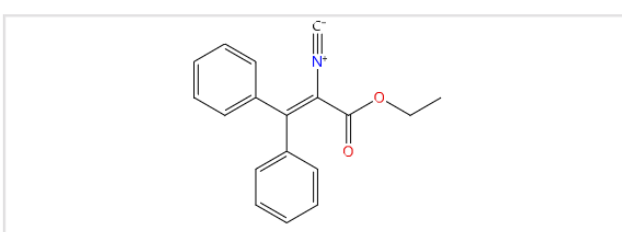

$C_{18}H_{15}NO_2$

Ethyl 2-isocyano-3,3-diphenyl-2-propenoate

44 References | 125 Reactions | 0 Suppliers

5

4464-33-9

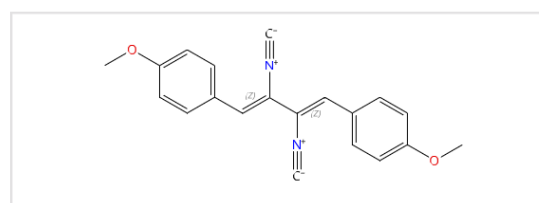
Double bond geometry shown

$C_{20}H_{16}N_2O_2$

1,1′-[(1*Z*,3*Z*)-2,3-Diisocyano-1,3-butadiene-1,4-diyl]bis[4-methoxybenzene]

34 References | 20 Reactions | 33 Suppliers

6

19559-24-1

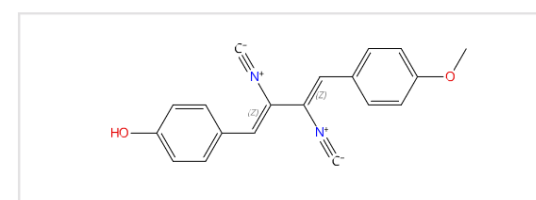
Double bond geometry shown

$C_{19}H_{14}N_2O_2$

4-[(1*Z*,3*Z*)-2,3-Diisocyano-4-(4-methoxyphenyl)-1,3-butadien-1-yl]phenol

23 References | 0 Reactions | 6 Suppliers

7

159934-03-9

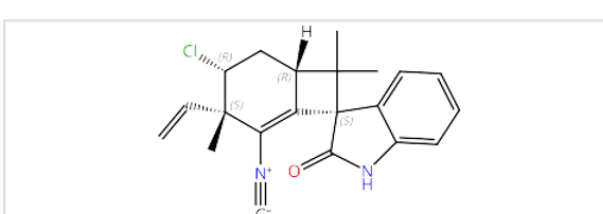
Absolute stereochemistry shown, Rotation (+)

$C_{21}H_{21}ClN_2O$

(1*R*,3*R*,3′*S*,4*S*)-3-Chloro-4-ethenyl-5-isocyano-4,8,8-trimethylspiro[bicyclo[4.2.0]oct-5-ene-7,3′-[3*H*]indol]-2′(1′*H*)-one

22 References | 32 Reactions | 1 Supplier

8

1610564-99-2

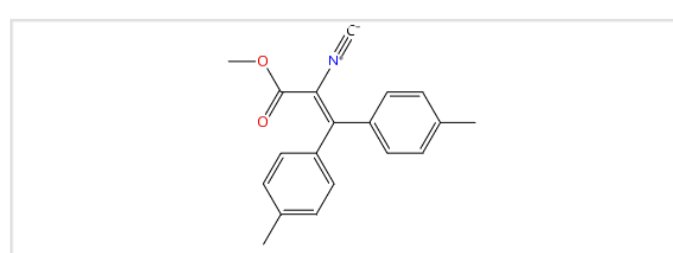

$C_{19}H_{17}NO_2$

Methyl 2-isocyano-3,3-bis(4-methylphenyl)-2-propenoate

20 References | 45 Reactions | 1 Supplier

9

2081899-35-4

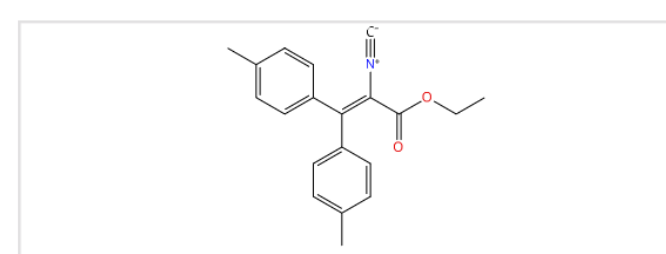

$C_{20}H_{19}NO_2$

2-Propenoic acid, 2-isocyano-3,3-bis(4-methyl phenyl)-, ethyl ester

19 References | 47 Reactions | 0 Suppliers

10

125485-57-6

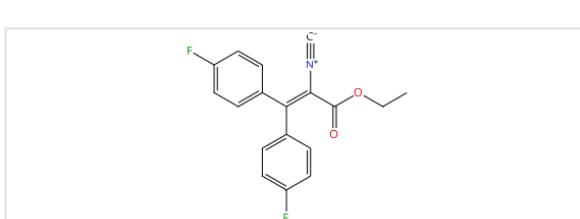

$C_{18}H_{13}F_2NO_2$

Ethyl 3,3-bis(4-fluorophenyl)-2-isocyano-2-propenoate

19 References | 41 Reactions | 1 Supplier

Figure S5: Citations of the Top 10 Isocyano*alkenes* over Time

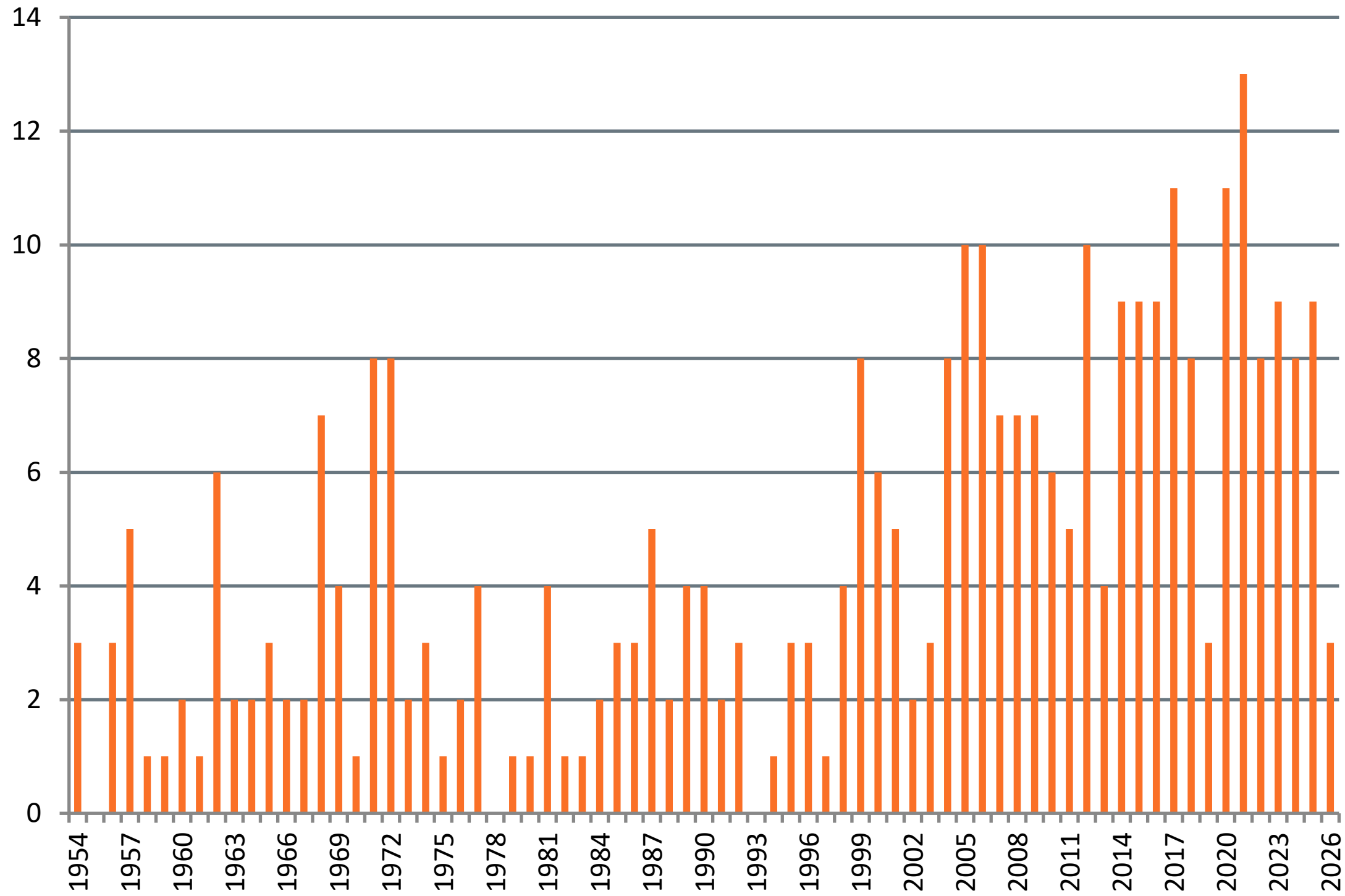

Table S4. Citations of the Top 10 Isocyano*arenes*

1

2769-71-3

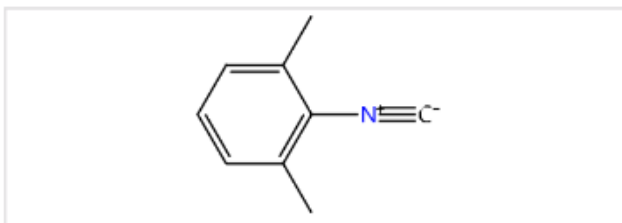

C_9H_9N

2,6-Dimethylphenyl isocyanide

2,381 References	5,780 Reactions	55 Suppliers

2

931-54-4

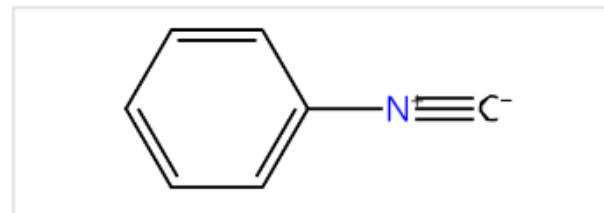

C_7H_5N

Isocyanobenzene

1,272 References	1,557 Reactions	54 Suppliers

3

10349-38-9

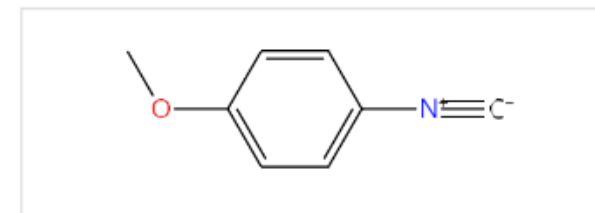

C_8H_7NO

1-Isocyano-4-methoxybenzene

874 References	1,747 Reactions	48 Suppliers

4

7175-47-5

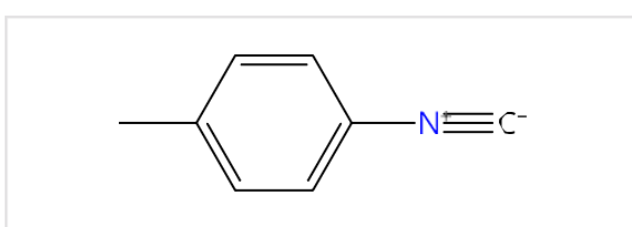

C_8H_7N

p-Tolyl isocyanide

554 References	810 Reactions	28 Suppliers

5

1885-81-0

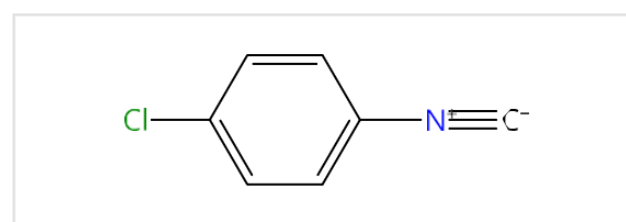

C_7H_4ClN

1-Chloro-4-isocyanobenzene

382 References	868 Reactions	45 Suppliers

6

3128-77-6

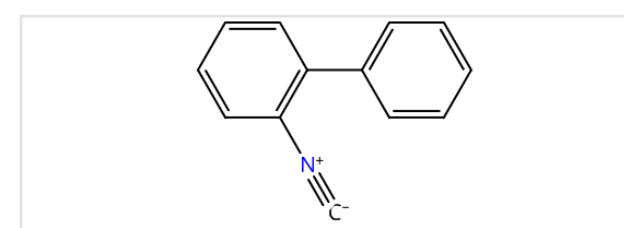

$C_{13}H_9N$

2-Isocyano-1,1′-biphenyl

295 References	1,750 Reactions	34 Suppliers

7

10124-78-4

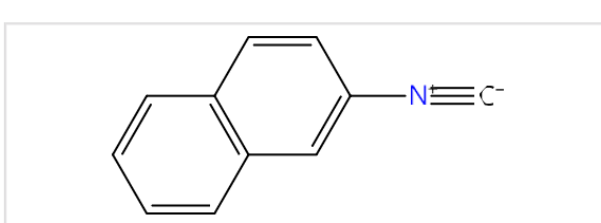

$C_{11}H_7N$

2-Isocyanonaphthalene

282 References	698 Reactions	54 Suppliers

8

935-16-0

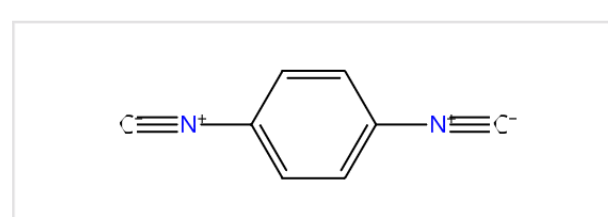

$C_8H_4N_2$

1,4-Diisocyanobenzene

265 References	159 Reactions	41 Suppliers

9

10468-64-1

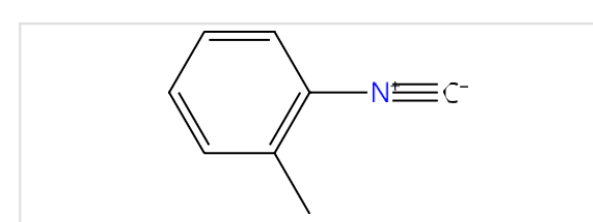

C_8H_7N

1-Isocyano-2-methylbenzene

233 References	343 Reactions	27 Suppliers

10

57116-96-8

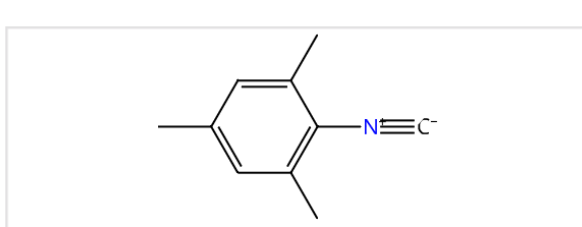

$C_{10}H_{11}N$

2-Isocyano-1,3,5-trimethylbenzene

233 References	387 Reactions	36 Suppliers

Figure S6: Citations of the Top 10 Isocyano*arenes* over Time

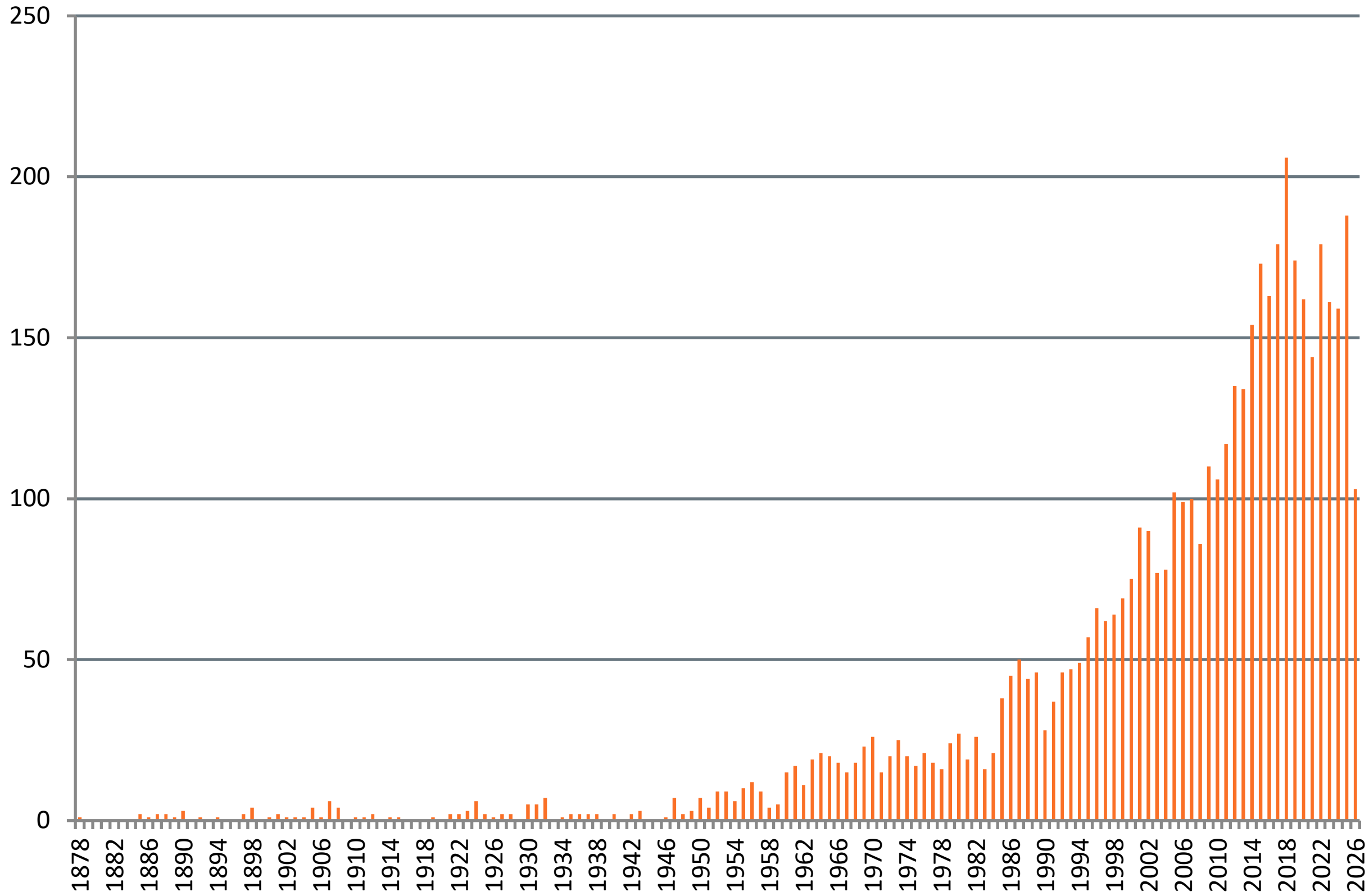

Theoretical Background

The molecular electrostatic potential $V(\mathbf{r})$ is a physical observable and well-established tool for analyzing chemical bonding, molecular charge distributions, and intermolecular interactions, and it is rigorously defined by:[9, 10]

$$V(\mathbf{r}) = \sum_A \frac{Z_A}{|\mathbf{R}_A - \mathbf{r}|} - \int \frac{\rho(\mathbf{r}')d\mathbf{r}}{|\mathbf{r}' - \mathbf{r}|}$$

where Z_A is the charge on nucleus A, located at $\mathbf{R}_A$, and $\rho(\mathbf{r})$ is the electron density. $qV(\mathbf{r})$ gives the interaction energy between a point charge q and the static charge distribution of the molecule. In contrast to e.g. partial charges, $V(\mathbf{r})$ is a physical observable that can be determined experimentally or computationally. $V(\mathbf{r})$ is commonly evaluated on an electron isodensity surface, and maxima ($V_{S,max}$) in the surface $V(\mathbf{r})$ ($V_S(\mathbf{r})$ are found at electron deficient regions, such as the σ-holes of hydrogens and halogens, and reflect sites that interact with nucleophiles. Surface minima ($V_{S,min}$) are typically found at electron rich regions, such as π-bonds or lone pairs, and reflect reactivity towards electrophiles. However, $V_S(\mathbf{r})$ only reflects the tendency for electrostatic interactions and is less useful for characterizing interactions that involve charge transfer and lead to the formation of covalent bonds. It should be noted that $V(\mathbf{r})$ is a one-electron property and as such less sensitive to the computational method, i.e. DFT-functional and basis set, than e.g. relative energies.

The average local ionization energy $I(\mathbf{r})$ is well suited to analyze the propensity for charge transfer interactions with electrophiles that lead to the formation of covalent bonds.[9, 11] It is rigorously defined within Hartree-Fock theory and Kohn-Sham density functional theory by.

$$I(\boldsymbol{r}) = \frac{-1}{\rho(\boldsymbol{r})} \sum_i^{occ} \varepsilon_i \rho_i(\boldsymbol{r})$$

where ε_i is the energy of orbital i, $\rho_i(\mathbf{r})$ is the orbital density, and $\rho(\mathbf{r})$ is the total density. $I(\mathbf{r})$ corresponds to the average energy needed to ionize an electron from the position **r.**
where ε_i is the energy of orbital i, $\rho_i(\mathbf{r})$ is the orbital density, and $\rho(\mathbf{r})$ is the total density. $I(\mathbf{r})$ corresponds to the average energy needed to remove an electron from the position **r.** $I(\mathbf{r})$ like $V(\mathbf{r})$ is generally evaluated on an isodensity surface, and surface minima in $I(\mathbf{r})$ are indicative of sites that interact with electrophiles in chemical reactions.

The local electron attachment energy is a similar property to $I(\mathbf{r})$ but reflect the interaction tendencies towards nucleophiles in charge transfer interactions that lead to the formation of covalent bonds.[9, 12] $E(\mathbf{r})$ is rigorously defined within generalized Kohn-Sham density functional theory by

$$E(\boldsymbol{r}) = \frac{1}{\rho(\boldsymbol{r})} \sum_{i=LUMO}^{\varepsilon_i<0} \varepsilon_i \rho_i(\boldsymbol{r})$$

where ε_i is the energy of orbital *i*, $\rho_i(\mathbf{r})$ is the orbital density, and $\rho(\mathbf{r})$ is the total density. $E(\mathbf{r})$ only has contributions from the virtual (unoccupied) orbitals below the free electron limit. $E(\mathbf{r})$ is justified based on Janak´s theorem and the piece-wise linear energy dependence of the number of electrons. Minima in the surface $E(\mathbf{r})$ reflect sites that are prone to interact with nucleophiles, and the value of $E_{S,min}$ the relative strength of the interaction. $E_S(\mathbf{r})$ have been shown to be particularly useful for characterizing sites for nucleophilic aromatic substititution and conjugate nuchleophilic addition to π-bonds. $E(\mathbf{r})$ is more sensitive to the choice of DFT functional and basis than $V(\mathbf{r})$ and $I(\mathbf{r})$. For the prediction of regioselectivity and relative reactivity of attack by nucleophiles in organic system, standard hybrid functionals, like B3LYP and PBE0, that includes 15-25 % Hartree-Fock exchange have been found to perform well.[9,12,13,14] On the other hand, long-range corrected GGA or hybrid-functionals, such a LC-BLYP, CAM-B3LYP or ωB97X-D are less suitable for predicting regioselectivity.[9,12] When it comes to basis set, it is important to use a polarized basis set augmented by diffuse functions, as the latter are needed for reproducing the sizes and energies of the virtual/unoccupied orbitals.[9,12] The pople-type 6-31+G(d,p) basis has been shown to perform well in several studies.[9,12,13]

Computational procedures

Molecular structures and vibrational frequencies have been computed at the B3LYP-6-31+G(d,p) level using the Gaussian 16 suite of programs.[15] The frequencies have been scaled by 0.9642 before comparing them to experiment.[16] The surface property calculations were based on structures and wavefunctions corrected for solvation effects by invoking the polarizable continuum model and solvent parameters for methanol in the Gaussian 16 computations. Solvation effects were included as the experimental characterization of chemical reactivity was done in organic solvents. It should be noted that changing the solvent has only marginal effects on the results of the surface propery calculations, and methanol was chosen as a representative solvent. All surface property computations were performed at the 0.004 isodensity surface using the hs95ver25 software of Brinck.[9] The 0.004 au isodensity surface has been show to be particularly useful when characterizing sites that reacts with nucleophiles using $E_S(\mathbf{r})$. $V_{S,min}$ obtained at the 0.004 au surface are also closer to the spatial minima in $V_S(\mathbf{r})$ than $V_{S,min}$ obtained at the more commonly used 0.001 au surface.[10] $E_S(\mathbf{r})$ and $V_S(\mathbf{r})$ have been visualized using the UCSF Chimera program.[17]

To estimate relative enthalpies of isocyanides and the isomeric nitriles computations have been performed using (gas phase) the CBS-QB3 method.[18] It is a composite quantum chemical model based on structure optimization and frequency calculation at the B3LYP/6-311G(2d,d,p) level and single point energy at the CCSD(T)/6-311G(d†) level with basis set extrapolation to infinite basis using MP2 and MP4. The CBS-QB3 method is highly accurate with a mean absolute error in energies of 0.87 kcal/mol for the G2 test set consisting of 125 experimental energies (dissociation energies, ionization potentials, electron affinities, and proton affinities).[18]

Geometries, Energies, and (unscaled) Frequencies of optimized structures

1d (C9H5N1) B3LYP/6-31+G(d,p)

Frequencies --	75.1520	80.7975	205.6322
Frequencies --	226.7936	369.5637	373.9047
Frequencies --	409.5337	469.9742	508.9022
Frequencies --	572.1704	623.3439	634.6533
Frequencies --	699.9344	706.4783	774.1751
Frequencies --	854.9049	936.3459	985.4725
Frequencies --	1004.2996	1007.2724	1014.5053
Frequencies --	1053.8894	1104.3007	1187.0020
Frequencies --	1204.2437	1321.4102	1324.3680
Frequencies --	1359.1253	1476.2729	1528.1709
Frequencies --	1615.2876	1645.4481	**2141.2359***
Frequencies --	2352.6221	3186.4224	3195.0756
Frequencies --	3204.4167	3212.0121	3215.8575

scf done: -400.626567

C	-0.000000	0.000000	4.773319
N	-0.000000	0.000000	3.586563
C	-0.000000	0.000000	2.276508
C	-0.000000	0.000000	1.060823
C	0.000000	0.000000	-0.365106
C	0.000000	1.216685	-1.077571
C	-0.000000	-1.216685	-1.077571
C	0.000000	1.210744	-2.471097
H	0.000000	2.153793	-0.530685
C	0.000000	-1.210744	-2.471097
H	-0.000000	-2.153793	-0.530685
C	0.000000	-0.000000	-3.171076
H	0.000000	2.152280	-3.011799
H	0.000000	-2.152280	-3.011799
H	0.000000	-0.000000	-4.256895

5a (C3H3N1) B3LYP/6-31+G(d,p)

Frequencies --	217.8132	295.8095	528.0781
Frequencies --	719.5952	901.1454	946.1530
Frequencies --	983.5235	1137.6453	1333.3929
Frequencies --	1436.4406	1689.1304	**2202.5333***
Frequencies --	3180.3163	3201.3481	3279.1508

scf done: -170.813237

C	-0.023951	-0.016613	4.905548
N	0.019161	0.013286	3.727093
C	0.013765	0.009550	2.345585
C	0.930516	0.644700	1.609913
H	-0.801854	-0.555530	1.903904
H	0.873912	0.605488	0.528417
H	1.739399	1.205114	2.065619

3a (C5H9N1) B3LYP/6-31+G(d,p)

Frequencies --	92.6690	111.3773	127.6194
Frequencies --	245.0046	261.2594	301.2434
Frequencies --	379.3017	480.2636	742.8900
Frequencies --	802.8715	905.8112	945.5969
Frequencies --	955.3587	1024.5825	1060.6066
Frequencies --	1131.7953	1175.6486	1256.1675
Frequencies --	1294.4375	1324.7157	1329.8177
Frequencies --	1378.1389	1406.8112	1421.8151
Frequencies --	1494.8010	1495.8061	1505.3122
Frequencies --	1505.6328	1516.7190	**2232.5307***
Frequencies --	3018.8156	3034.9757	3042.0136
Frequencies --	3048.0476	3052.0079	3078.1727
Frequencies --	3099.1481	3105.4124	3109.1125

scf done: -250.685681

C	-0.053176	-0.024892	4.957521
N	-0.041054	-0.018780	3.782317
C	-0.009955	-0.004803	2.353674
C	1.317563	0.540231	1.807754
H	-0.849832	0.606080	2.003443
H	-0.179136	-1.029000	2.002066
C	1.344290	0.553205	0.274166
H	1.470940	1.554280	2.196497
H	2.139054	-0.075077	2.194387
C	2.662493	1.094495	-0.290993
H	1.176429	-0.465169	-0.102400
H	0.509437	1.161425	-0.100202
H	2.654115	1.092331	-1.385801
H	2.841944	2.123998	0.039363
H	3.513401	0.486799	0.037453

1d B3LYP/6-31+G(d,p) scrf(pcm,Solvent=methanol)

SCF Done: E(RB3LYP) = -400.631587077

C	0.000000	0.000000	4.791100
N	0.000000	0.000000	3.606700
C	0.000000	0.000000	2.296600
C	0.000000	0.000000	1.080600
C	0.000000	0.000000	-0.345000
C	0.000000	1.218900	-1.055400
C	0.000000	-1.218900	-1.055400
C	0.000000	1.212100	-2.449500
H	0.000000	2.156700	-0.510000
C	0.000000	-1.212100	-2.449500
H	0.000000	-2.156700	-0.510000
C	0.000000	0.000000	-3.148800
H	0.000000	2.153200	-2.990400
H	0.000000	-2.153200	-2.990400
H	0.000000	0.000000	-4.234500

1l B3LYP/6-31+G(d,p) scrf(pcm,Solvent=methanol)

SCF Done: E(RB3LYP) = -516.416982868

C	-3.392200	0.731200	0.000000
N	-2.345400	1.479200	0.000000
C	-1.109400	0.963100	0.000100
C	0.068100	0.640400	0.000100
C	1.433700	0.222100	0.000000
C	1.769500	-1.149900	0.000100
C	2.477400	1.173500	-0.000100
C	3.104900	-1.552300	0.000100
H	0.975100	-1.889600	0.000100
C	3.810500	0.763000	-0.000100
H	2.230800	2.230600	-0.000100
C	4.130200	-0.599500	0.000000
H	3.346100	-2.611300	0.000100
H	4.601200	1.507500	-0.000200
H	5.168900	-0.916100	-0.000100
O	-3.349100	-0.602300	0.000000
C	-4.622000	-1.294100	-0.000100
H	-5.189300	-1.039600	0.898300
H	-5.189000	-1.039800	-0.898800
H	-4.379600	-2.355200	0.000100
H	-4.377600	1.193900	0.000000

8c B3LYP/6-31+G(d,p) scrf(pcm,Solvent=methanol)

SCF Done: E(RB3LYP) = -400.675250114

N	0.000000	0.000000	4.802500
C	0.000000	0.000000	3.632400
C	0.000000	0.000000	2.267600

C	0.000000	0.000000	1.047800
C	0.000000	0.000000	-0.373900
C	0.000000	1.221500	-1.081100
C	0.000000	-1.221500	-1.081100
C	0.000000	1.213500	-2.474600
H	0.000000	2.158600	-0.535100
C	0.000000	-1.213500	-2.474600
H	0.000000	-2.158600	-0.535100
C	0.000000	0.000000	-3.171900
H	0.000000	2.153600	-3.016800
H	0.000000	-2.153600	-3.016800
H	0.000000	0.000000	-4.257400

9a B3LYP/6-31+G(d,p) scrf(pcm,Solvent=methanol)

SCF Done: E(RB3LYP) = -347.750632783 A.U. after 8 cycles

C	0.000100	2.598700	0.000000
C	0.000100	1.384900	0.000000
C	0.000100	-0.047300	0.000000
C	0.000100	-0.761400	1.215800
C	0.000100	-0.761400	-1.215800
C	0.000100	-2.156700	1.210700
H	0.000100	-0.216000	2.153900
C	0.000100	-2.156700	-1.210700
H	0.000100	-0.216000	-2.153900
C	0.000100	-2.857900	0.000000
H	0.000100	-2.697400	2.152400
H	0.000100	-2.697400	-2.152400
H	0.000100	-3.943800	0.000000
C	0.000100	4.138700	0.000000
H	-0.506400	4.495300	0.872400
H	1.008900	4.495300	0.000000
H	-0.506400	4.495300	-0.872400

10a B3LYP/6-31+G(d,p) scrf(pcm,Solvent=methanol)

SCF Done: E(RB3LYP) = -1031.13456831

N	-0.051500	-2.014500	-0.406200
C	-1.190500	-1.334200	-0.269200
C	-2.290700	-0.817200	-0.160500
C	-3.566100	-0.194400	-0.029500
C	-3.669100	1.188900	0.230000
C	-4.750200	-0.951500	-0.157100
C	-4.918200	1.791700	0.357300
H	-2.762300	1.777600	0.329500
C	-5.995300	-0.340500	-0.028700
H	-4.679000	-2.016300	-0.356700
C	-6.084200	1.030900	0.228600
H	-4.982800	2.857500	0.557300

H	-6.898800	-0.935300	-0.129500
H	-7.056900	1.504200	0.328300
H	1.997100	-2.069500	-0.446400
S	1.399600	0.264800	-0.038200
C	3.194900	0.295100	0.033700
C	3.880700	-0.361700	1.063900
C	3.898100	1.031700	-0.927400
C	5.274200	-0.297600	1.114300
H	3.330900	-0.913400	1.820200
C	5.290500	1.105200	-0.857300
H	3.361200	1.538000	-1.723400
C	5.979400	0.437600	0.158100
H	5.806100	-0.813200	1.908600
H	5.835500	1.677200	-1.602700
H	7.063100	0.492300	0.206100

1d CBS-QB3

Temperature= 298.150000 Pressure= 1.000000
E(ZPE)= 0.107148 E(Thermal)= 0.115458
E(SCF)= -398.191874 DE(MP2)= -1.543674
DE(CBS)= -0.143603 DE(MP34)= -0.038217
DE(CCSD)= -0.066153 DE(Int)= 0.049971
DE(Empirical)= -0.065939
CBS-QB3 (0 K)= -399.892341 CBS-QB3 Energy= -399.884031
CBS-QB3 Enthalpy= -399.883087 CBS-QB3 Free Energy= -399.925406

C	-0.000000	0.000000	2.251214
C	-0.000000	0.000000	1.039965
C	0.000000	0.000000	-0.380522
C	0.000000	1.214776	-1.089064
C	-0.000000	-1.214776	-1.089064
C	0.000000	1.208185	-2.478025
H	0.000000	2.149280	-0.541960
C	0.000000	-1.208185	-2.478025
H	-0.000000	-2.149280	-0.541960
C	0.000000	0.000000	-3.174369
H	0.000000	2.147181	-3.018957
H	0.000000	-2.147181	-3.018957
H	0.000000	0.000000	-4.258222
N	-0.000000	-0.000000	4.775220
C	-0.000000	-0.000000	3.615357

8c CBS-QB3

Temperature= 298.150000 Pressure= 1.000000
E(ZPE)= 0.108018 E(Thermal)= 0.116122
E(SCF)= -398.228063 DE(MP2)= -1.558838
DE(CBS)= -0.144032 DE(MP34)= -0.030148
DE(CCSD)= -0.066892 DE(Int)= 0.050321
DE(Empirical)= -0.065909

CBS-QB3 (0 K)= -399.935544 CBS-QB3 Energy= -399.927439
CBS-QB3 Enthalpy= -399.926495 CBS-QB3 Free Energy= -399.968547

C	-0.000000	0.000000	4.755088
N	-0.000000	0.000000	3.572725
C	-0.000000	0.000000	2.266071
C	-0.000000	0.000000	1.058003
C	0.000000	0.000000	-0.365152
C	0.000000	1.213006	-1.076188
C	-0.000000	-1.213006	-1.076188
C	0.000000	1.207187	-2.465541
H	0.000000	2.148110	-0.529888
C	0.000000	-1.207187	-2.465541
H	-0.000000	-2.148110	-0.529888
C	0.000000	0.000000	-3.163226
H	0.000000	2.146823	-3.005671
H	-0.000000	-2.146823	-3.005671
H	0.000000	-0.000000	-4.247104

References

[1] Bürger, H.; Lentz, D.; Meisner, B.; Nickelt, N.; Preugschat, D.; Senzlober, M. Syntheses and ^{1}H-, ^{13}C-, and ^{15}N NMR Spectra of Ethynyl Isocyanide, H−C≡C−N≡C, D−C≡C−N≡C, and Prop-1-ynyl Isocyanide, H_3C−C≡C−N≡C, D_3C−C≡C−N≡C: High-Resolution Infrared Spectrum of Prop-1-ynyl Isocyanide. *Chem. Eur. J.* **2000**, *6*, 3377-3385.

[2] Chao, A.; Alwedi, E.; Fleming, F. F. Isocyanide Purification: C-2 Silica Cleans Up a Dirty Little Secret *Synthesis* **2019,** *51*, 2122-2127.

[3] Massarotti, A.; Brunelli, F.; Aprile, S.; Giustiniano, M.; Tron, G. C. Medicinal Chemistry of Isocyanides. *Chem. Rev.* **2021,** *121*, 10742–10788.

[4] Pirrung, M. C.; Ghorai, S. Versatile, Fragrant, Convertible Isonitriles. *J. Am. Chem. Soc.* **2006,** *128,* 11772–11773.

[5] D. J. White, R. P. Blakemore, J. N. Green, E. Bryan Hauser, A. M. Holoboski, E. L. Keown, S. C. Nylund Kolz, W. B. Phillips, Total Synthesis of Rhizoxin D, a Potent Antimitotic Agent from the Fungus *Rhizopus chinensis*, *J. Org. Chem.* **2002**, *67*, 7750–7760.

[6] Tian, H.; Holyoke, C. W., Jr.; Fleming, F. F. Stereoselective Synthesis of (E)- and (Z)-Isocyanoalkenes. *Org. Lett*. **2022**, *24*, 8657-8661.

[7] Tian, H.; Marrazzo, J.-P. R.; Huynh, T.; Fleming, F. F. Thiazoles via Formal [4+1] of NaSH to Bromoisocyanoalkenes. *J. Org. Chem.* **2025**, *90,* 4513-4517.

[8] (a) V. G. Nenajdenko, A. V. Gulevich, N. V. Sokolova, A. V. Mironov, E. S. Balenkova, Chiral Isocyanoazides: Efficient Bifunctional Reagents for Bioconjugation, *Eur. J. Org. Chem.* **2010**, 1445–1449. (b) K. F. McClure, Y. A. Abramov, E. R. Laird, J. T. Barberia, W. Cai, T. J. Carty, S. R. Cortina, D. E. Danley, A. J. Dipesa, K. M. Donahue, M. A. Dombroski, N. C. Elliott, C. A. Gabel, S. Han, T. R. Hynes, P. K. LeMotte, M. N. Mansour, E. S. Marr, M. A. Letavic, J. Pandit, D. B. Ripin, F. J. Sweeney, D. Tan, Y. Tao, Theoretical and Experimental Design of Atypical Kinase Inhibitors: Application to p38 MAP Kinase, *J. Med. Chem.* **2005**, *48*, 5728–5737. (c) S. Kotha, N. Sreenivasachary, Synthesis of constrained α-amino acid derivatives via ring-closing metathesis, *Bioorg. Med. Chem. Lett.* **1998**, *8*, 257–260.

[9] T. Brinck, J. H. Stenlid. The molecular surface property approach: a guide to chemical interactions in chemistry,

medicine, and material science. *Adv. Theory Simul.* **2019**, *2,* 1800149.

[10] T. Brinck. Molecular electrostatic potential as a guide to intermolecular interactions: challenge of nucleophilic interaction sites. *Pure Appl. Chem.* **2025**, *97*, 1675-1684.

[11] P. Sjoberg, J. S. Murray, T. Brinck, P. Politzer. Average local ionization energies on the molecular-surfaces of aromatic systems as guides to chemical-reactivity. *Can. J. Chem.* **1990**, *68*, 1440-1443.

[12] T. Brinck, P. Carlqvist, J. H. Stenlid. Local Electron Attachment Energy and Its Use for Predicting Nucleophilic Reactions and Halogen Bonding. *J. Phys. Chem. A* **2016**, *120*, 10023-10032.

[13] Stenlid, J. H.; Brinck, T. Nucleophilic aromatic substitution reactions described by the local electron attachment energy. *J. Org. Chem.* **2017**, *82*, 3072-3083.

[14] Jorner, K.; Brinck, T.; Norrby, P.-O.; Buttar, D. Machine learning meets mechanistic modelling for accurate prediction of experimental activation energies. *Chem. Sci.* **2021**, *12*, 1163-1175.

[15] Frisch, M. J. et al. *Gaussian 16 Suite of Programs* (Gaussian, Inc., Wallingford CT, 2016).

[16] NIST Computational Chemistry Comparison and Benchmark Database, NIST Standard Reference Database Number 101 Release 22, May 2022, Editor: Russell D. Johnson III http://cccbdb.nist.gov/DOI:10.18434/T47C7Z.

[17] E. F. Pettersen, T. D. Goddard, C. C. Huang, G. S. Couch, D. M. Greenblatt, E. C. Meng, T. E. Ferrin. UCSF Chimera—a visualization system for exploratory research and analysis. *J. Comp. Chem.* **2004**, *25*, 1605-1612.

[18] J. A. Montgomery, Jr., M. J. Frisch, J. W. Ochterski, G. A. Petersson. A complete basis set model chemistry. VI. Use of density functional geometries and frequencies. *J. Chem. Phys.* **1999**, *110*, 2822-2827.
